# Genome-wide analysis of the bZIP gene family in Chinese jujube (*Ziziphus jujuba Mill.*)

**DOI:** 10.1186/s12864-020-06890-7

**Published:** 2020-07-14

**Authors:** Yao Zhang, Weilin Gao, Hongtai Li, Yongkang Wang, Dengke Li, Chaoling Xue, Zhiguo Liu, Mengjun Liu, Jin Zhao

**Affiliations:** 1 College of Life Science, Hebei Agricultural University, Baoding, China; 2 Hebei Key Laboratory of Plant Physiology and Molecular Pathology, Hebei Agricultural University, Baoding, China; 3 Pomology Institute, Shanxi Academy of Agricultural Sciences, Taigu, China; 4 Research Center of Chinese Jujube, Hebei Agricultural University, Baoding, China

**Keywords:** *ZjbZIPs*, Chinese jujube, Tissue-specific expression, Fruit development, Phytoplasma, Abiotic stress, Protein-protein interaction

## Abstract

**Background:**

Among several TF families unique to eukaryotes, the basic leucine zipper (bZIP) family is one of the most important. Chinese jujube (*Ziziphus jujuba* Mill.) is a popular fruit tree species in Asia, and its fruits are rich in sugar, vitamin C and so on. Analysis of the bZIP gene family of jujube has not yet been reported. In this study, *ZjbZIP*s were identified firstly, their expression patterns were further studied in different tissues and in response to various abiotic and phytoplasma stresses, and their protein-protein interactions were also analyzed.

**Results:**

At the whole genome level, 45 *ZjbZIPs* were identified and classified into 14 classes. The members of each class of bZIP subfamily contain a specific conserved domain in addition to the core bZIP conserved domain, which may be related to its biological function. Relative Synonymous Codon Usage (RSCU) analysis displayed low values of NTA and NCG codons in *ZjbZIP*s, which would be beneficial to increase the protein production and also indicated that *ZjbZIP*s were at a relative high methylation level. The paralogous and orthologous events occurred during the evolutionary process of *ZjbZIPs*. Thirty-four ZjbZIPs were mapped to but not evenly distributed among 10 pseudo- chromosomes. 30 of *ZjbZIP* genes showed diverse tissue-specific expression in jujube and wild jujube trees, indicating that these genes may have multiple functions. Some *ZjbZIP* genes were specifically analyzed and found to play important roles in the early stage of fruit development. Moreover, some *ZjbZIP*s that respond to phytoplasma invasion and abiotic stress environmental conditions, such as salt and low temperature, were found. Based on homology comparisons, prediction analysis and yeast two-hybrid, a protein interaction network including 42 ZjbZIPs was constructed.

**Conclusions:**

The bioinformatics analyses of 45 *ZjbZIPs* were implemented systematically, and their expression profiles in jujube and wild jujube showed that many genes might play crucial roles during fruit ripening and in the response to phytoplasma and abiotic stresses. The protein interaction networks among ZjbZIPs could provide useful information for further functional studies.

## Background

Transcription factors (TFs) are an important component of regulatory networks. TFs bind to specific promoter sequences to activate or inhibit the expression of target genes [1]. Among several TF families unique to eukaryotes, the basic leucine zipper (bZIP) family is one of the largest and most diverse [2–4], containing two regions with different functions: the basic region and the leucine zipper region [5]. The basic region is highly conserved and consists of approximately 16 amino acid residues with a consistent N-X7-R/K motif for nuclear localization and sequentially specific DNA binding, while the leucine zipper region is less conserved and forms a helical structure with dimerization specificity [1, 6].

Members of the bZIP family are involved in the regulation of plant resistance under biotic and abiotic stresses [7–9], and play some important roles during plant growth and development processes, such as hormone signal transduction [10], energy metabolism [11], seedling development [12] and flowering [13]. In plant, bZIPs can be combined with cis- acting elements such as G-box (CACGTG), A-box (TACGTA) and abscisic acid (ABA)- responsive elements (ABRE) (CCACGTGG) to regulate the expression of downstream genes. In Arabidopsis, ABF1, ABF2, ABF3 and ABF4 could bind to the cis-acting element ABRE, and regulate many downstream salt and drought tolerances through the interaction between ABRE and bZIP proteins [14]. OsbZIP1 might enhance resistance to *Magnaporthe grisea* through salicylic acid (SA), jasmonic acid (JA) and ABA signal transduction pathways [15]. In addition, AtbZIPs also regulated the signal transduction of ABA-related pathways, thereby affecting seed germination and maturity [12].

The TGA (TGACG motif-binding factor) subfamily of bZIPs plays important roles in defense responses against pathogens [16]. As the target of SA signaling, the TGA factors can thus activate and connect the SA pathway with the JA/ET-dependent pathway [17–20]. Moreover, these WRKY transcription factors induced by SA could activate the promoter of pathogenesis-related (PR)-1 and involved in the regulation of the TGA/NPR1 complex [21, 22]. And they also demonstrated some reverse functions, such as TGA2 suppressed the expression of *PR1* whereas TGA6 was able to increase *PR1* expression and could induce basal defense [23]. Thus, such interactions will be discovered in more plants when further exploring the function of TGA factors.

Chinese jujube (*Ziziphus jujuba* Mill.), a member of the Rhamnaceae family, is an important dry fruit and a traditional herbal medicine in Asia [24]. Both Chinese jujube and wild jujube, which are considered ideal fruit trees in arid and semiarid temperate regions, have strong tolerance to biotic and abiotic stress [25, 26]. Although the bZIP gene family has been analyzed in many other plant species such as Arabidopsis [27], peach [28], apple [3] and so on [29, 30], the analysis of bZIP family in jujube has not yet been reported. Based on the functions of bZIPs in other species, we thought the members of bZIP family should have multiple functions on jujube development, defense responses against pathogens and abiotic stress. Thus, the characteristics and expressions of bZIP members in Chinese jujube are identified and analyzed systematically. These results would provide a basis for future studies related to biological functions and the regulatory networks.

## Results

### Identification of *ZjbZIP*s in Chinese jujube

A total of 45 nonredundant putative bZIP transcripts (Table 1) within the jujube genome sequence were identified. They were named *ZjbZIP1* to *ZjbZIP45* according to their gene structure and motifs. The ORF length of the *ZjbZIPs* ranged from 384 bp (*ZjbZIP44*) to 2205 bp (*ZjbZIP11*), and they encoded proteins ranging from 127 (*ZjbZIP44*) to 749 (*ZjbZIP11*) amino acids (aa) in length, with predicted pIs ranging from 4.65 (*ZjbZIP40*) to 9.96 (*ZjbZIP44*) (Table 1) and predicted molecular weights (MWs) of 14.67–82.15 kDa. The proteins with an isoelectric point greater than 7 accounted for 53% of the total number, which means that half of the ZjbZIP proteins were neutral or alkaline, and most of the proteins in the E, F and G subfamilies were weakly acidic.

**Table 1:** The information of bZIP gene family in Chinese jujube

	NCBI accession	Group	Chr.	Size (aa)	MW(D)	PI	ORF (bp)	Exon number
*ZjbZIP1*	XM_016043261.2	A	12	436	47,951.52	8.47	1311	4
*ZjbZIP2*	XM_016043231.2	A	12	270	30,201.81	8.89	813	3
*ZjbZIP3*	XM_016020783.2	A	3	321	35,584.09	8.5	966	3
*ZjbZIP4*	XM_016030823.2	A	6	299	32,803.72	7.79	900	4
*ZjbZIP5*	XM_016030951.2	A	6	313	33,876.96	9.09	942	4
*ZjbZIP6*	XM_016032146.2	A	7	325	35,538.02	5.51	978	3
*ZjbZIP7*	XM_016017070.2	A	2	397	43,940.78	7.09	1194	4
*ZjbZIP8*	XM_016034266.2	A	8	265	29,175.47	9.26	798	3
*ZjbZIP9*	XM_016037430.2	A	9	434	46,121.77	9.85	1305	4
*ZjbZIP10*	XM_025074624.1	A	6	425	45,632.07	9.21	1278	4
*ZjbZIP11*	XM_016042475.2	B	12	749	82,145.02	7.07	2250	2
*ZjbZIP12*	XM_016036011.2	C	9	449	48,518.82	6.2	1350	6
*ZjbZIP13*	XM_016019943.2	C	2	437	47,207	5.91	1314	6
*ZjbZIP14*	XM_016017317.2	D	2	515	57,096.85	7.37	1548	12
*ZjbZIP15*	XM_016010890.2	D	UN	467	51,490.68	7.09	1404	11
*ZjbZIP16*	XM_016040197.2	D	10	468	51,929.31	6.07	1407	11
*ZjbZIP17*	XM_016035424.2	D	8	363	41,012.5	7.07	1092	8
*ZjbZIP18*	XM_025069094.1	D	UN	488	47,387.8	7.32	1269	11
*ZjbZIP19*	XM_025076058.1	D	7	348	39,084.03	6.09	1047	8
*ZjbZIP20*	XM_016039294.2	D	10	396	44,113.28	6.63	1191	9
*ZjbZIP21*	XM_016043667.2	E	12	343	38,339.63	6.25	1032	4
*ZjbZIP22*	XM_016037719.2	F	9	272	29,467.89	6.21	819	2
*ZjbZIP23*	XM_016026428.2	F	5	264	29,307.66	5.69	795	1
*ZjbZIP24*	XM_016037896.2	F	9	272	29,467.89	6.21	819	2
*ZjbZIP25*	XM_016010135.2	G	UN	409	43,539.79	5.97	1230	12
*ZjbZIP26*	XM_016033348.2	G	8	427	45,943.89	6.54	1284	12
*ZjbZIP27*	XM_016034676.2	G	8	359	37,885.71	5.78	1080	11
*ZjbZIP28*	XM_016044679.2	H	12	200	22,598.46	9.69	603	4
*ZjbZIP29*	XM_016030371.2	H	6	167	18,168.02	9.64	504	4
*ZjbZIP30*	XM_025069252.1	H	UN	177	19,743.88	9.24	534	4
*ZjbZIP31*	XM_025077206.1	I	9	589	64,322.77	6.35	1770	4
*ZjbZIP32*	XM_016022085.2	I	3	386	42,154.4	5.85	1161	4
*ZjbZIP33*	XM_016019180.2	I	2	470	49,855.63	6.24	1413	4
*ZjbZIP34*	XM_016012104.2	I	UN	427	46,237.98	6.09	1284	4
*ZjbZIP35*	XM_016010308.1	I	UN	339	36,967.19	9.45	1020	4
*ZjbZIP36*	XM_016036218.1	S	9	146	16,518.78	8.11	441	1
*ZjbZIP37*	XM_016016004.2	S	UN	229	25,550.97	9.06	690	1
*ZjbZIP38*	XM_016013151.2	S	UN	146	16,504.75	8.11	441	1
*ZjbZIP39*	XM_016024994.2	S	4	196	22,785.29	6.13	591	1
*ZjbZIP40*	XM_016024530.2	L	4	307	34,443.98	4.65	924	3
*ZjbZIP41*	XM_016013429.2	C	UN	349	38,379.34	5.49	1050	6
*ZjbZIP42*	XM_016023464.2	K	4	373	41,886.53	8.33	1122	4
*ZjbZIP43*	XM_016011524.2	M	UN	364	40,092.99	6.05	1095	11
*ZjbZIP44*	XM_016027660.1	M	5	127	14,671.01	9.96	384	1
*ZjbZIP45*	XM_016046481.2	J	UN	518	57,828.84	8.98	1557	1

The average GC content of 45 *ZjbZIP*s was 46.88%, and the contents of GC_1_, GC_2_ and GC_3_ were 53.45, 44.63 and 42.56%, respectively. Relative Synonymous Codon Usage (RSCU) analyses will help us to understand the patterns in *ZjbZIP*s, and the RSCU values greater than 1.5 was defined as high-frequency codons [31]. Among the 64 codons of 45 *ZjbZIP*s, seven high-frequency codons (AGG 1.80, AGA 1.79, GTT 1.73, GCT 1.69, TTG 1.65, TCT 1.59 and TTT 1.58) were investigated, and most of them were T-ended (Table 2). We also found that most *ZjbZIP*s prefer ATG as the stop codon. RSCU values of four NCG codons in *ZjbZIP*s were the lowest (TCG 0.62, CCG 0.61, ACG 0.45, GCG 0.36) (Table 2), suggesting that *ZjbZIP*s were at a relative high methylation level [32]. Meanwhile, RSCU values of four NTA codons also displayed a lower level (ATA 0.75, TTA 0.73, GTA 0.66, CTA 0.55), which was beneficial to increase the protein production by inhibiting mRNA degradation [33].

**Table 2:** The RSCU of 64 codons in *ZjbZIPs*

First codon	Second codon				Third codon
	T	C	A	G	
T	**TTT(1.58)**	**TCT(1.59)**	TAT(1.43)	TGT(1.19)	T
	TTC(0.42)	TCC(0.92)	TAC(0.57)	TGC(0.81)	C
	TTA(0.73)	TCA(1.35)	TAA(0.60)	TGA(1.40)	A
	**TTG(1.65)**	TCG(0.62)	TAG(1.00)	TGG(1.00)	G
C	CTT(1.46)	CCT(1.47)	CAT(1.25)	CGT(0.69)	T
	CTC(0.76)	CCC(0.55)	CAC(0.75)	CGC(0.49)	C
	CTA(0.55)	CCA(1.36)	CAA(0.97)	CGA(0.68)	A
	CTG(0.85)	CCG(0.61)	CAG(1.03)	CGG(0.56)	G
A	ATT(1.37)	ACT(1.38)	AAT(1.23)	AGT(0.86)	T
	ATC(0.88)	ACC(0.98)	AAC(0.77)	AGC(0.66)	C
	ATA(0.75)	ACA(1.19)	AAA(0.90)	**AGA(1.79)**	A
	ATG(1.00)	ACG(0.45)	AAG(1.10)	**AGG(1.80)**	G
G	**GTT(1.73)**	**GCT(1.69)**	GAT(1.34)	GGT(1.35)	T
	GTC(0.61)	GCC(0.72)	GAC(0.66)	GGC(0.59)	C
	GTA(0.66)	GCA(1.22)	GAA(0.97)	GGA(1.34)	A
	GTG(1.00)	GCG(0.36)	GAG(1.03)	GGG(0.72)	G

### Phylogenetic tree construction and conserved motifs of ZjbZIPs

**Fig. 1:**
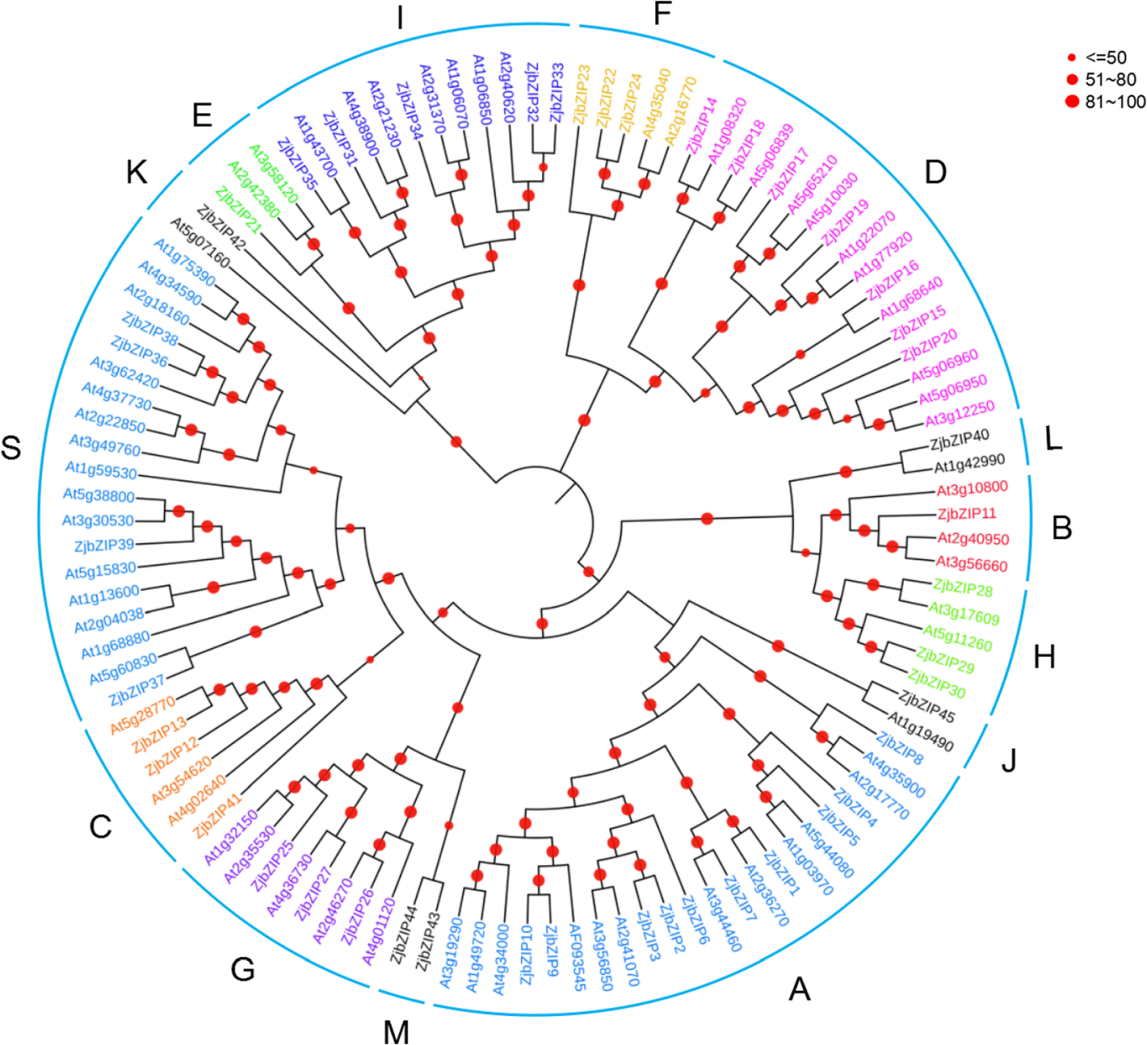
The phylogenetic analysis of ZjbZIPs. The NJ tree was constructed from the protein sequences of ZjbZIPs using MEGA7 with 1000 bootstrap copies

Compared with bZIPs in Arabidopsis, ZjbZIPs were also divided into 10 subfamilies (A-I, S). In addition, we defined six newly discovered ZjbZIPs as four subfamilies of J, K, L and M (Fig. 1). The classification result was further supported by the phylogenetic tree of bZIPs between jujube and apple (Additional file 1). In jujube, the A subfamily is the largest subfamily, while in the Arabidopsis, the S subfamily is the largest subfamily [34]. As expected, bZIP proteins from the same group tended to cluster together and were named following the same scheme.

**Fig. 2:**
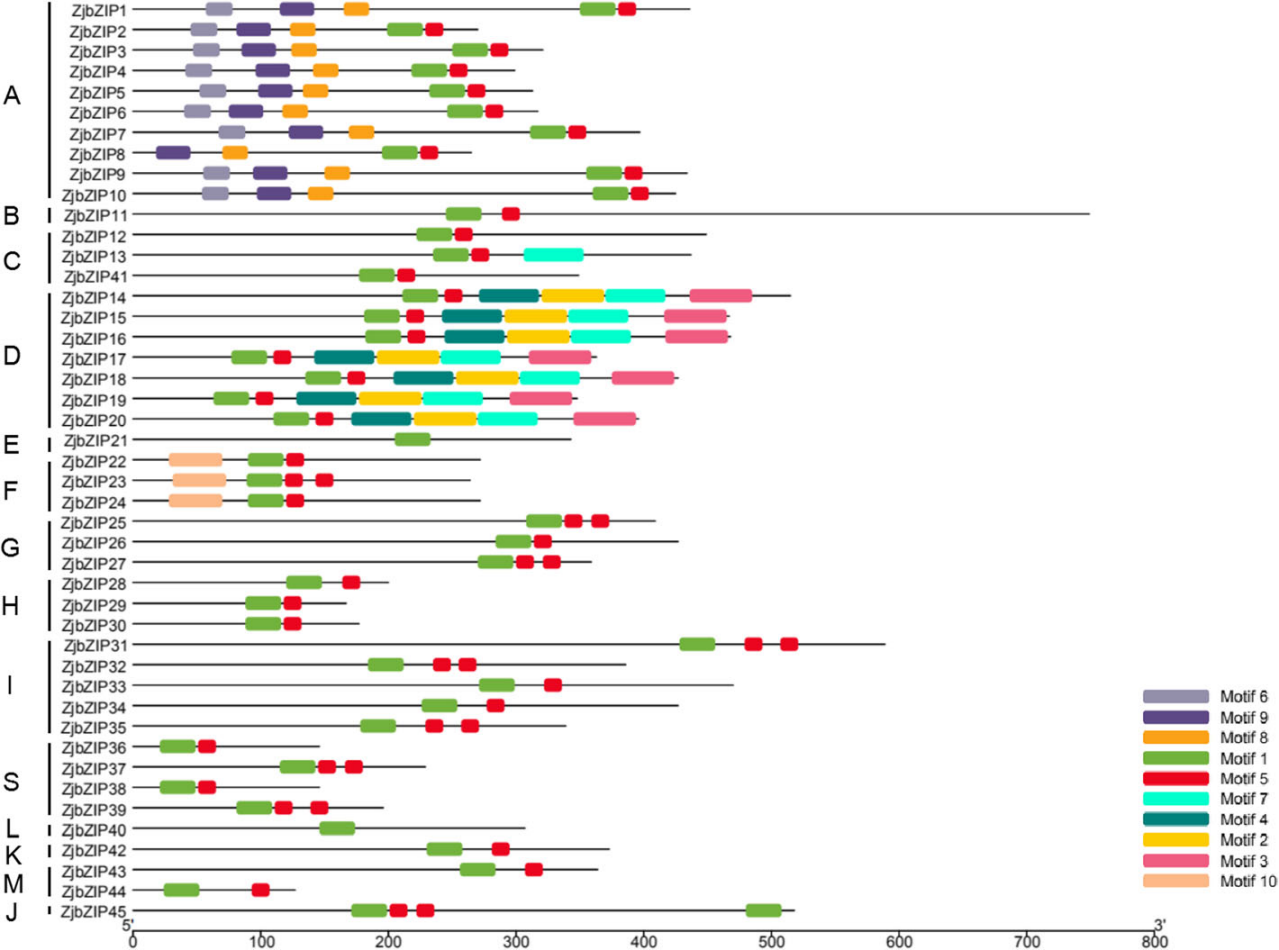
Conserved motifs of ZjbZIP proteins. The motif in the ZjbZIP proteins was identified by using Multiple Em for Motif Elicitation (MEME). Ten conserved motifs were identified and displayed in different colors

Using MEME software, a total of 10 conserved motifs among ZjbZIPs was identified (Fig. 2), of which motif 1 and motif 5 were identified as the core conserved domains and constituted the leucine zipper region of bZIP (Additional file 2). The proteins in each subfamily contain the same conserved motifs, which further support the above result of phylogenetic tree. However, they also have different conserved motifs among various subfamilies. For example, in addition to the core conserved domains, the A subfamily also contains three conserved motifs (Motif 6, 8 and 9), which may be related to their different biological functions.

### The phylogenetic tree and line charts for a lineage of gene groups for *ZjbZIP* genes

In order to further study the evolution pattern and direction of *ZjbZIP* genes, *ZjbZIP26* and *29* were selected to perform evolutionary analysis. *ZjbZIP26* and *ZjbZIP29* are homologous genes of *GBF3* and *HY5*, respectively. And *GBF3* and *HY5* were proved to participate in various biological processes [35, 36].

**Fig. 3:**
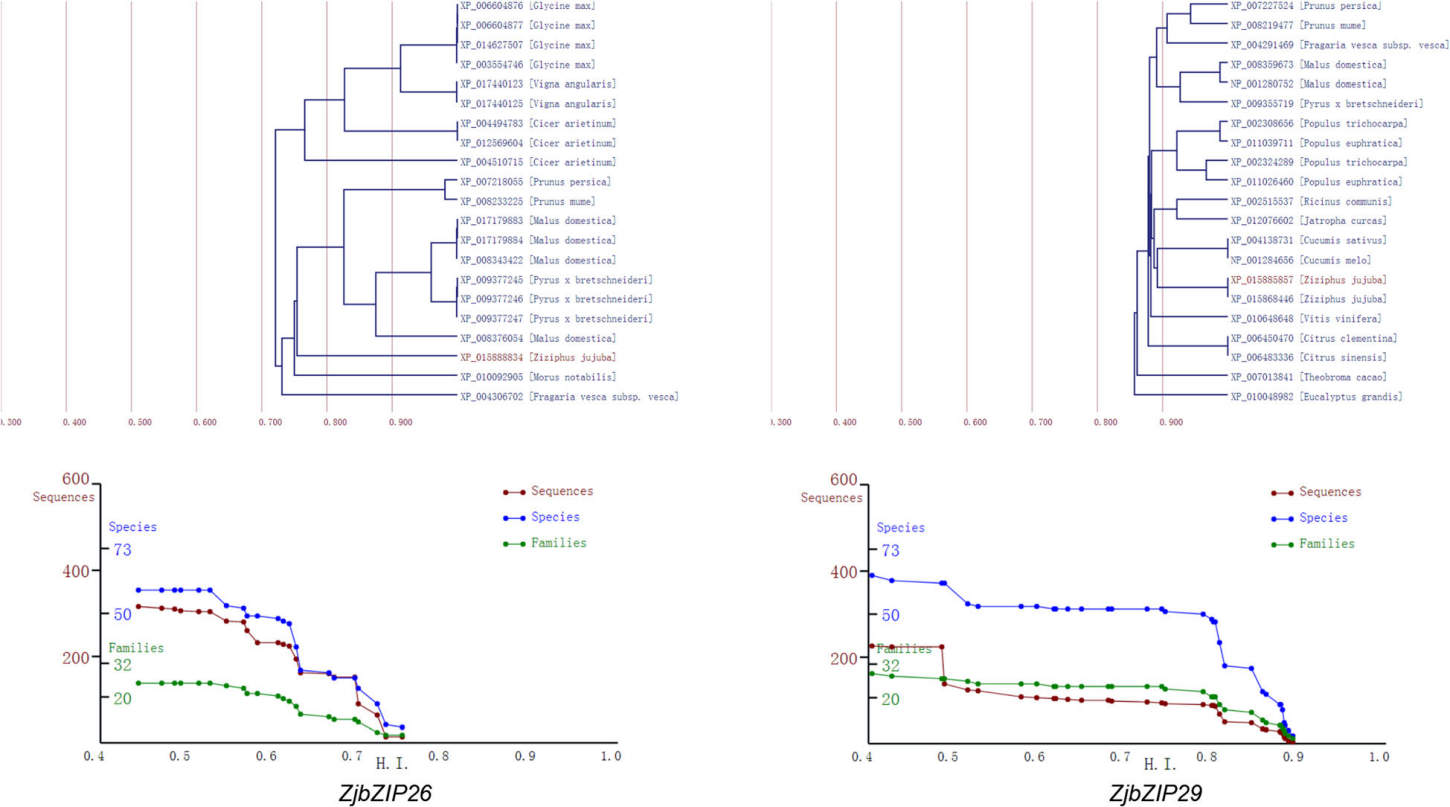
The phylogenetic trees and line charts for a lineage of gene groups for *ZjbZIP26* and *ZjbZIP29*. The tree contains *ZjbZIP26* (*ZjbZIP29*) and 20 other genes with the highest *HI* values. The horizontal axis represents *HI*. Red, blue, and green lines represent the numbers of genes (sequences), species, and families contained in individual gene groups, respectively

As shown in the Fig. 3, two phylogenetic trees of *ZjbZIP26* and *− 29* with 20 other genes showing high homology indices (*HIs*) in various species were constructed, respectively. The values of *HIs* between all pairs in the two trees were all above 0.7, suggesting that they have similar amino acid sequences and might have conserved functions. To the phylogenetic tree of ZjbZIP29, three paralogous events were presumably occurred in a group of two genes (*Cucumis sativus* XP_004138731 and *Cucumis melo* NP_001284656), a group of two genes (*Citrus clementina* XP_006450470 and *Citrus sinensis* XP_006483336) and a group of two genes (*Ziziphus jujuba* XP_015885857 and XP_015868446) in *Ziziphus jujuba*. To the phylogenetic tree of *ZjbZIP26*, there were also five paralogous events.

Generally, along with evolutionary time the decrease of both the numbers of genes and species represents an orthologous event, and the number of genes decreases and the number of species is retained, which means a paralogous event [37]. For *ZjbZIP26*, along with evolutionary time, the numbers of genes (red line) and species (blue line) were both decreased in the timing between 0.619 and 0.632 of HIs, suggesting that an orthologous event happened; and only the numbers of genes (red line) were decreased in the timing between 0.570 and 0.582 of HIs, indicating that a paralogous event occurred. The two kinds of homologous events also found in *ZjbZIP29* analysis*.* Therefore, the paralogous and orthologous duplication events should occurred during the evolutionary process of *bZIPs*.

### The chromosomal location and gene structure of *ZjbZIP*s

**Fig. 4:**
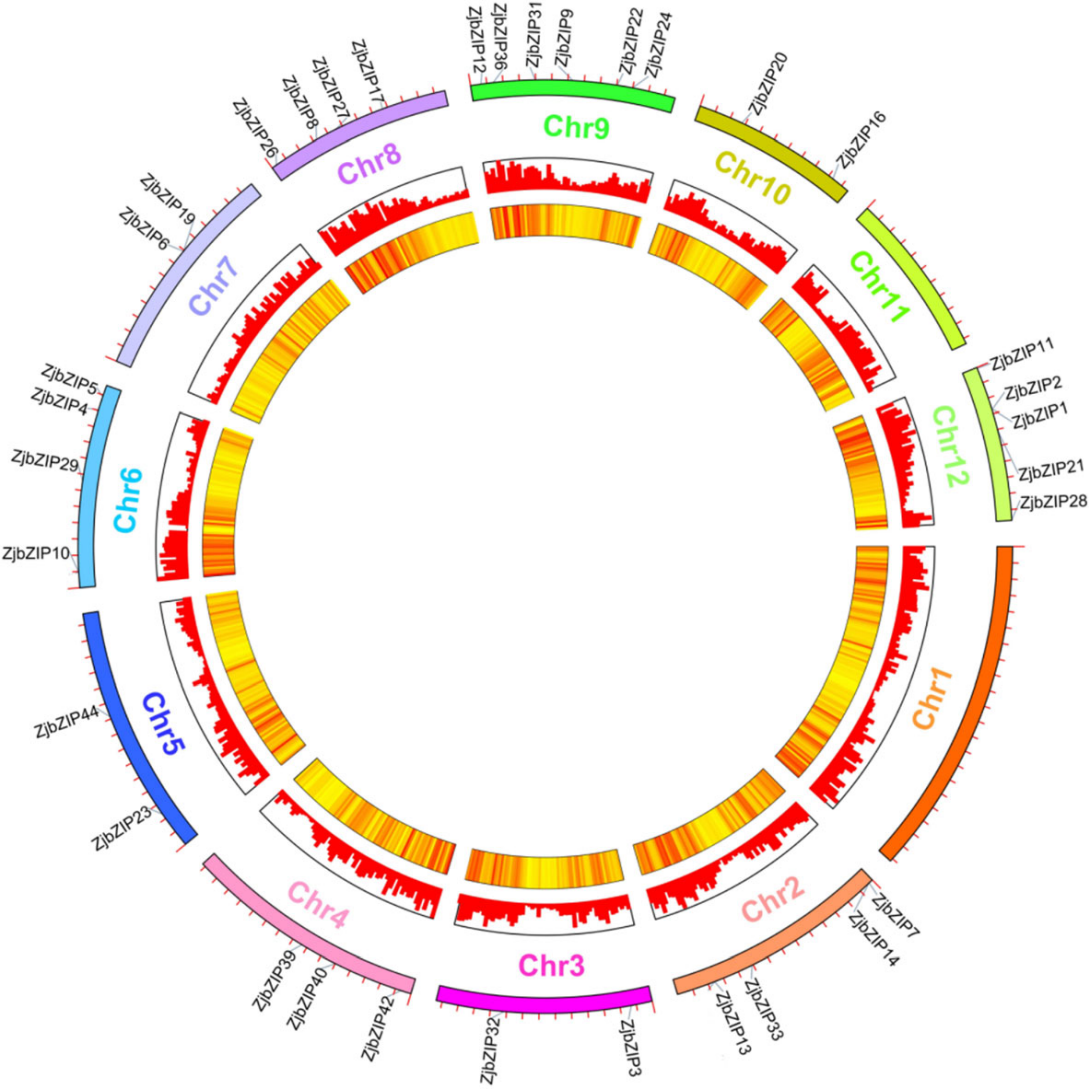
The chromosomal location of 34 *ZjbZIP*s*.* Genes are mapped to jujube chromosomes by the TBtools. The chromosomes of jujube are arranged in a circle

Of the 45 *ZjbZIP* genes, 34 were mapped to 10 pseudochromosomes in the jujube genome (Fig. 4), and 11 genes were unanchored. *ZjbZIPs* were not evenly distributed across the 12 chromosomes: there were 6 *ZjbZIPs* on both Chr. 9 and 12, and no genes were located on Chr. 1 or 11. Further analysis found that *ZjbZIP1* and *ZjbZIP2*, *ZjbZIP6* and *ZjbZIP19*, *ZjbZIP7* and *ZjbZIP14* are tandem repeat genes, indicating that some *ZjbZIPs* underwent gene duplication during jujube evolution to increase the number of genes and enhance their biological functions.

Additionally, the gene structure within the same subfamily was highly conserved (Additional file 3). We found that the genes in the C, D and G subfamilies contained more introns than did the genes in the other groups.

### Expression patterns of *ZjbZIP*s in various organs

**Fig. 5:**
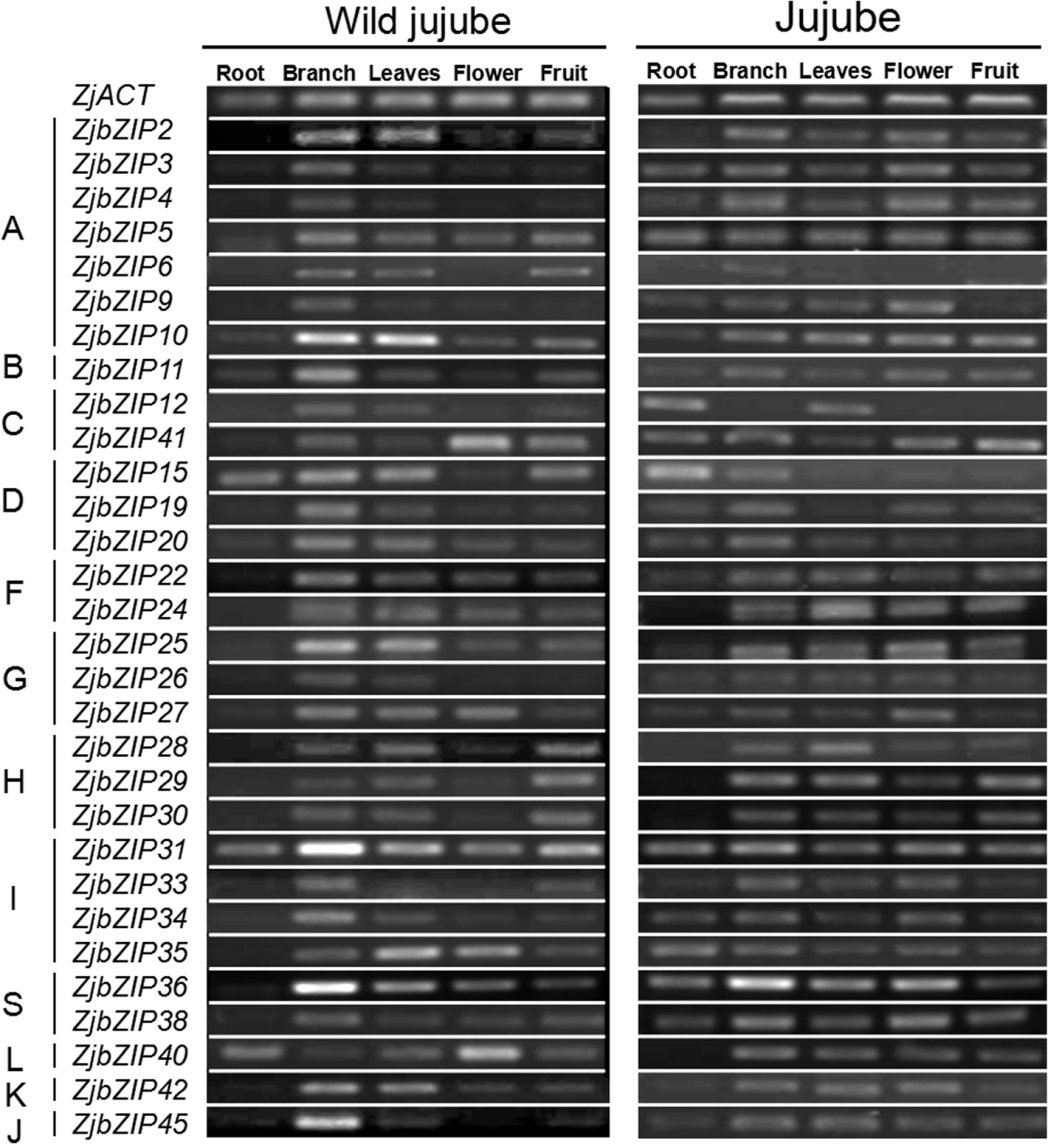
Expression patterns of 30 *ZjbZIP*s in five tissues of wild jujube and jujube by RT-PCR. *ZjACT* was used as an internal control. Left: wild jujube, from left to right: root, branch, leave, flower, and fruit. Right: jujube, from left to right: root, branch, leave, flower, fruit

To investigate the organ-specific expression of the *ZjbZIP* genes, the expression of 30 *ZjbZIP*s were analyzed in five organs of jujube and wild jujube by RT-PCR (Fig. 5, Additional file 4). It was shown that most *ZjbZIP*s were expressed in at least four organs, indicating that *ZjbZIPs* should involve in the development process of various organs in jujube and wild jujube. The expression of most *ZjbZIP* genes in the same subfamily showed similar patterns, suggesting that these genes in the same subfamily might have conserved functions.

In wild jujube, some *ZjbZIP* genes display a special expression pattern, in which *ZjbZIP10, − 25, − 31, − 36* were highly expressed in branch and leaf, indicating that these genes should play some roles in the development of the two organs. Compared with jujube, the expression levels of some genes, such as *ZjbZIP3, − 5, − 12, − 34, − 35, − 36, − 38*, and *− 41*, were significantly decreased in root of wild jujube, and only three genes, *ZjbZIP15, − 31, − 40,* showed lower expression (Fig. 5). These *ZjbZIP* genes may be related to their differing functions in root between wild jujube and jujube. We also found most of *ZjbZIPs* were expressed with varying degrees in fruit, and these genes should be candidate genes for further investigating in jujube fruit development. The broad and divergent expression patterns indicated that the *ZjbZIPs* should have multiple functions in jujube growth and development.

### *ZjbZIP*s involvement in jujube fruit development

**Fig. 6:**
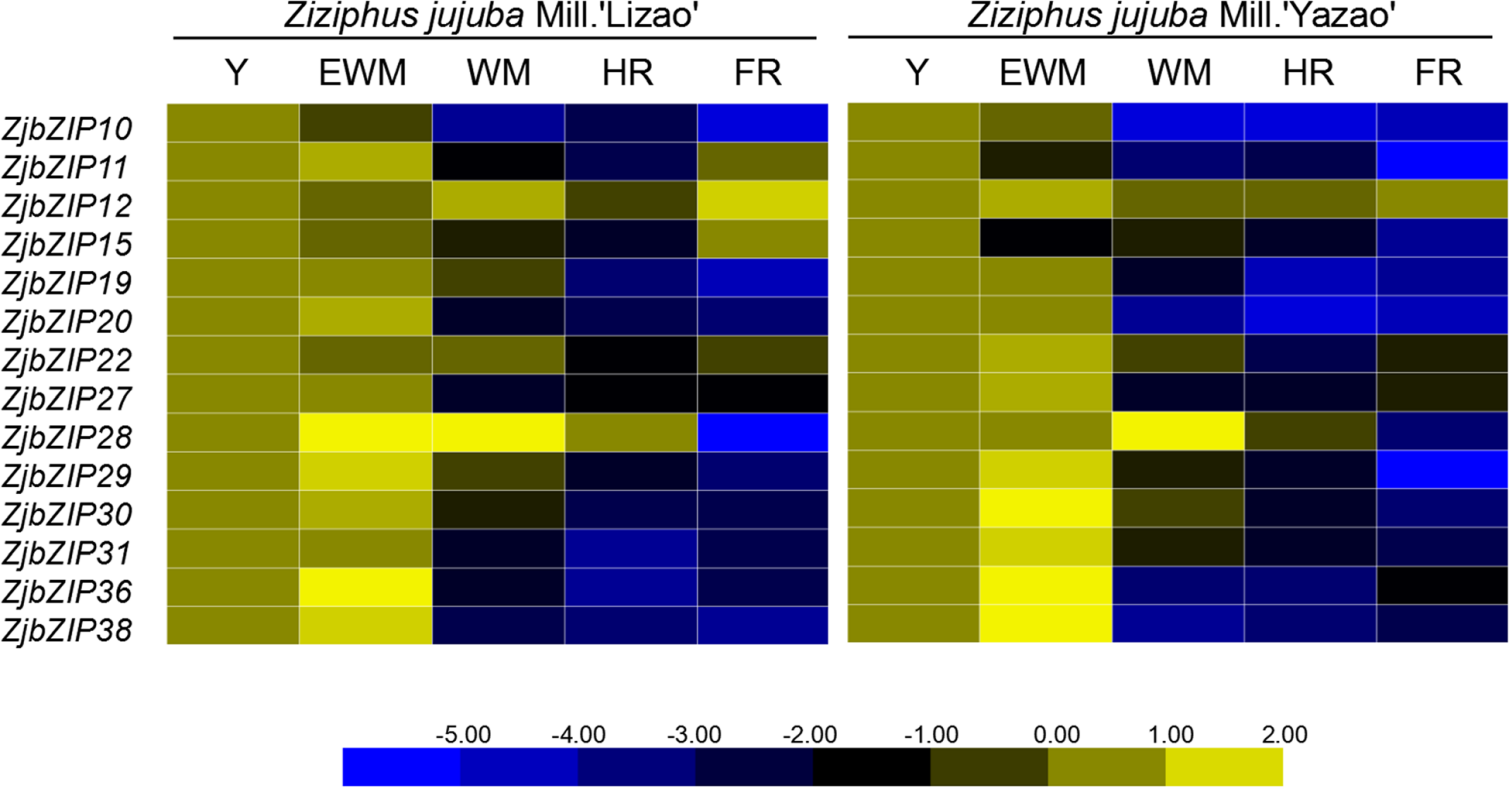
Heat maps of the relative expression of *ZjbZIP*s during fruit ripening in ‘Lizao’ and ‘Yazao’. Y, young fruit; EWM, early white mature fruit; WM, white mature fruit; HR, half-red fruit; FR, full red fruit. Scaled log2 expression values based on qRT-PCR data are shown from blue to yellow, indicating low to high expression

Based on organ-specific expression (Fig. 5), the highly expressed genes in fruit were further investigated at five development stages of jujube fruit. It is noteworthy that almost all of the *ZjbZIP*s tested were highly expressed at the first two stages and showed similar trends in both of the two cultivars (Fig. 6), indicating that these genes were positively involved in the fruit enlargement process. In other word, *ZjbZIP*s should play some significant roles in jujube fruit development.

Especially, *ZjbZIP28, − 29, − 30, − 36, − 38* showed the obvious increase in expression at the early white mature fruit stage (EWM), which period is just the fruit rapid expanding stage. These genes were identified as candidate genes for further study, in terms of their functions in fruit development.

### ZjbZIP protein-protein interaction network prediction

**Fig. 7:**
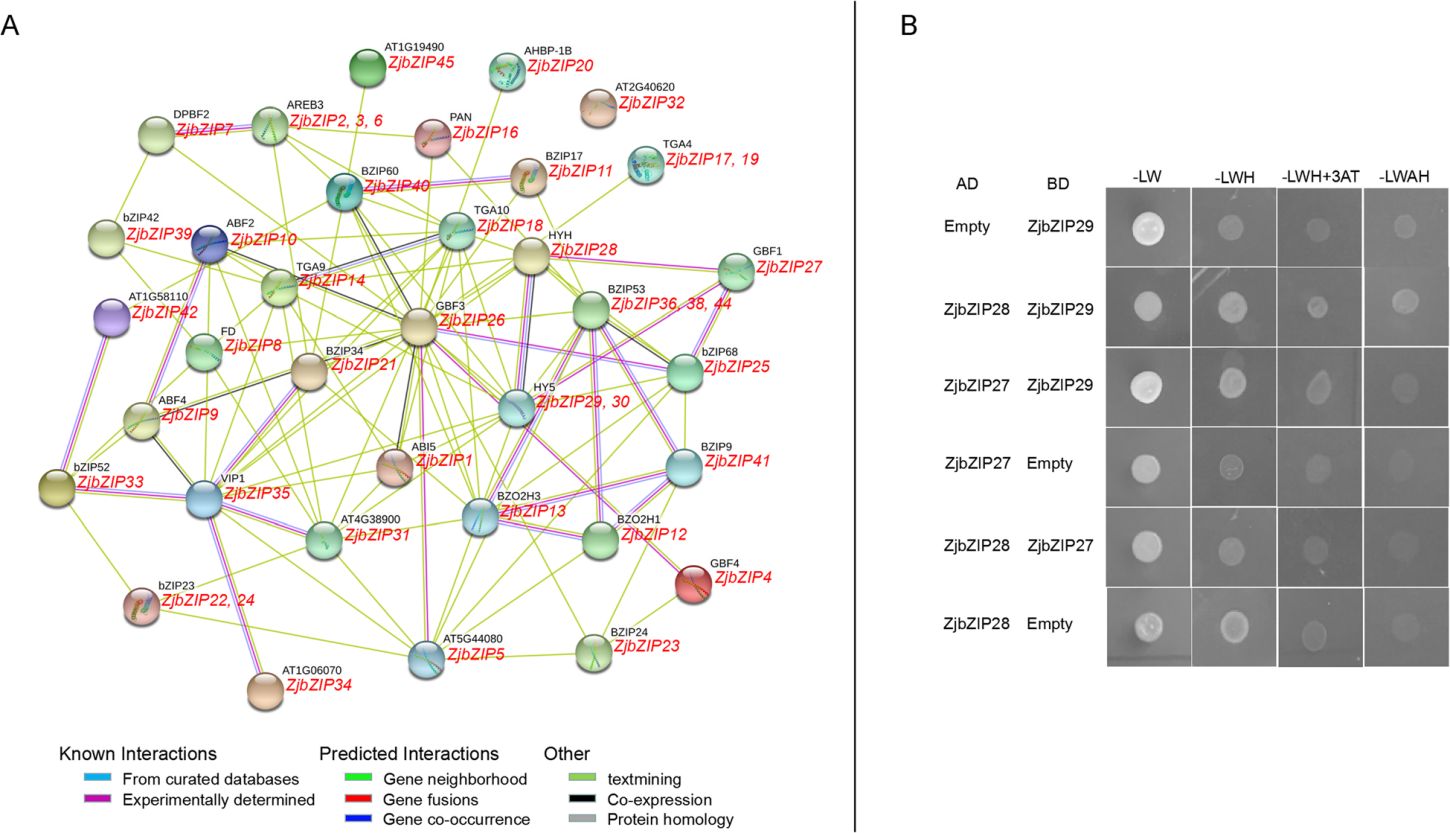
**a**: Protein-protein interaction network for ZjbZIPs based on their orthologs in Arabidopsis. This network was predicted by online software STRING. The ZjbZIP proteins were shown in the red font below with the Arabidopsis orthologs. **b**: Yeast two-hybrid screening of ZjbZIP27, ZjbZIP28 and ZjbZIP29

Based on their orthologs in Arabidopsis, ZjbZIPs were predicted to interact with each other by STRING (Fig. 7a), which was in accordance with previous reports that the binding activity of bZIPs depends upon the formation of homodimers or heterodimers among bZIPs [38]. Several interactions including ZjbZIP27 and ZjbZIP28, ZjbZIP29 and ZjbZIP28, were further confirmed by yeast two-hybrid in Fig. 7b (Additional file 4). As shown in Fig. 7a, both TGA9 (homolog of ZjbZIP14) and TGA10 (homolog of ZjbZIP18) are involved in the regulation of anther development [39], B202H1 (homolog of ZjbZIP12) can interact with BZIP53 (homolog of ZjbZIP36, ZjbZIP38 and ZjbZIP44) to promote their expression in seeds and are involved in defense responses [40], and the interaction between GBF4 (homolog of ZjbZIP4) and bZIP68 (homolog of ZjbZIP25) is regulated by light or other hormones [41].

### The expression of bZIP genes under abiotic and biotic stress

**Fig. 8:**
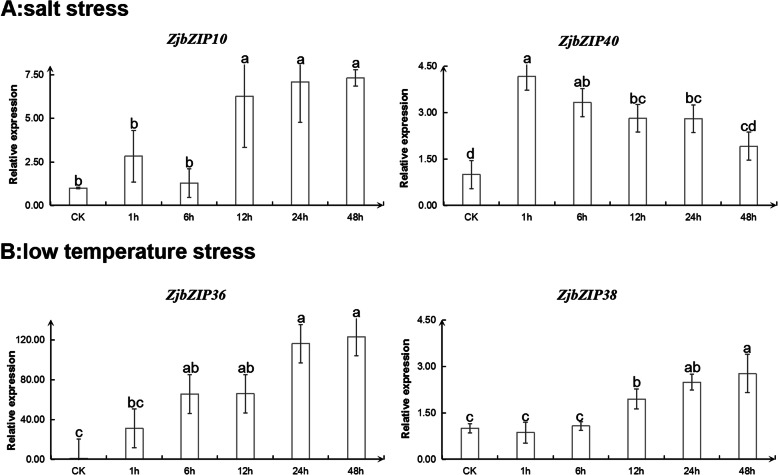
The relative expression of *ZjbZIPs* under abiotic stress. **a**: The relative expression of two *ZjbZIP*s under salt stress. **b**: The relative expression of two *ZjbZIPs* under low temperature stress. All statistical analyses were performed with SPSS software 17.0. Duncan’s multiple range tests were used to assess differences between treatments. Different letters mean significant difference at 0.05 levels between the corresponding treatments

According to protein-protein interaction predictions and previous studies, 11 *ZjbZIP*s were selected for further validation under abiotic stress, including low-temperature and salt stresses (Additional file 5). Under salt stress, the expression levels of *ZjbZIP10*, *11*, *23* and *40* (Additional file 5) increased with prolonged treatment time. The expression of *ZjbZIP10* increased significantly after 6 h of treatment and peaked at 48 h (Fig. 8a), and *ZjbZIP40* expression peaked at 1 h of treatment but then decreased. The expression of *ZjbZIP*3 showed no difference under salt stress. We also found that the expression of *ZjbZIP29, ZjbZIP36* and *38* increased markedly under low-temperature stress (Additional file 5 and Fig. 8b), and the expression of other three genes (*ZjbZIP17*, *22* and *24*) also increased in varying degrees (Additional file 5).

**Fig. 9:**
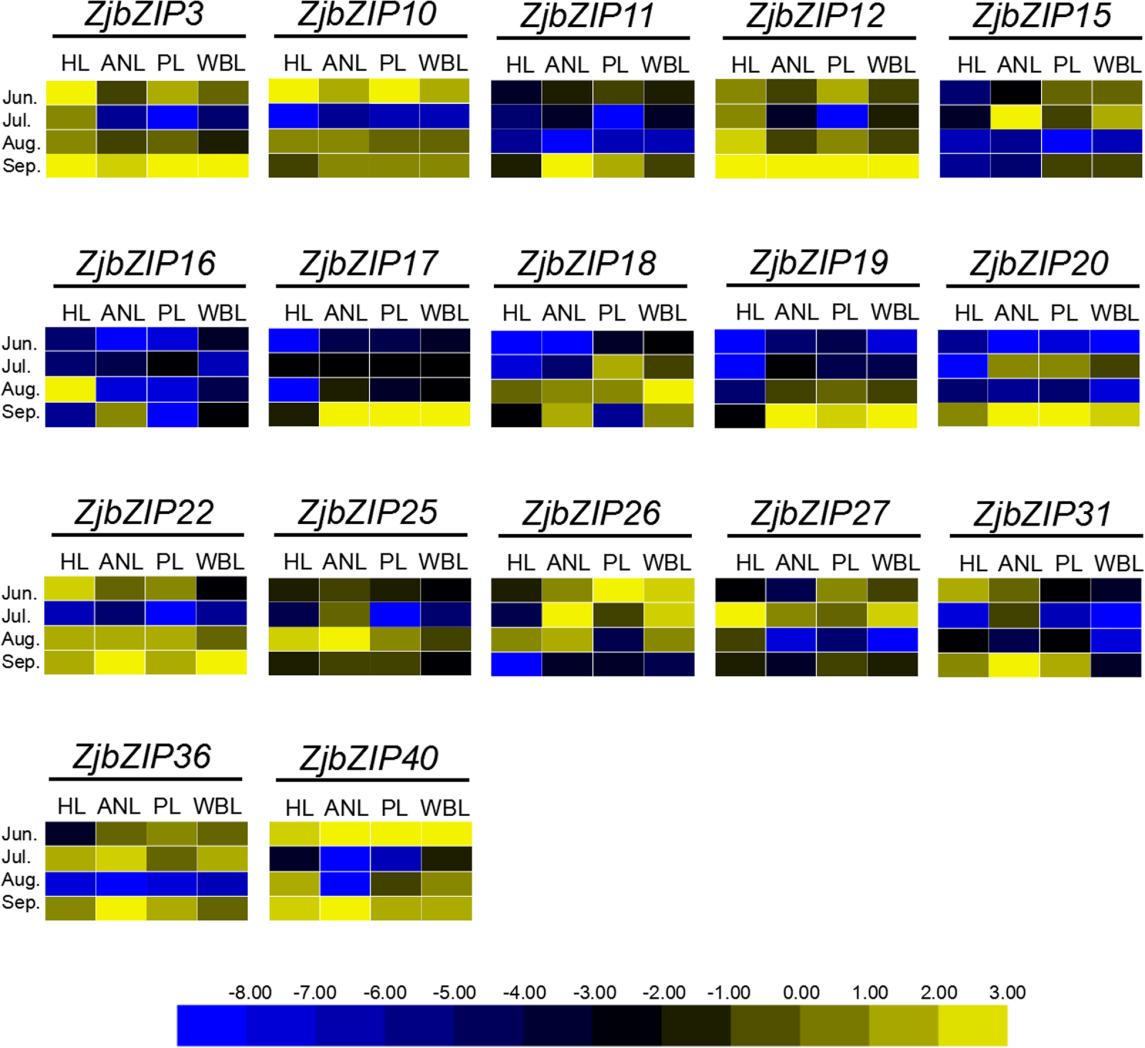
Heat maps of the relative expression of *ZjbZIP* genes under phytoplasma stress. Scaled log2 expression values based on qRT-PCR data are shown from blue to yellow, indicating low to high expression respectively

Jujube withes’ broom (JWB), which is caused by phytoplasma, is a destructive disease that affects jujube production. Since bZIP genes have multiple functions in plants, whether they participate in jujube-phytoplasma interactions remains unclear. Among the 17 *ZjbZIP*s identified (Fig. 9), the expression of *ZjbZIP3* and *12* in diseased leaves was significantly lower than that in healthy leaves. Moreover, the *ZjbZIP11*, *15*, *17*, *18*, *19*, *20* and *26* genes were highly expressed in diseased leaves. These results suggested that these *ZjbZIP*s participate in jujube-phytoplasma interactions and play different roles.

## Discussion

In this study, a total of 45 bZIP genes were identified in the jujube genome. A previous genome evolution study showed that Chinese jujube is closely related to species of the Rosaceae family [24], so the numbers of bZIPs from two Rosaceae species (apple and peach) [42], grape and Arabidopsis were compared with the number of ZjbZIPs. Compared with the number in some other plant species, the smaller number of bZIP genes in jujube and peach should be caused by the occurrence of only one whole genome duplication (WGD) event during the species evolution. Meanwhile, the divergence of gene number in same gene family of various species also relate to their evolutionary differences or the genome size [28].

In *Arabidopsis*, various subfamilies of *AtbZIP*s contain different conserved domains and perform various biological functions. For example, most members of the class A subfamily are involved in ABA pathway and abiotic stress responses, the class D members mainly play some roles in pathogen resistance and plant development, and the class S subfamily is involved in carbohydrate metabolism [34]. Here, the *ZjbZIP*s are highly homologous to sequences of the corresponding subfamilies in Arabidopsis, indicating that *ZjbZIP*s also might play different functions as a result of different conserved domains.

There are 10 TGA family members in Arabidopsis (Additional file 6), which are divided into five categories. A number of studies have shown that members of the TGA family play an important role in defense against biological and necrotizing pathogens [43, 44]. One member of the TGA family was found to interact with an ankyrin-repeat protein, a nonexpressor of the pathogen-associated (PR) gene (NPR1), which is a key component of the SA defense signaling pathway; SA is a key signaling molecule involved in plant resistance [45, 46]. Moreover, TGA members participate in mitotic reactions, regulate the growth and development of organisms, and play an important role in flower development [43]. Homology analysis and phylogenetic tree analysis indicated that subfamily D of *ZjbZIP* family is highly homologous to the TGA family in *Arabidopsis* (Additional file 6). It showed that ZjbZIP14 and TGA9 (At1g08320), ZjbZIP18 and TGA10 (At5g06839), ZjbZIP19 and TGA4 (At5g10030) were homologous (Additional file 6), suggesting that they might have similar biological functions and participate in pathogen defense reactions. Moreover, *ZjbZIP17*, − *18*, − *19* and − *20*, which are highly homologous to the TGA members in Arabidopsis, showed differential expression at the early stage of phytoplasma infection (Fig. 9). Phytoplasma causes one of serious disease in jujube production, named Jujube Witches’ Broom. Thus, these genes should involve in jujube-phytoplasma interaction.

*AtbZIP*s can recognize and bind to ABREs within promoters, which are named ABRE- binding factors (ABFs)/ABRE-binding proteins (AREBs) [9]. They play a key role in the regulation of the expression of downstream stress-responsive genes involved in ABA signaling. Genetic transformation of ABF/ABRE transcription factors has been suggested to be an effective approach for engineering stress-tolerant plants [47]. In this study, we also found that the expression level of *ZjbZIP10* was increased with time under salt stress. Thus, *ZjbZIP10* may have the function in terms of involvement in the resistance to salt stress. In addition, the expression of *ZjbZIP36* was significantly induced under low-temperature stress, indicating that it might be involved in the response to cold stress.

Overall, all the results imply that *ZjbZIPs* play multiple roles in jujube development and under biotic and abiotic stresses. Further function verifications are worthwhile and need to reveal their regulation metabolism in detail.

## Conclusions

The bioinformatics analyses of 45 ZjbZIPs were firstly investigated in this study. Meanwhile, their expression patterns in jujube and wild jujube were measured by qPCR during fruit ripening and in the response to phytoplasma and abiotic stresses, and some candidate genes involved in these processes were screened out. The protein interactions among ZjbZIPs were predicted, which provide useful information for further functional studies to elucidate their regulation mechanism.

## Methods

### Plant materials

The Chinese jujube and wild jujube trees used in this study were cultivated at the Experimental Station of Chinese Jujube, Hebei Agricultural University, Baoding, China. They are very common fruit trees in China and are not endangered species. No specific permits were required for the sample collection.

Five organs (roots, branches, leaves, flowers and fruits) collected from three jujube trees and three wild jujube trees were used for organ-specific expression analysis. In general, jujube fruit development can be divided into five typical stages: the young fruit stage (Y), early white mature fruit stage (EWM), white mature fruit stage (WM), half-red fruit stage (HR) and full-red fruit stage (FR). The first two stages of jujube fruit development (young fruit stage (Y) and early white mature fruit stage (EWM)) are crucial to fruit enlargement, and the later three stages are important to fruit maturity. *Z. jujuba* Mill. ‘Lizao’ and ‘Yazao’ are two jujube fresh cultivars, which fruit display five typical developmental stages and suitable to study the fruit development. Thus, fruit samples of the two cultivars were taken at the five developmental stages and used to investigate the expression patterns of *ZjbZIP*s. Each treatment consisted of three biological replicates.

Three healthy trees and three JWB diseased trees of *Z. jujuba* Mill. ‘Dongzao’ were used to collect the leave samples at four stages (June, July, August and September). There are four kinds of leaves, i.e., healthy leaves (HL), apparently normal leaves (ANL), phyllody leaves (PL), and witches’ broom leaves (WBL). All treatments consisted of three biological replicates. All fresh samples were frozen in liquid nitrogen immediately, and then stored at − 80 °C for RNA extraction.

Cold- and salt-stress treatments were performed on the callus tissues of *Z. jujuba* Mill. ‘Guanyangchangzao’. For the cold treatment, calli at same growth stage and condition were transferred at 4 °C and then collected within 0, 1, 6, 12, 24, and 48 h. Calli incubated at 25 °C were collected and used as negative controls [26]. For the salinity treatment, callus tissues were subjected to a 150 mM NaCl and NaHCO_3_-NaOH solution (pH 9.5) for 0, 1, 6, 12, 24, and 48 h. Samples subjected to sterile water treatment rather than the NaCl and NaHCO_3_-NaOH solution were used as negative controls [48].

### Identification and protein structure analysis of ZjbZIPs

A hidden Markov model (HMM) file of the bZIP domain (PF00170) was downloaded (Pfam 31.0, https://pfam.xfam.org/) and used as the template to identify *ZjbZIP* sequences in the jujube genome [24]. Above sequences were further confirmed by using the Plant Transcription Factor Database (PlantTFDB, http://planttfdb.cbi.pku.edu.cn/) and the online CD search tool in NCBI database (https://www.ncbi.nlm.nih.gov/). Some characters of the *ZjbZIP* genes were also predicted by the bioinformatical tools [49]. GC content and RSCU were performed by software codonW1.4.4 (http://codonw.sourceforge.net/). The conserved motifs of ZjbZIP proteins were detected by MEME (http://meme-suite.org/).

### The chromosomal location and gene structure of ZjbZIPs

The chromosomal locations of the *ZjbZIP* genes and their tandem duplications were analyzed as previously described [49, 50]. TBtools (http://cj-chen.github.io/tbtools/) was used to draw a circle graph. The gene structure of *ZjbZIPs* was predicted by website GSDS (http://gsds.cbi.pku.edu.cn/) [49, 51].

### Multiple sequence alignment and phylogenetic tree construction

The multiple sequence alignment results were analyzed by using DNAMAN 8.0 software. A phylogenetic tree consisting of 45 ZjbZIPs was constructed based on their conserved domains. bZIP proteins from *Arabidopsis thaliana* and *Malus* were downloaded from the NCBI (https://www.ncbi.nlm.nih.gov/) and PlantTFDB (http://planttfdb.cbi.pku.edu.cn/) (Additional file 7) and used to construct the phylogenetic tree [52, 53]. In addition, the phylogenetic trees and line charts for a lineage of gene groups for *ZjbZIP26* and *29* were analyzed in the Gcorn plant database (http://www.plant.osakafu-u.ac.jp/~kagiana/gcorn/p/) [37].

### RNA isolation and expression analysis

The procedures of total RNA isolation, detection and cDNA synthesis were performed according to previous methods [49]. Both semiquantitative PCR and qRT-PCR were used to measure the expression of *ZjbZIPs*. The primer sequences were listed in Additional file 8. The PCR reaction system and procedures in the study were implemented as the method described [49, 54, 55]. Three biological replicates were analyzed for each treatment.

All statistical analyses were performed with SPSS software 17.0. Duncan’s multiple range tests were used to assess differences among different treatment times. Different letters mean significant difference at 0.05 levels between the corresponding treatments.

### Protein-protein interaction predictions

45 ZjbZIP protein sequences were used as the targets and their orthologs of *Arabidopsis thaliana* were appointed as references, and the STRING website (https://string-db.org/) was employed to predict protein-protein interactions [49]. Yeast two-hybrid screening (Y2H) [56] was further applied to verify several interactions.

## Supplementary information

**Additional file 1: Fig. S1.** The phylogenetic analysis of bZIP proteins of *Ziziphus jujuba* Mill and *Malus domestica.* The NJ tree was constructed from the bZIP protein sequences using MEGA7 with 1000 bootstrap copies.

**Additional file 2: Fig. S2.** The conserved motifs of ZjbZIP proteins.

**Additional file 3: Fig. S3.** The gene structure of 45 *ZjbZIPs* in Chinese jujube. Introns and exons are represented by black lines and red boxes respectively and upstream/downstream are represented by green boxes.

**Additional file 4.** A: All original and full-length gels in Fig. 5; B: All original and blot images in Fig. 7b.

**Additional file 5: Fig. S4.** The relative expression of *ZjbZIP*s under salt stress and low temperature stress.

**Additional file 6: Fig. S5.** A: The phylogenetic analysis of bZIP proteins of *Ziziphus jujuba* and *Arabidopsis thaliana.* The NJ tree was constructed from the protein sequences of ZjbZIPs and AtbZIPs using MEGA7 with 1000 bootstrap copies. B: The protein-protein interaction analysis of two ZjbZIPs by STRING database.

**Additional file 7: Table S1.** The bZIP transcription factors in *Arabidopsis* and apple used in this study. (XLS 38 kb)

**Additional file 8: Table S2.** The primers of *ZjbZIPs* used in this study. (XLS 29 kb)

## Data Availability

All data and materials used in this study are publicly available. All accession numbers deposited in the NCBI and PlantTFDB have been listed in Table 1 and additional file 7. The other accession numbers (XP_004138731, NP_001284656, XP_006450470, XP_006483336, At1g08320, At5g06839 and At5g10030) and related web links are included in the manuscript.

## Abbreviations

TFs: transcription factors
bZIP: basic helix-loop-helix
TGA: TGACG motif-binding factor
HIs: high homology indices
qRT-PCR: quantitative real-time PCR
HMM: hidden Markov model
JWB: jujube witches’ broom disease
ANL: apparently normal leaves
PL: phyllody leaves
WBL: witches’ broom leaves
HL: healthy leaves
Y: young fruit stage
EWM: early white mature fruit stage
WM: white mature fruit stage
HR: half-red fruit stage
FR: full-red fruit stage
