# Supplementary figures and images for: Genome-wide analysis of the bZIP gene family in Chinese jujube (Ziziphus jujuba Mill.)
Source: BMC Genomics. 2020 Jul 14;21:483. doi: 10.1186/s12864-020-06890-7 (PMC7362662; doi:10.1186/s12864-020-06890-7)

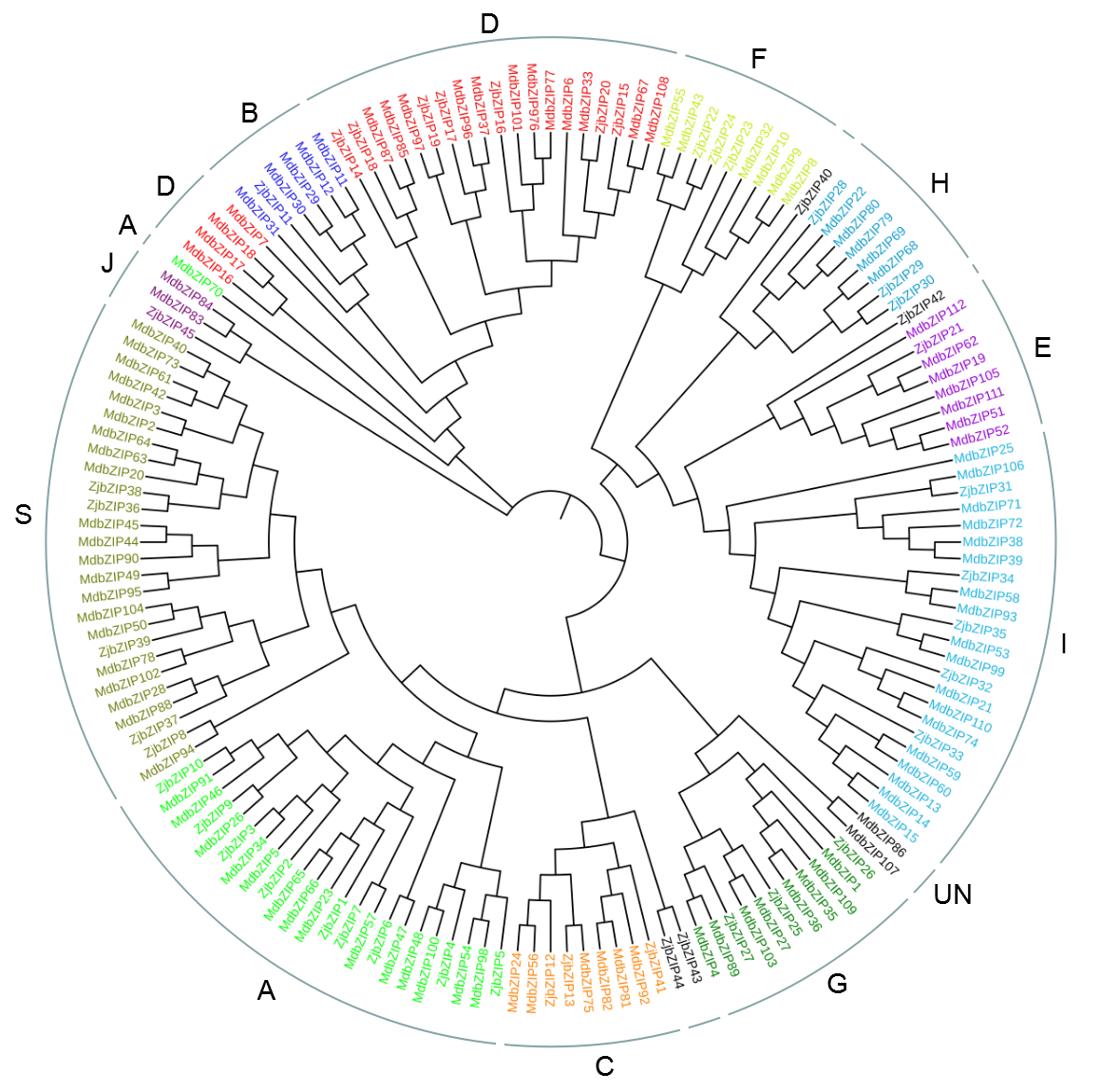

Supplement: Supplementary file 1 — Additional file 1: Fig. S1. The phylogenetic analysis of bZIP proteins of Ziziphus jujuba Mill and Malus domestica. The NJ tree was constructed from the bZIP protein sequences using MEGA7 with 1000 bootstrap copies. [file 12864_2020_6890_MOESM1_ESM.png]

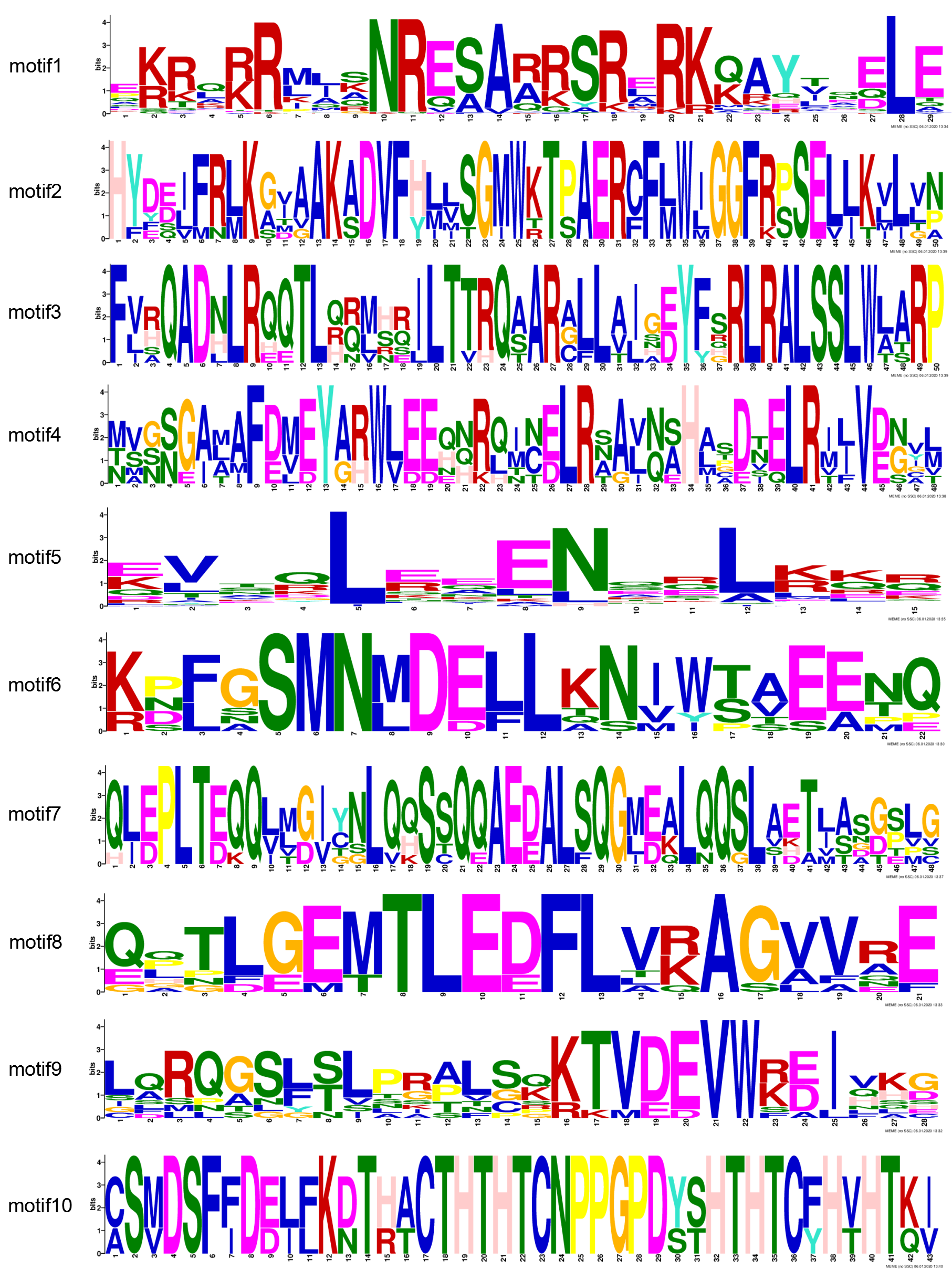

Supplement: Supplementary file 2 — Additional file 2: Fig. S2. The conserved motifs of ZjbZIP proteins. [file 12864_2020_6890_MOESM2_ESM.tif]

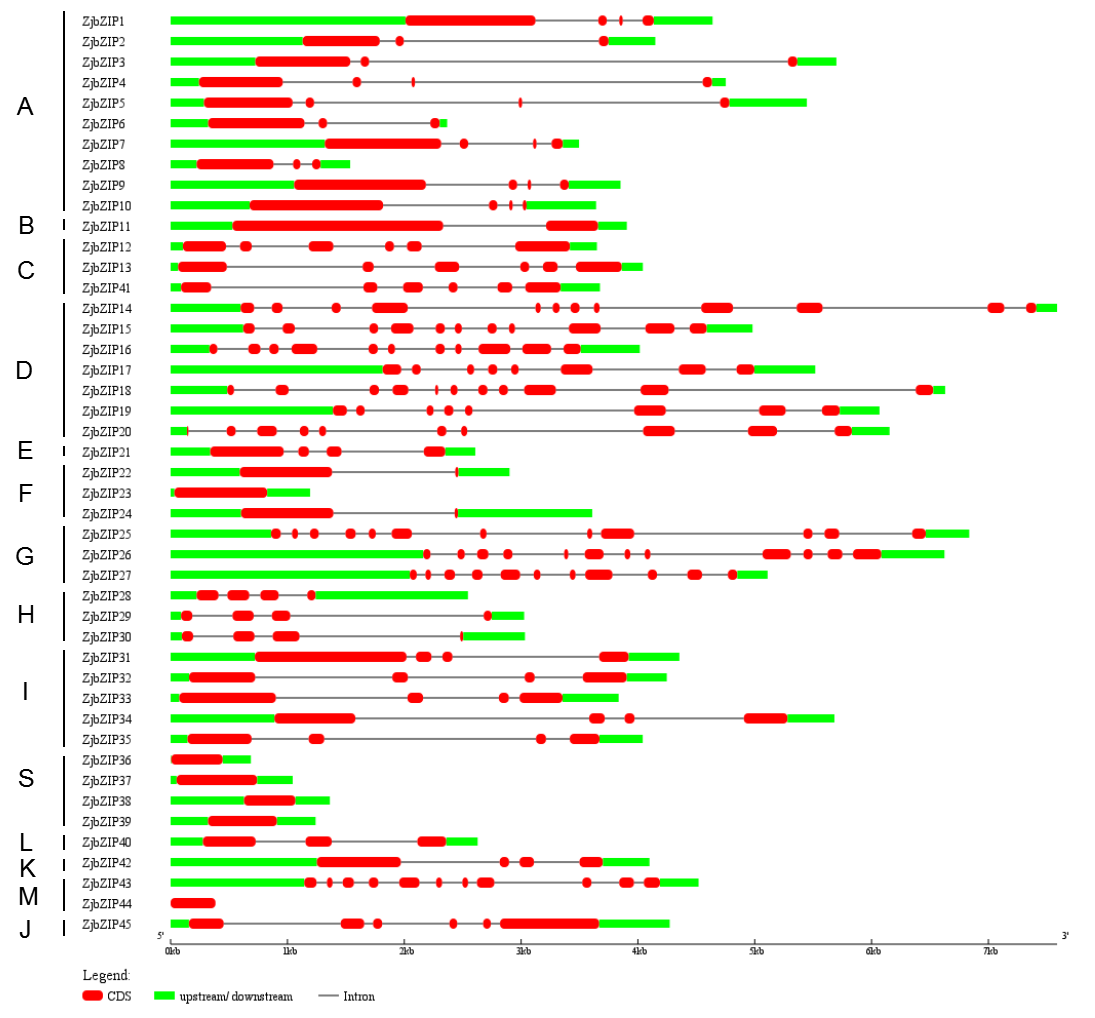

Supplement: Supplementary file 3 — Additional file 3: Fig. S3. The gene structure of 45 ZjbZIPs in Chinese jujube. Introns and exons are represented by black lines and red boxes respectively and upstream/downstream are represented by green boxes. [file 12864_2020_6890_MOESM3_ESM.png]

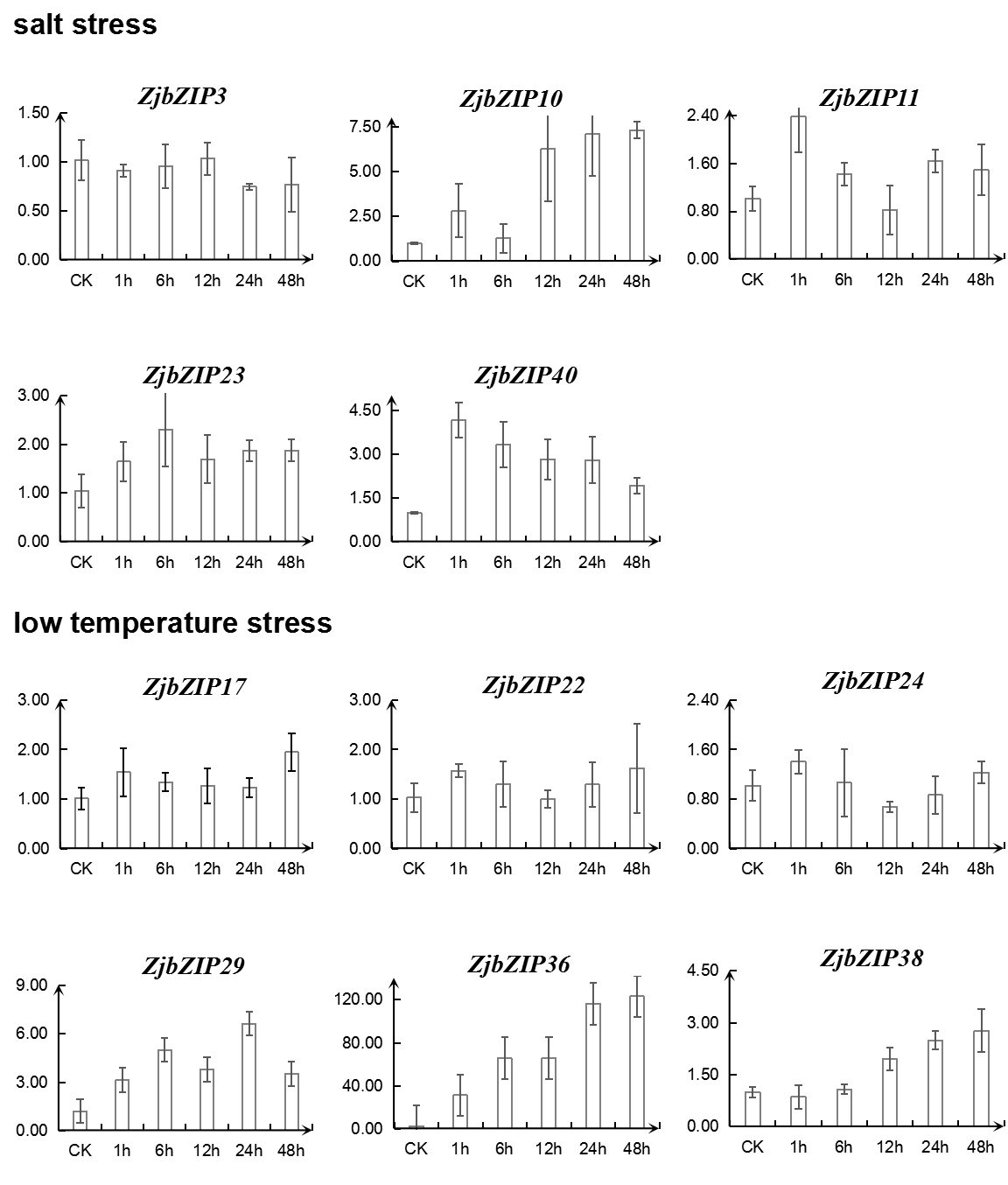

Supplement: Supplementary file 5 — Additional file 5: Fig. S4. The relative expression of ZjbZIPs under salt stress and low temperature stress. [file 12864_2020_6890_MOESM5_ESM.tif]

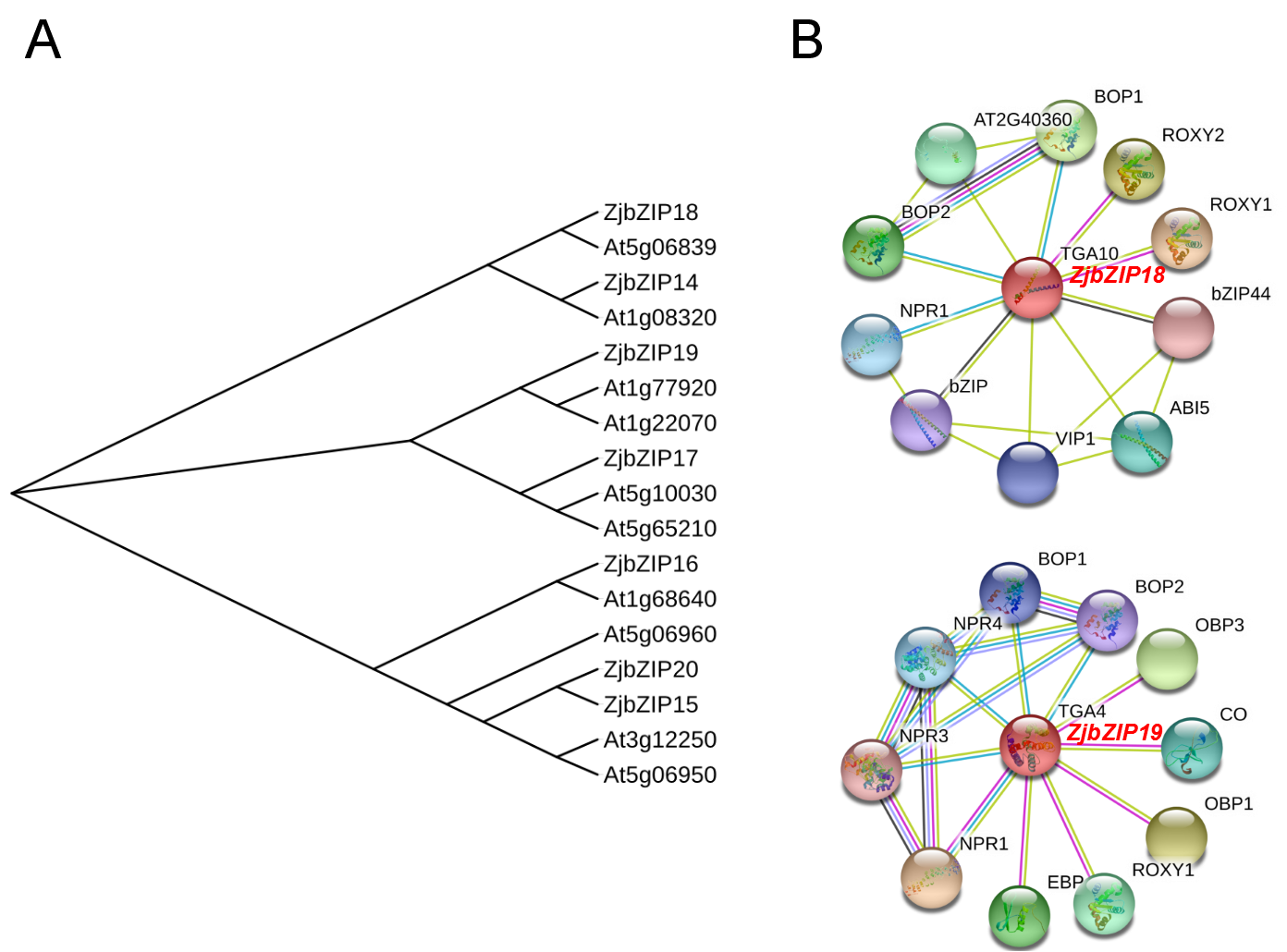

Supplement: Supplementary file 6 — Additional file 6: Fig. S5. A: The phylogenetic analysis of bZIP proteins of Ziziphus jujuba and Arabidopsis thaliana. The NJ tree was constructed from the protein sequences of ZjbZIPs and AtbZIPs using MEGA7 with 1000 bootstrap copies. B: The protein-protein interaction analysis of two ZjbZIPs by STRING database. [file 12864_2020_6890_MOESM6_ESM.tif]
