# Supplementary material for: Genome-wide analysis of the bZIP gene family in Chinese jujube (Ziziphus jujuba Mill.)
Source: BMC Genomics. 2020 Jul 14;21:483. doi: 10.1186/s12864-020-06890-7 (PMC7362662; doi:10.1186/s12864-020-06890-7)
Supplement: Supplementary file 4 — Additional file 4. A: All original and full-length gels in Fig. 5; B: All original and blot images in Fig. 7b. [file 12864_2020_6890_MOESM4_ESM.doc]

**Additional file 5**

A. All original, full-length gels in Fig. 5 were now as the following.

**Expression patterns of the genes in five tissues of wild jujube and jujube by RT-PCR.**


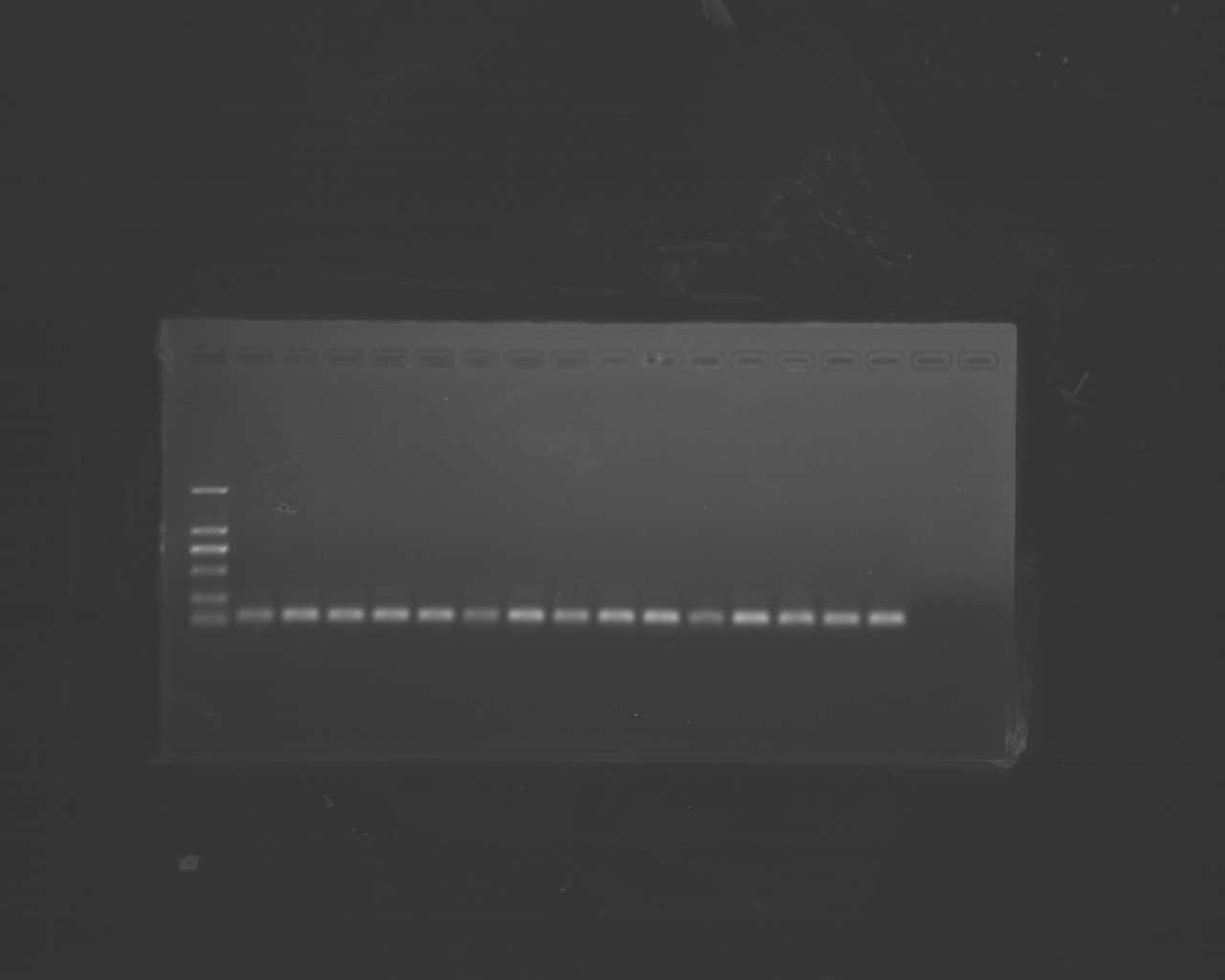

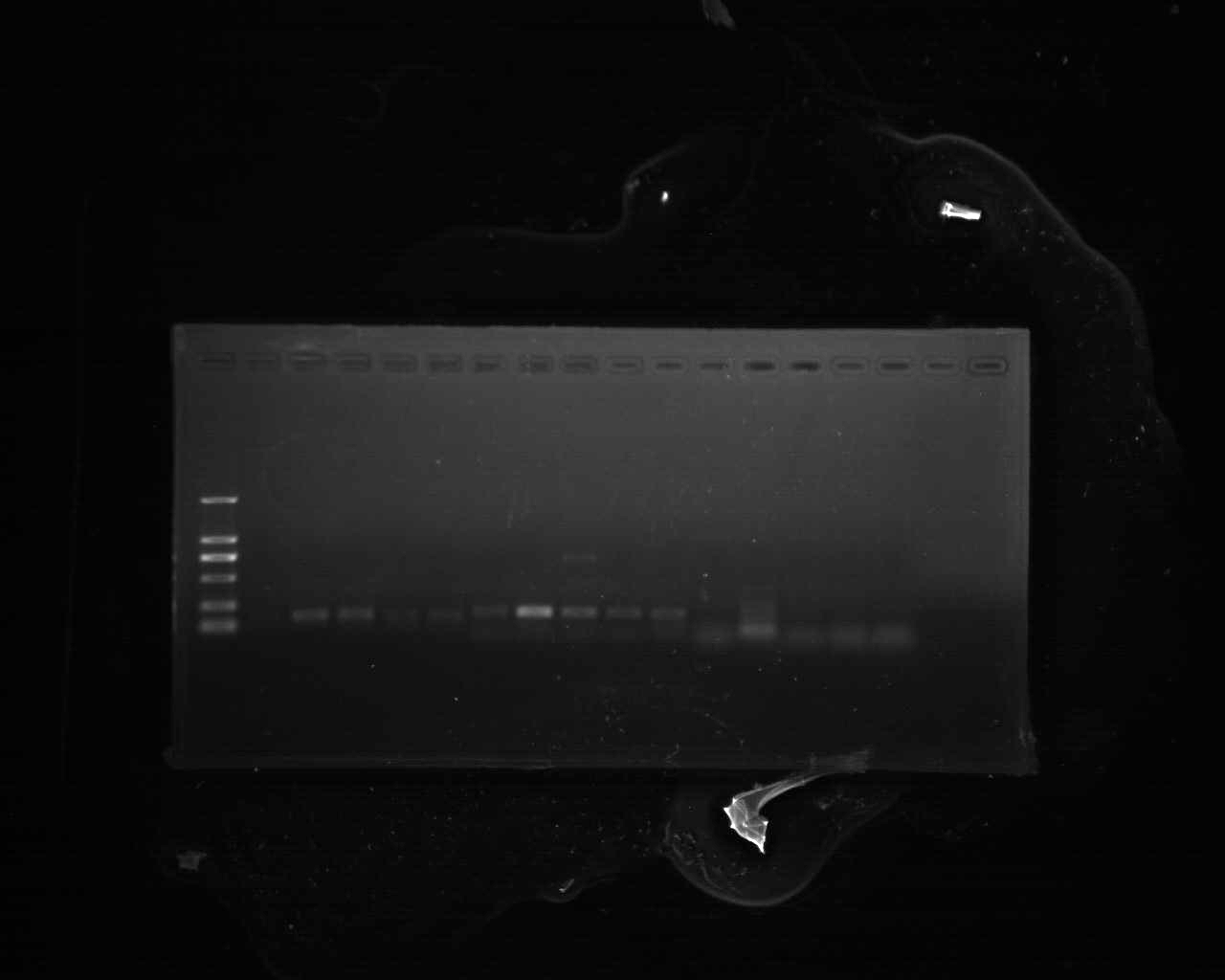

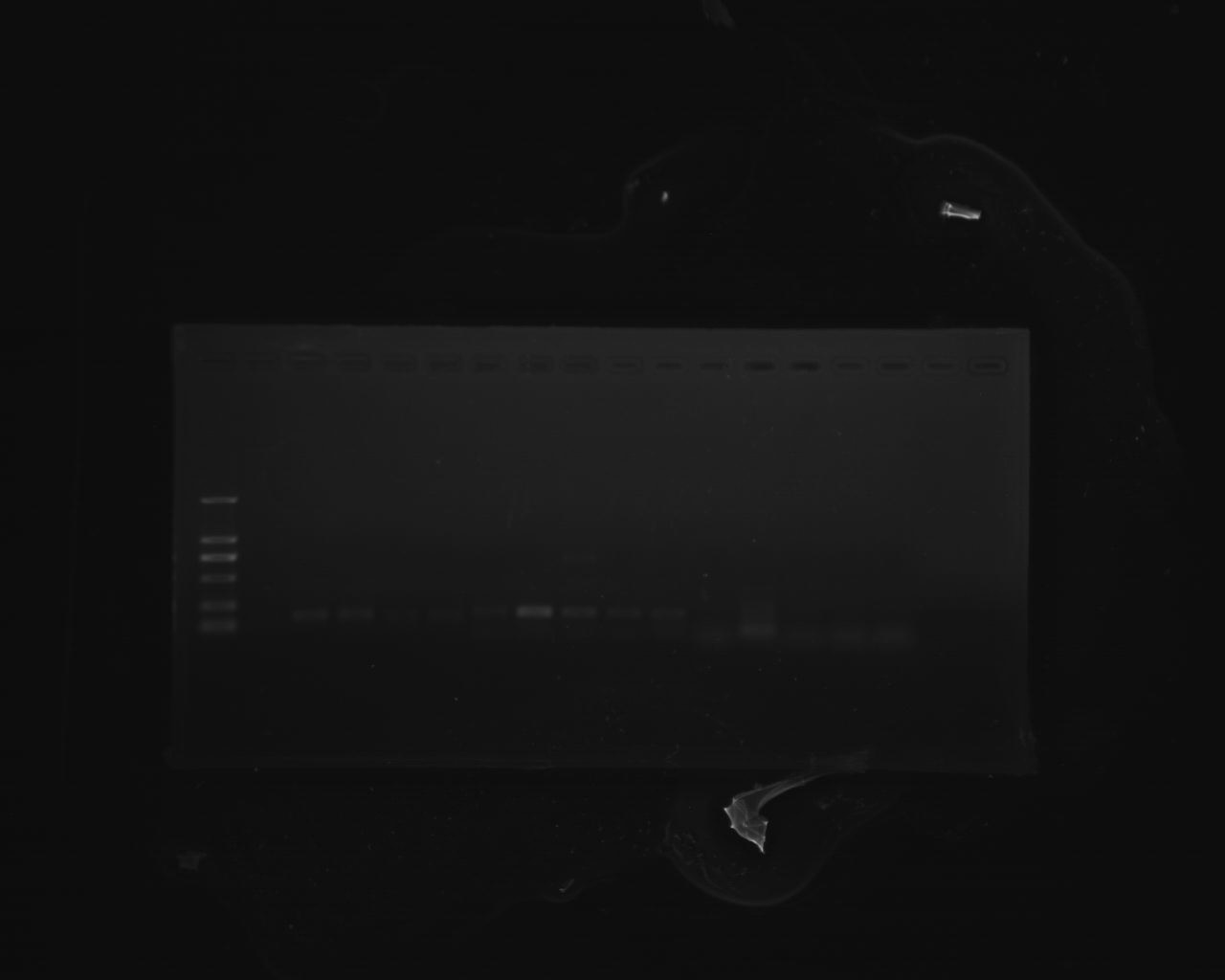


Root Branch Leaves Flower Fruit

***ZjACT***

Root Branch Leaves Flower Fruit

Root Branch Leaves Flower Fruit

*ZjbZIP2*

*ZjbZIP3*


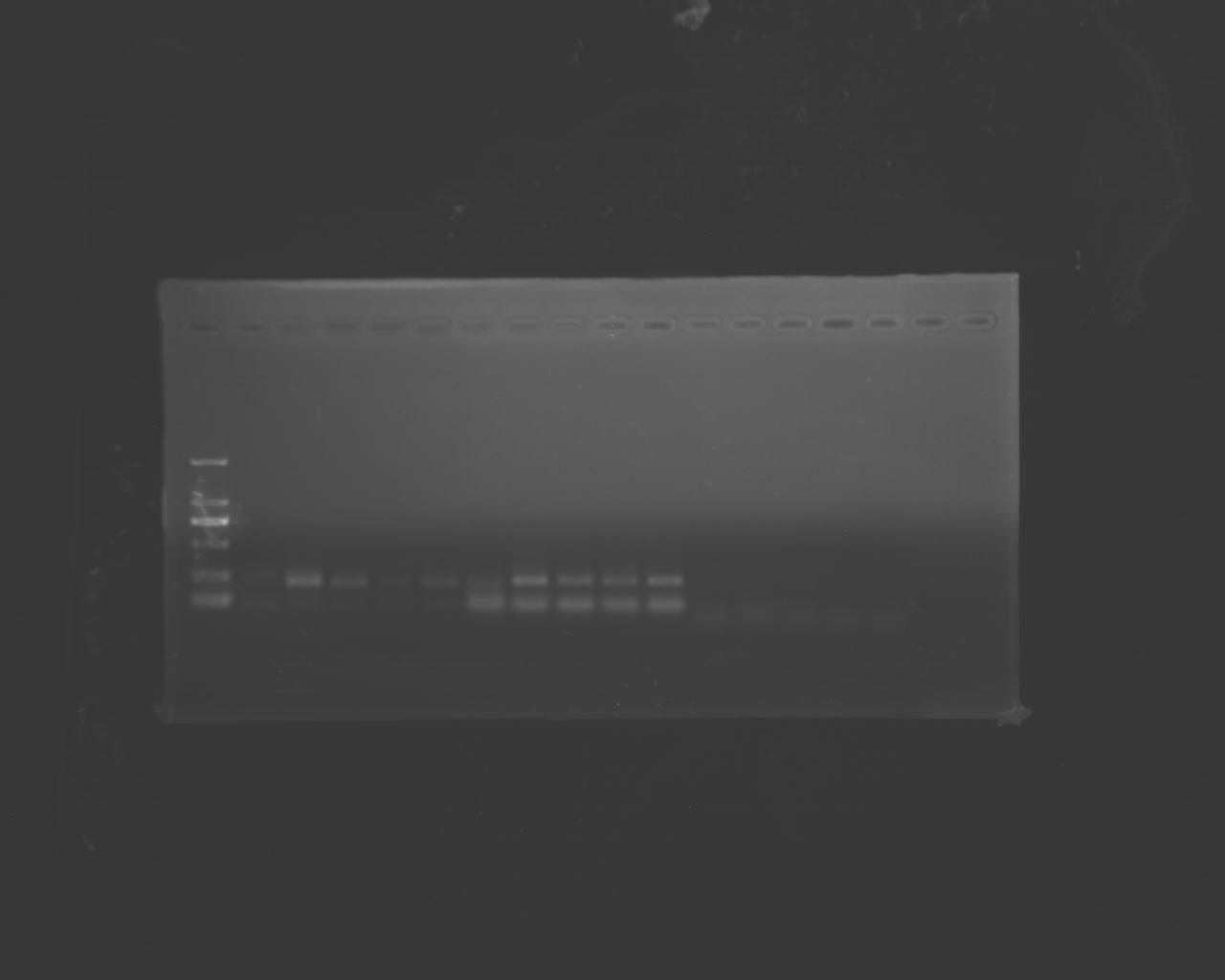


*ZjbZIP4*

Root Branch Leaves Flower Fruit


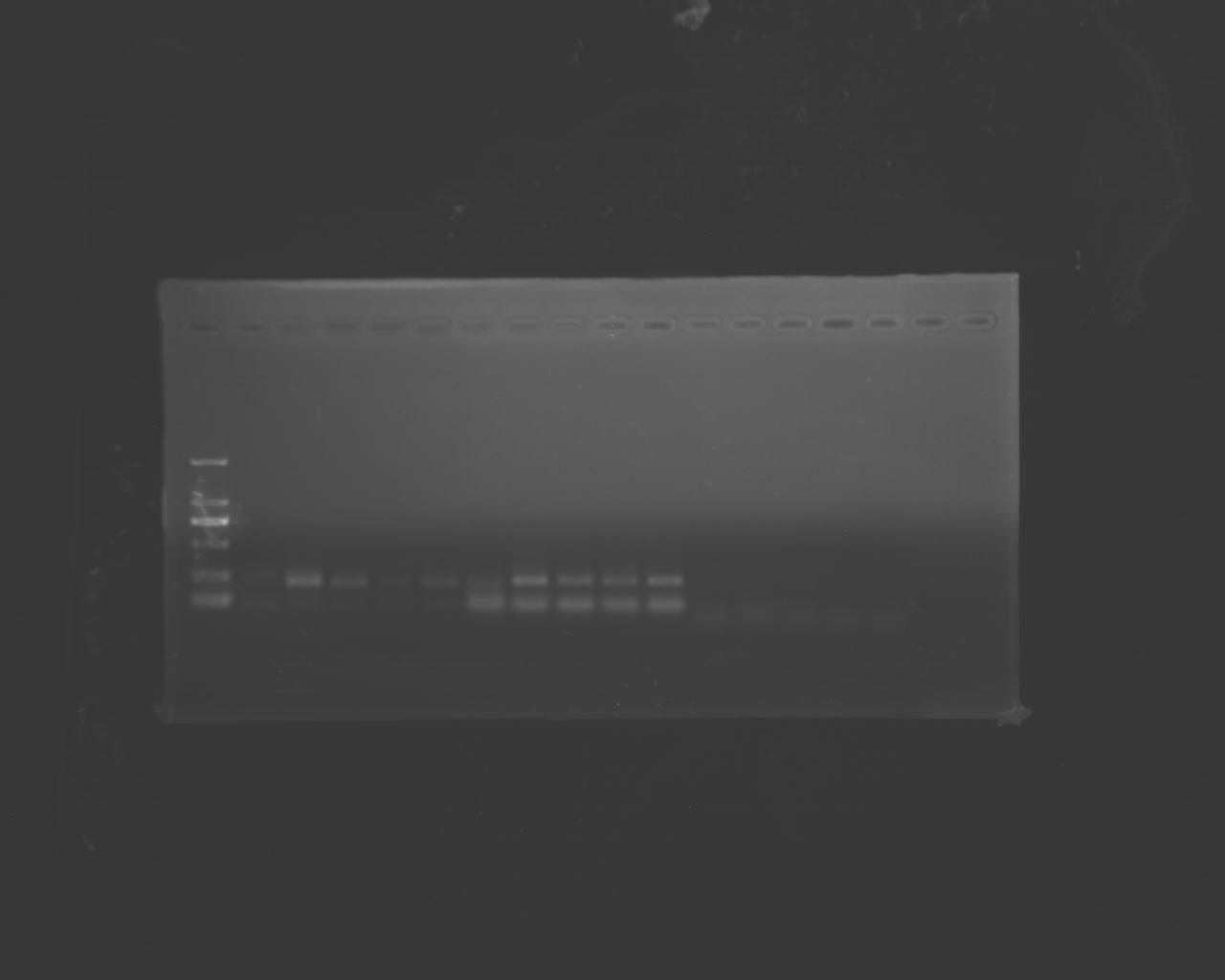


***ZjbZIP5***

Root Branch Leaves Flower Fruit


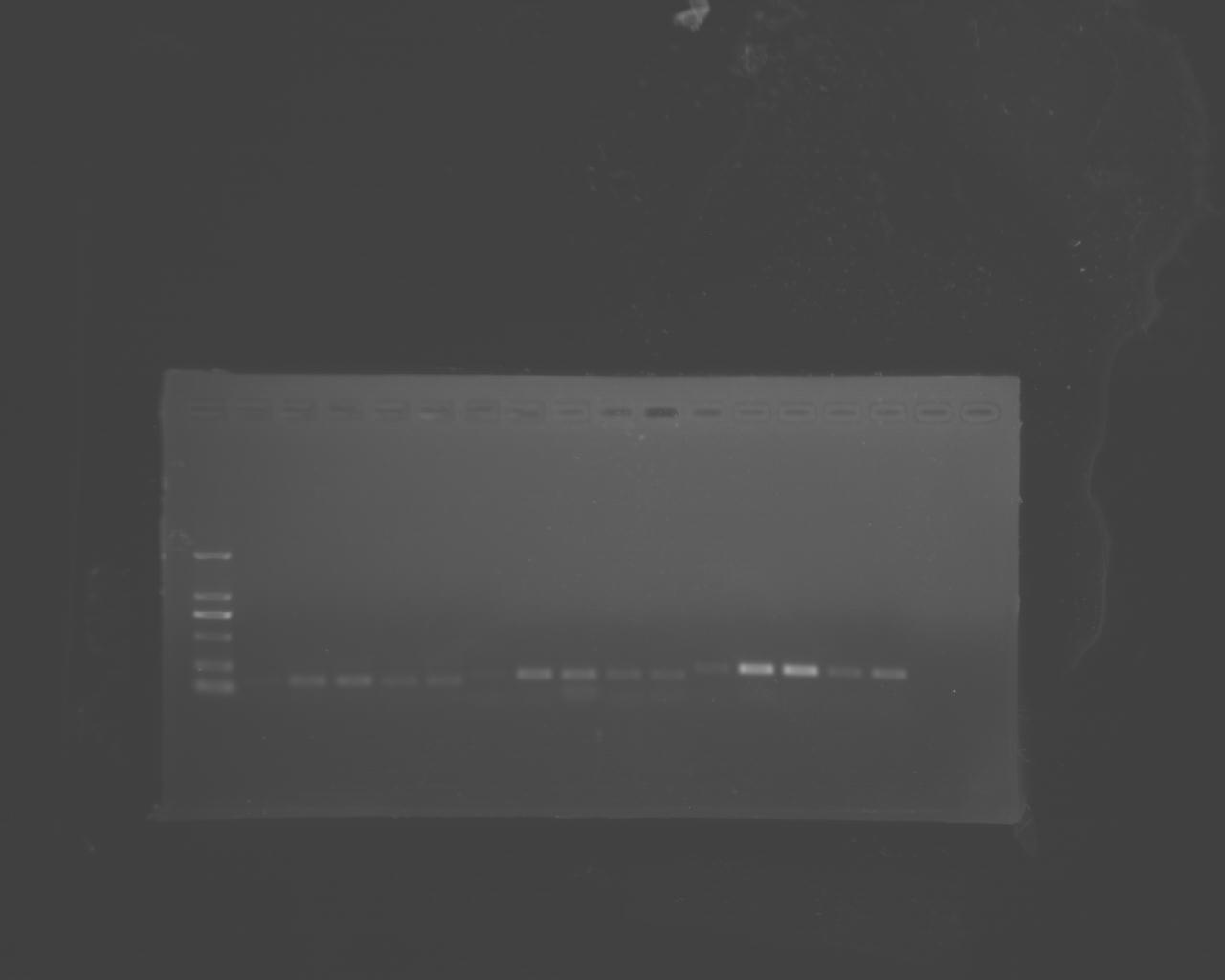


ZjbZIP6

Root Branch Leaves Flower Fruit


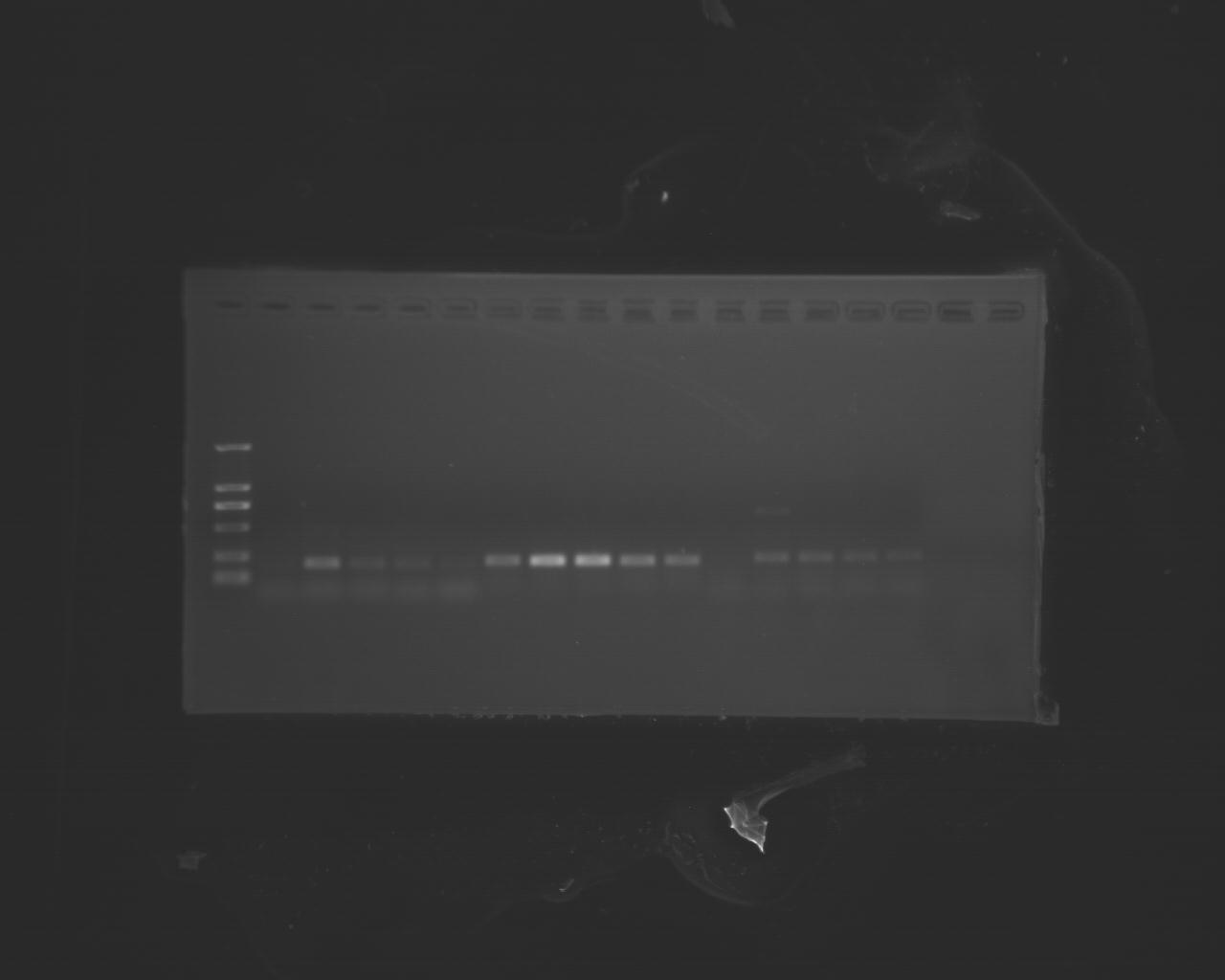


ZjbZIP9

Root Branch Leaves Flower Fruit


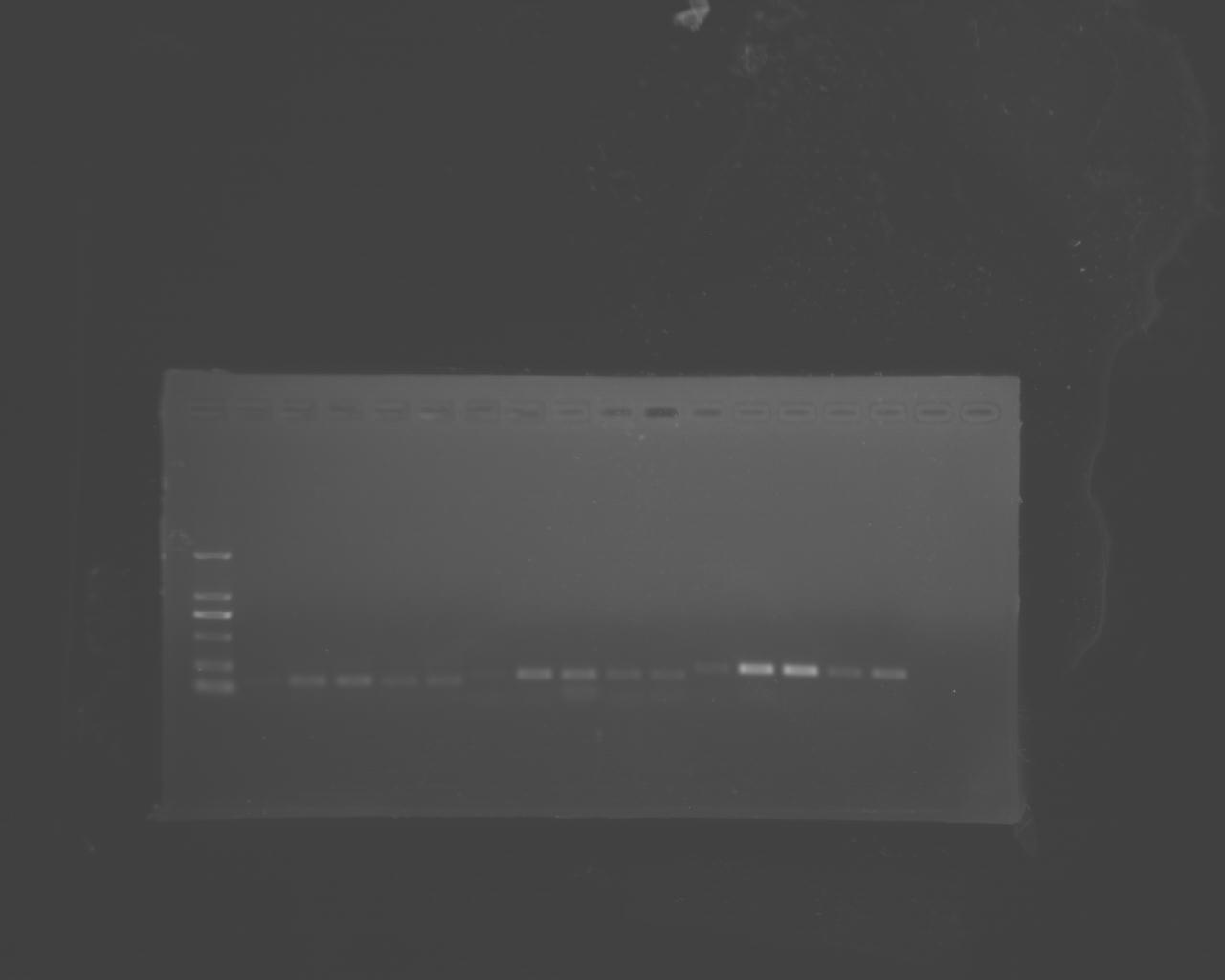


Root Branch Leaves Flower Fruit

ZjbZIP10


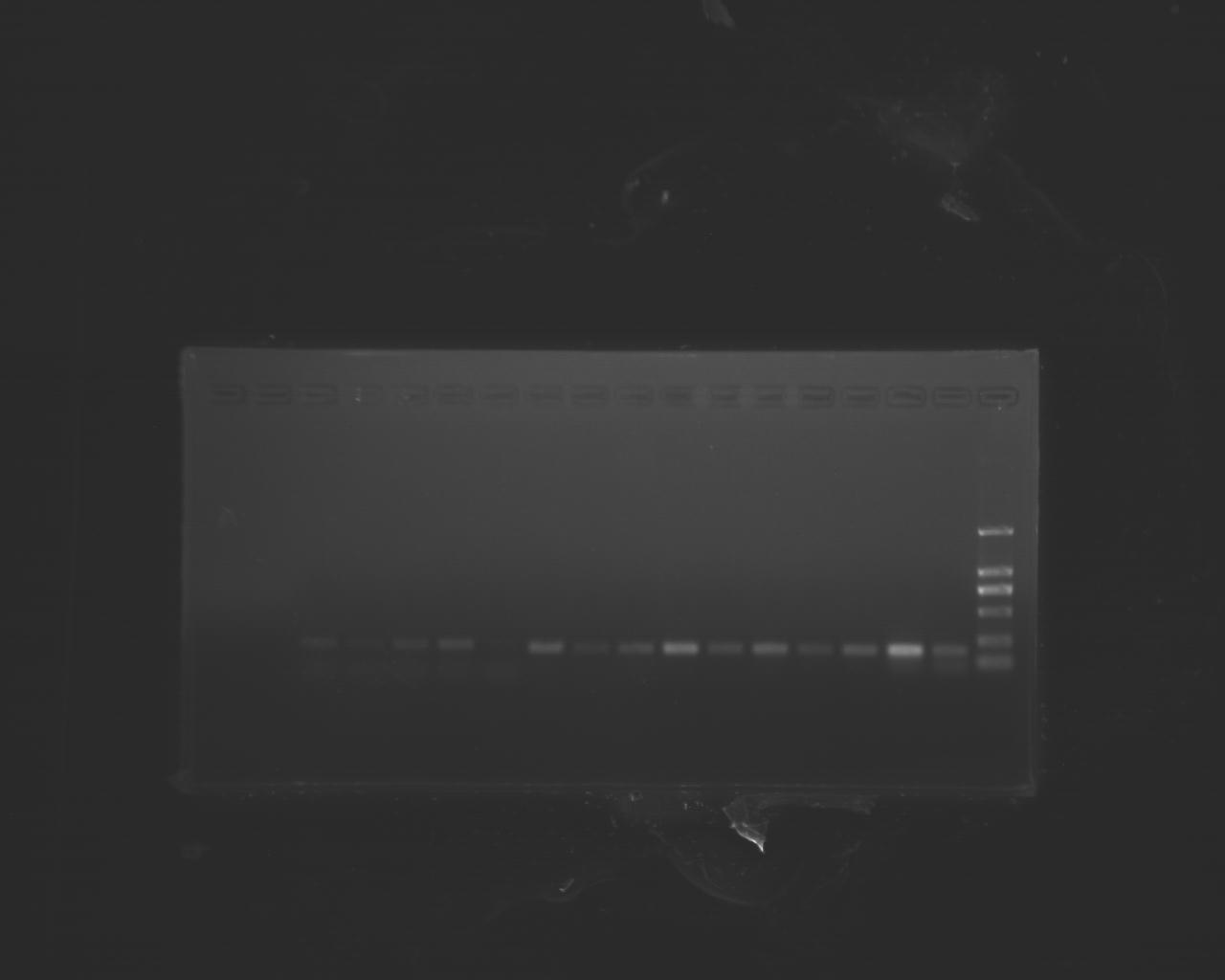


ZjbZIP11

Fruit Flower Leaves Branch Root


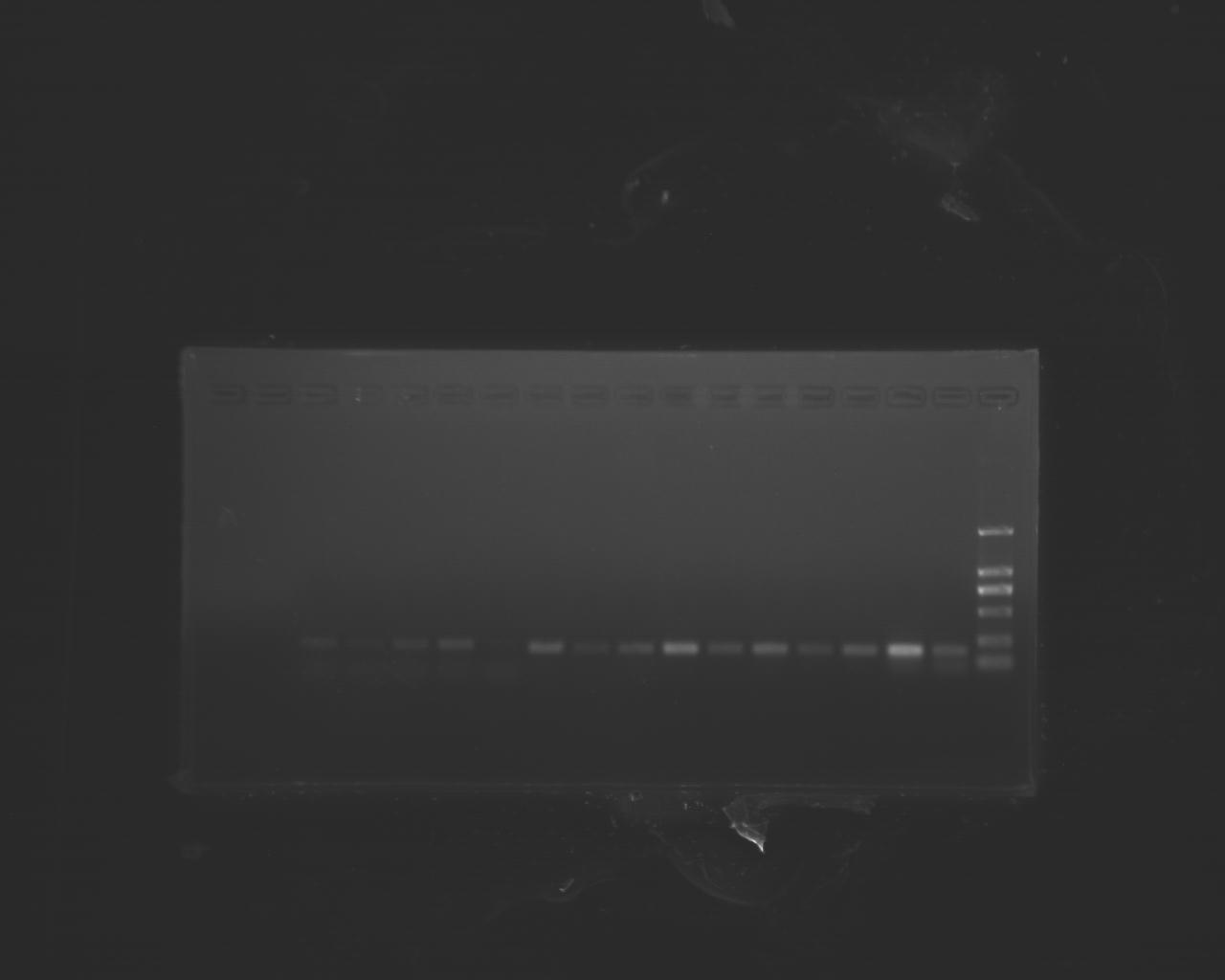


Fruit Flower Leaves Branch Root

ZjbZIP12


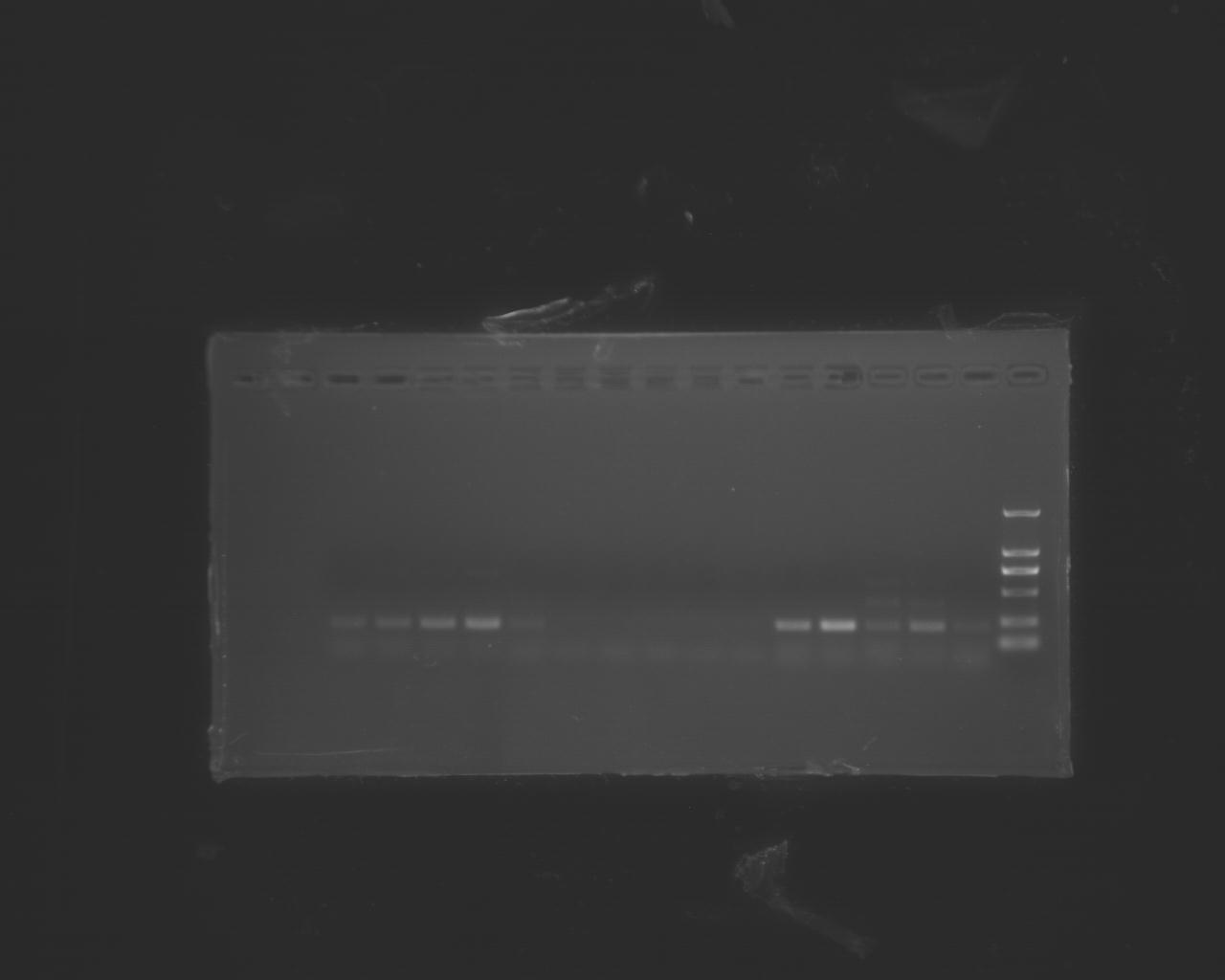


Fruit Flower Leaves Branch Root

*ZjbZIP41*


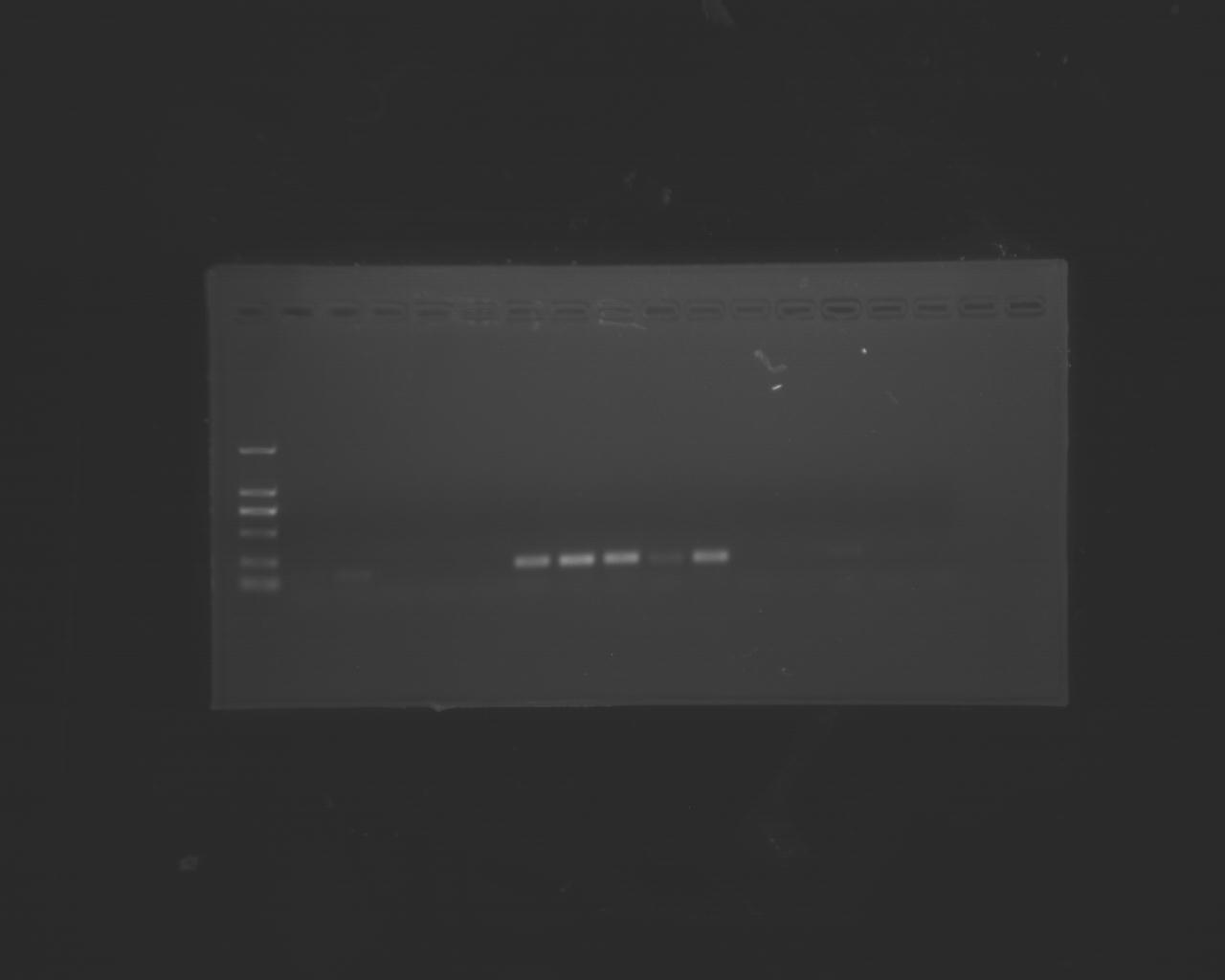


*ZjbZIP15*

Root Branch Leaves Flower Fruit


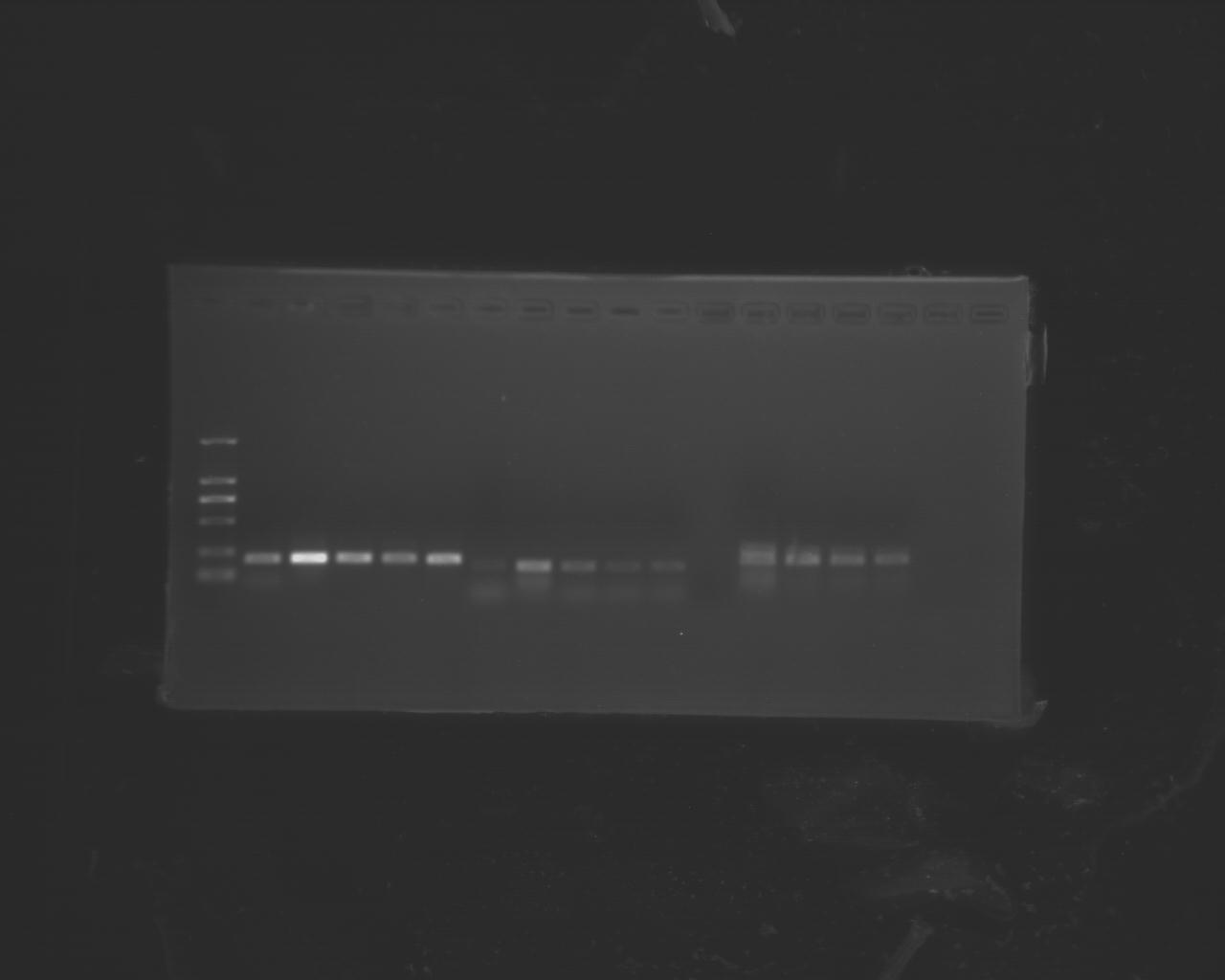


Root Branch Leaves Flower Fruit

*ZjbZIP19*


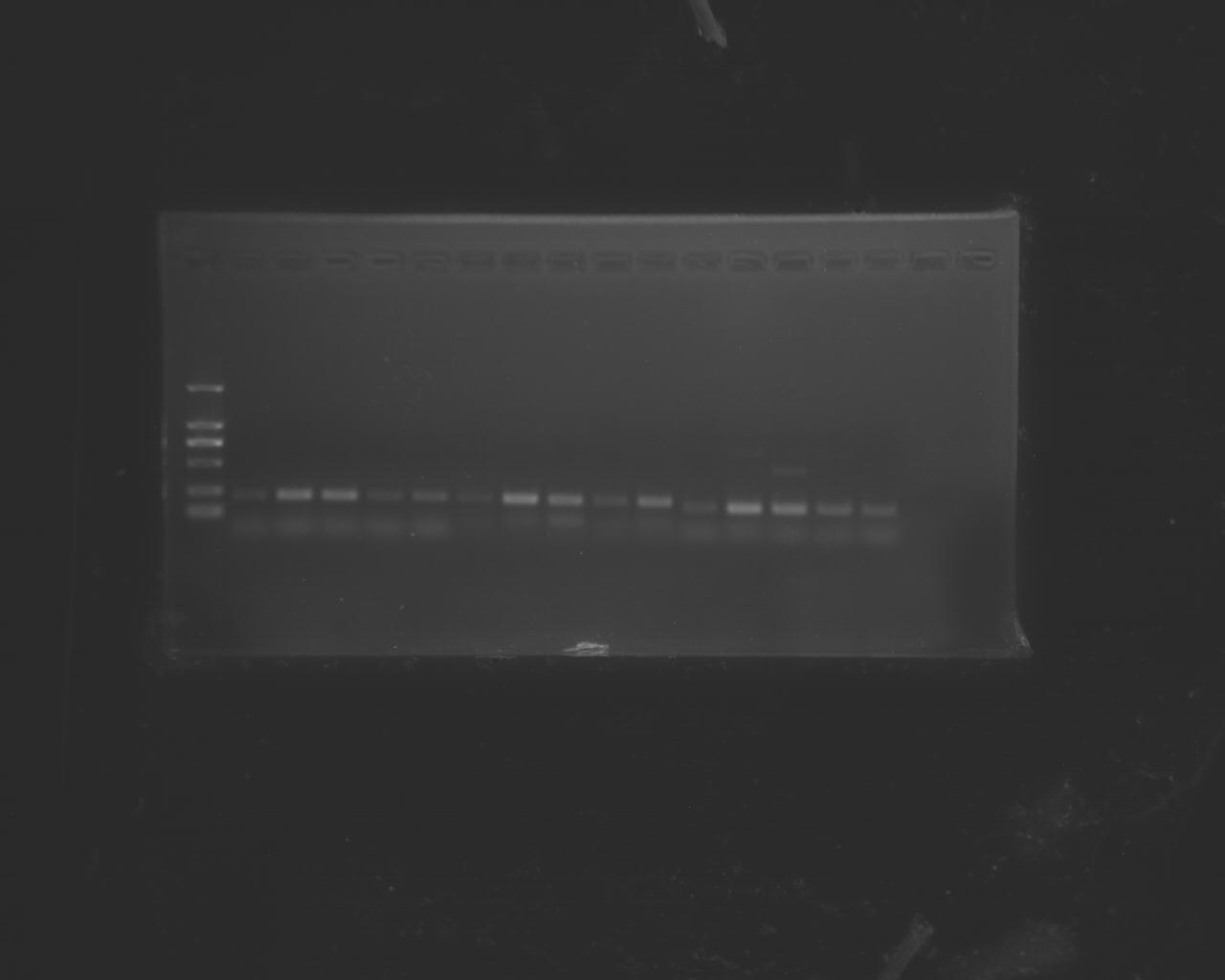


Root Branch Leaves Flower Fruit

*ZjbZIP20*


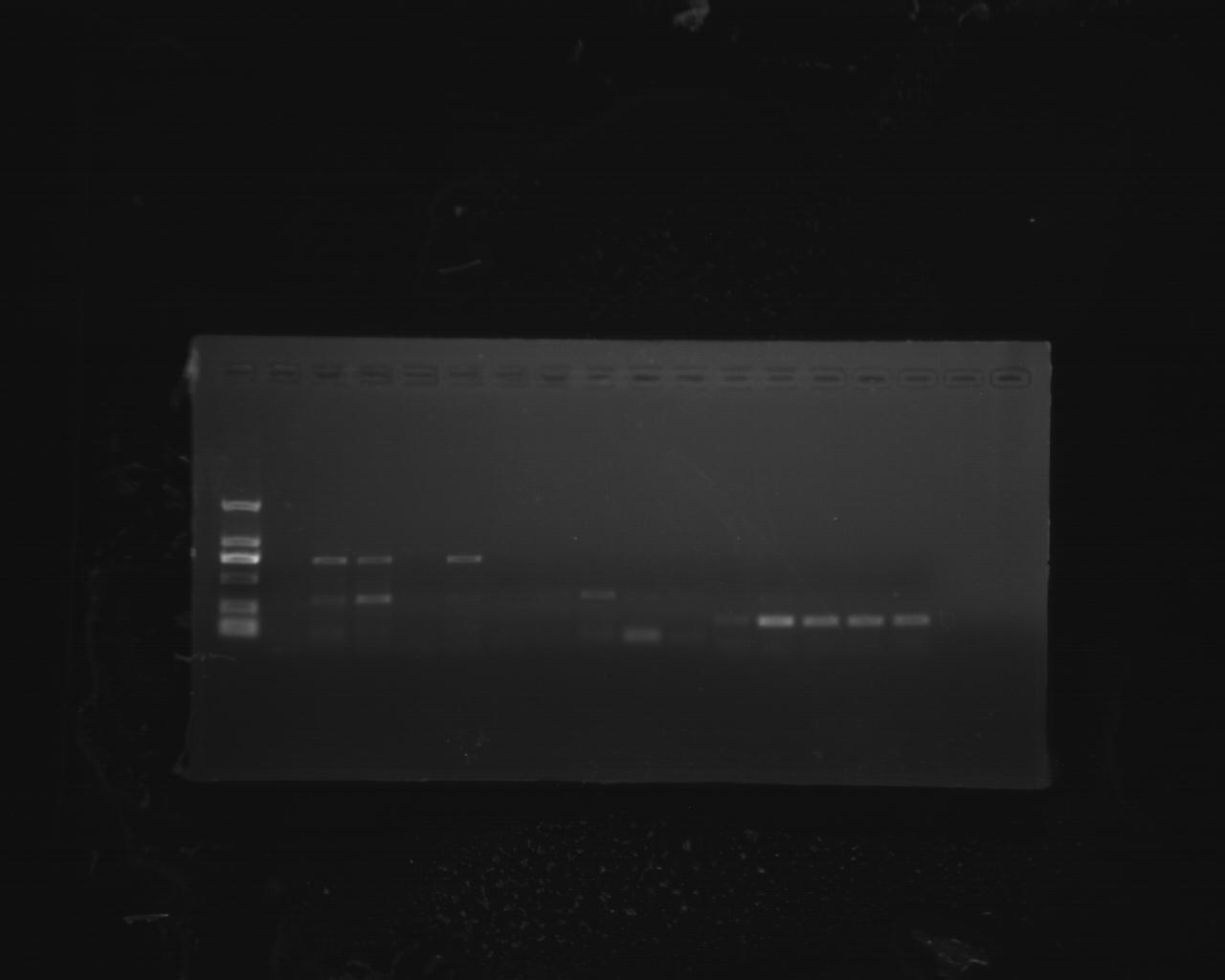


Root Branch Leaves Flower Fruit

*ZjbZIP22*


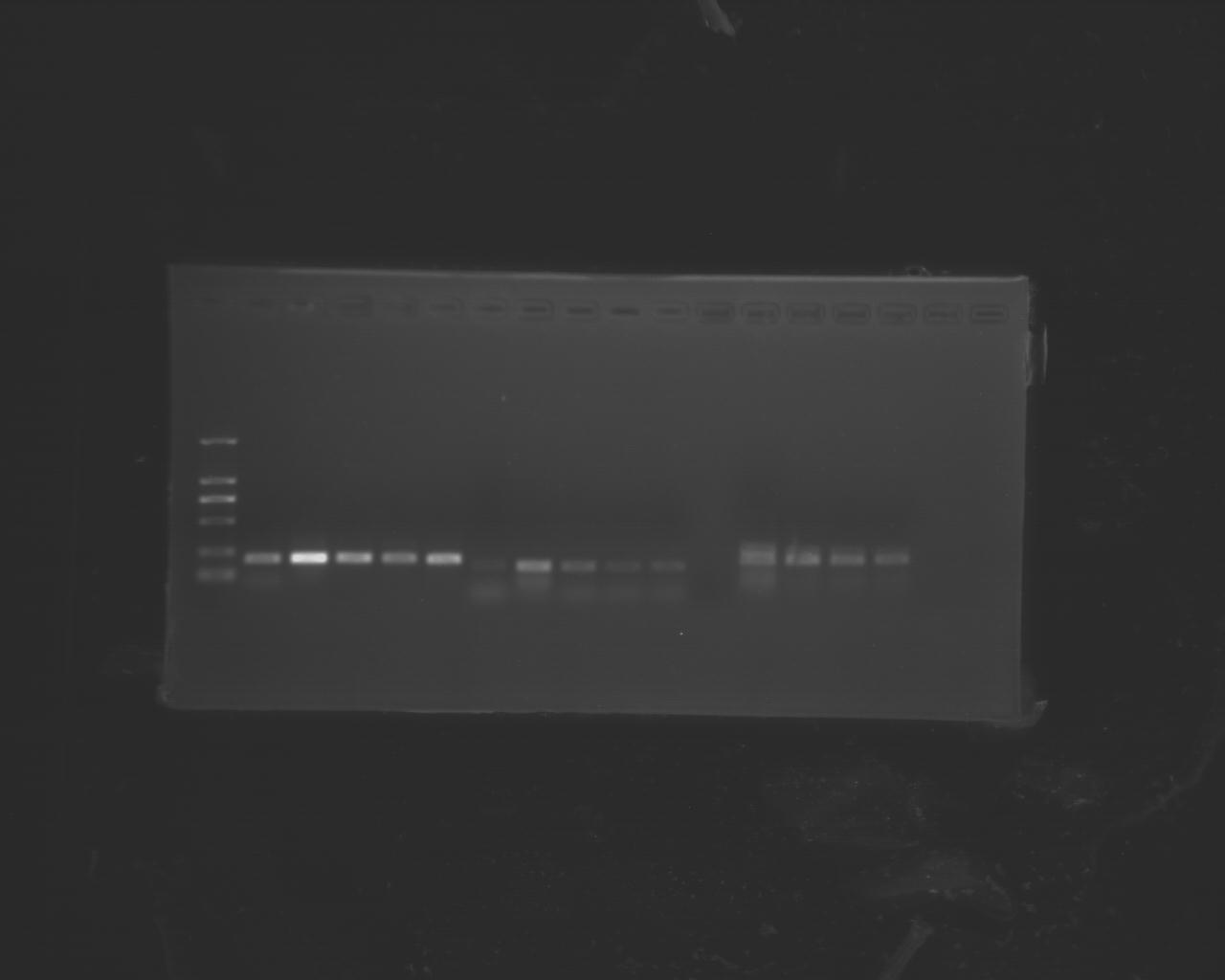


Root Branch Leaves Flower Fruit

*ZjbZIP24*


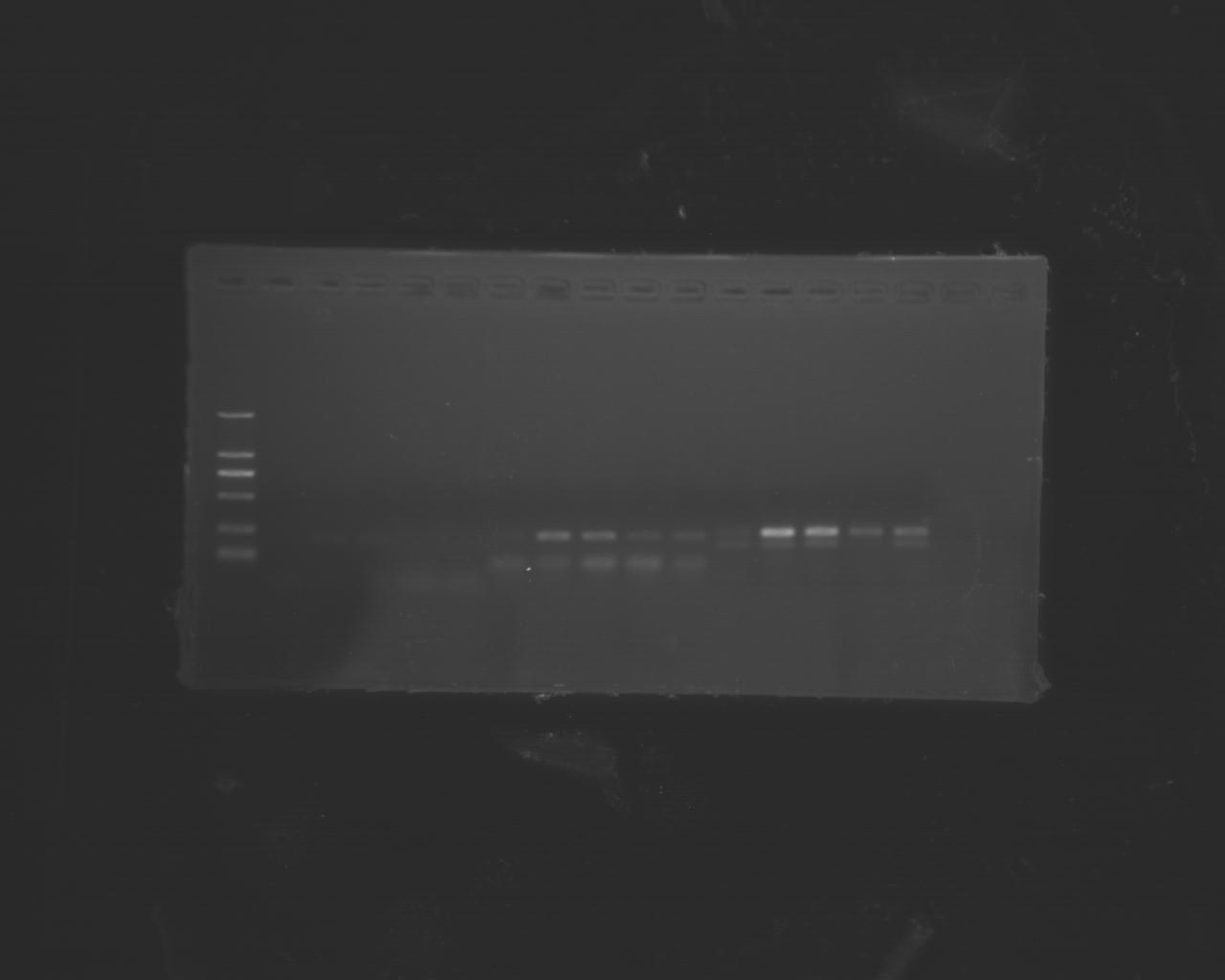


Root Branch Leaves Flower Fruit

*ZjbZIP25*


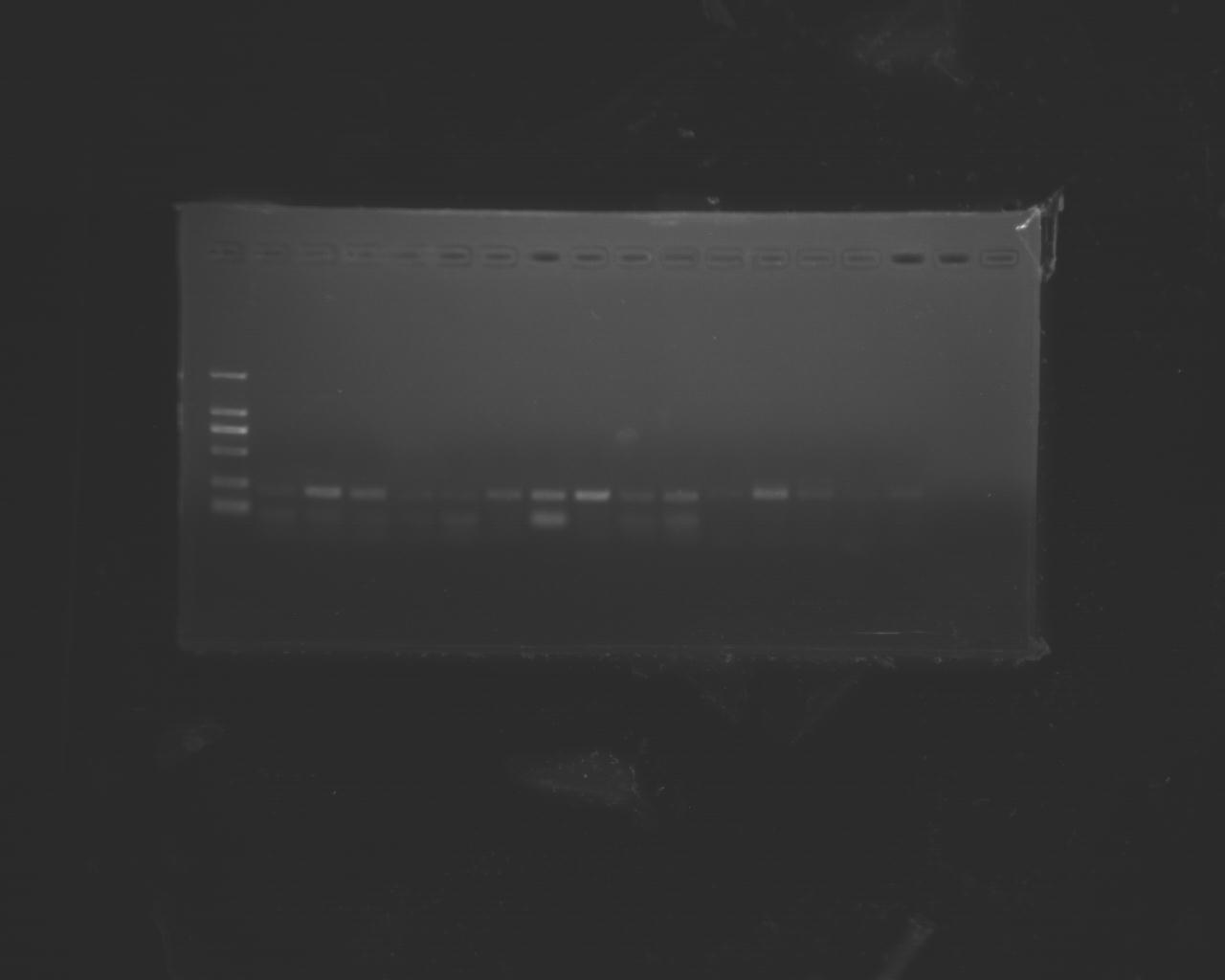


Root Branch Leaves Flower Fruit

ZjbZIP26


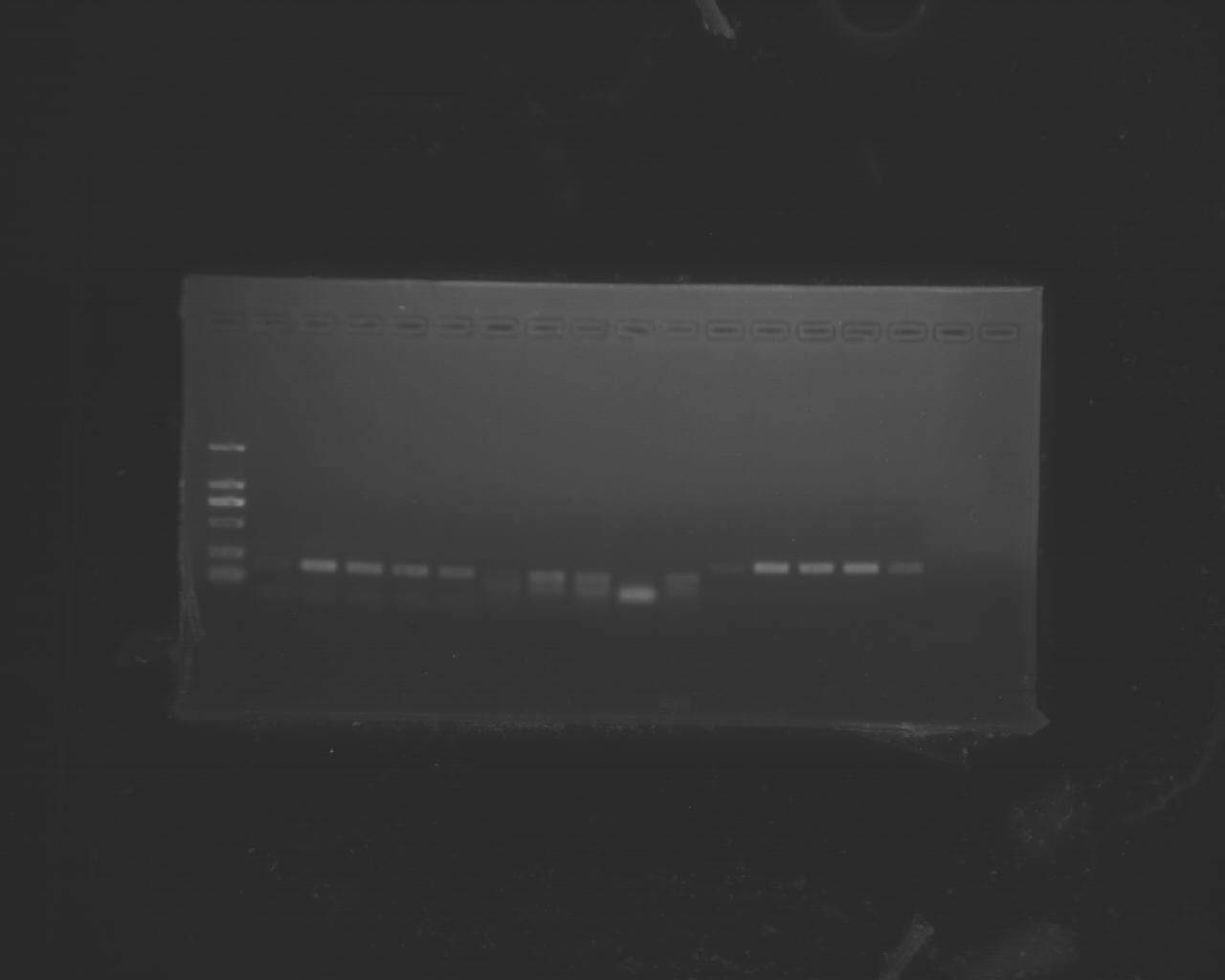


Root Branch Leaves Flower Fruit

ZjbZIP27


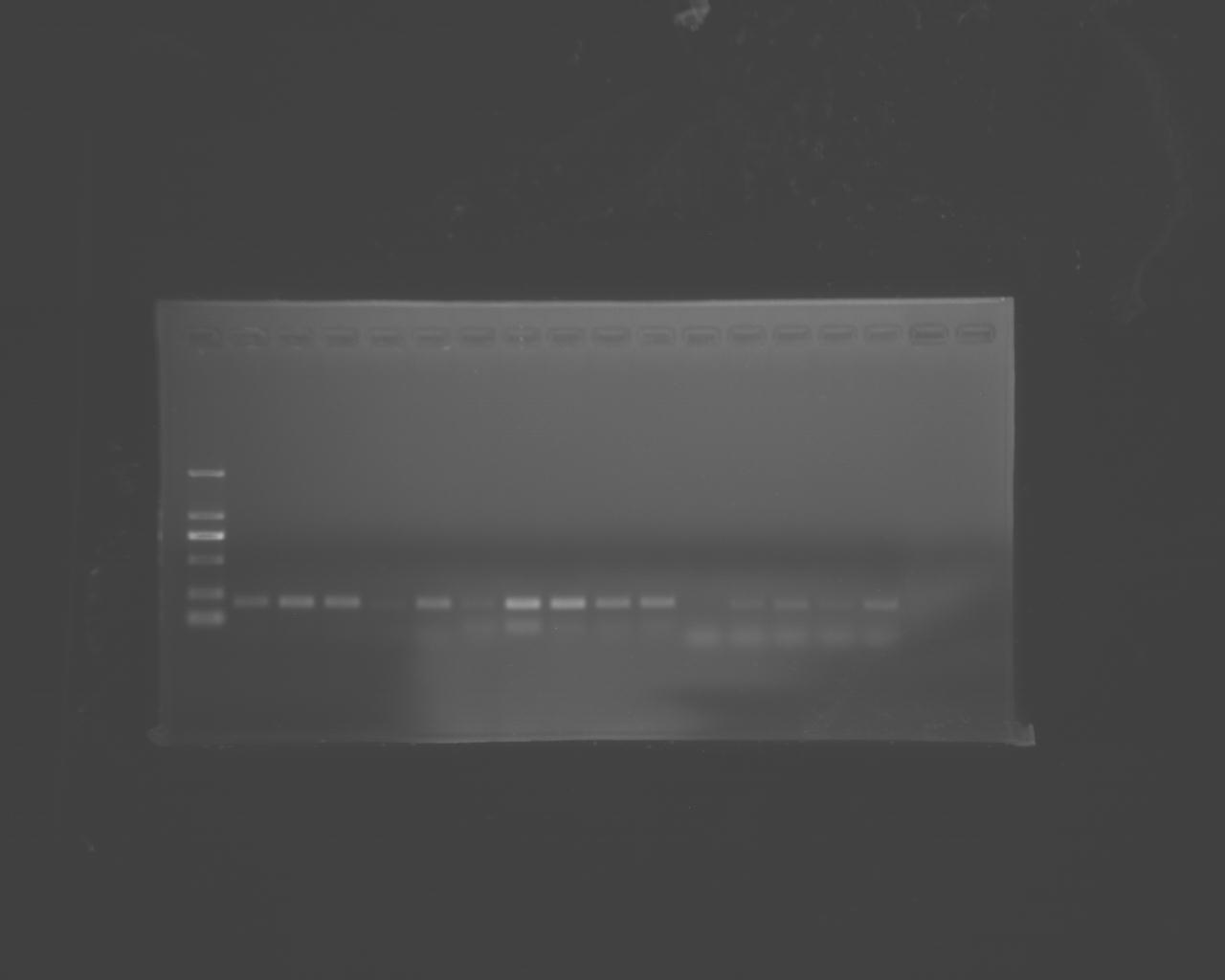


Root Branch Leaves Flower Fruit

ZjbZIP28


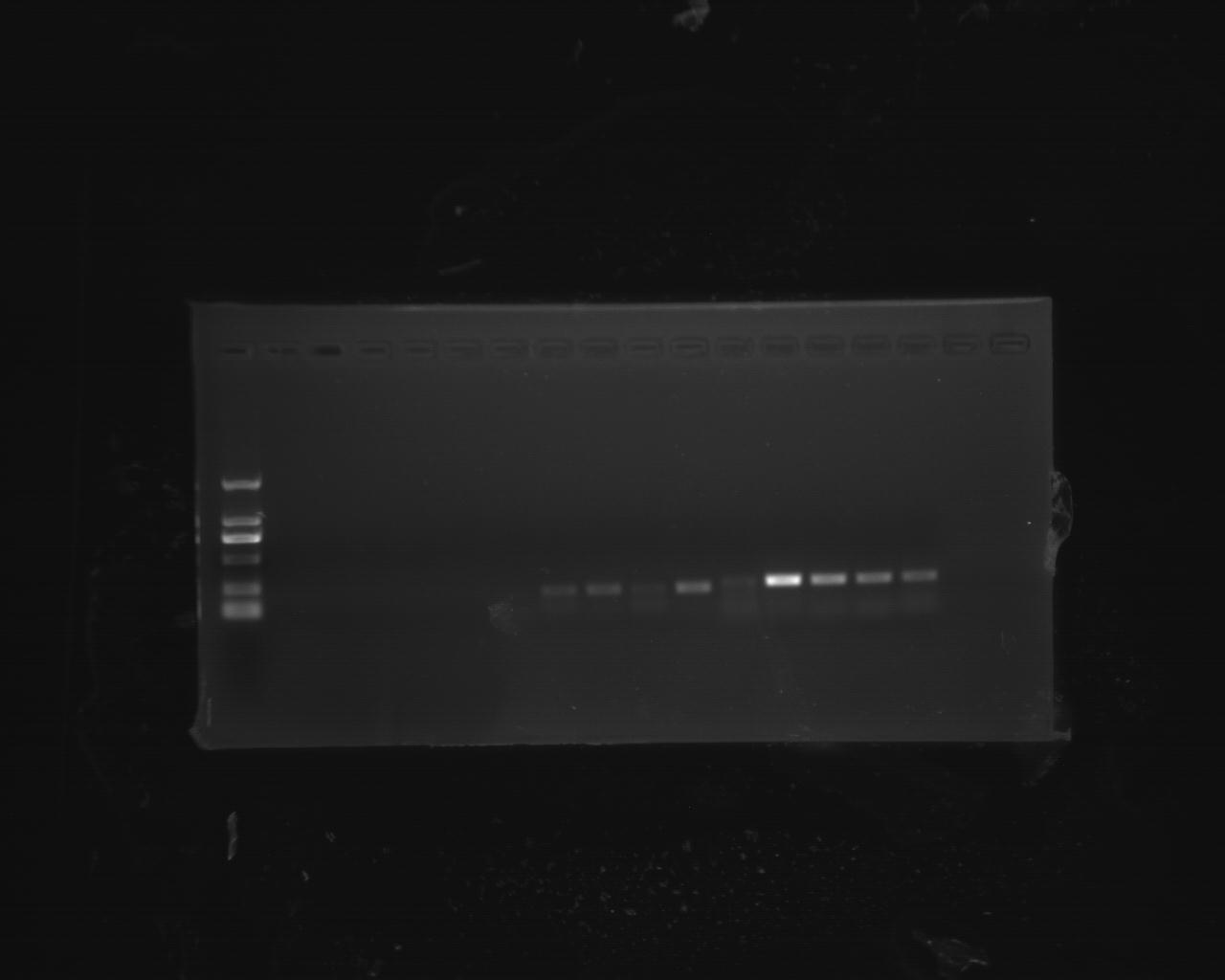


Root Branch Leaves Flower Fruit

*ZjbZIP29*


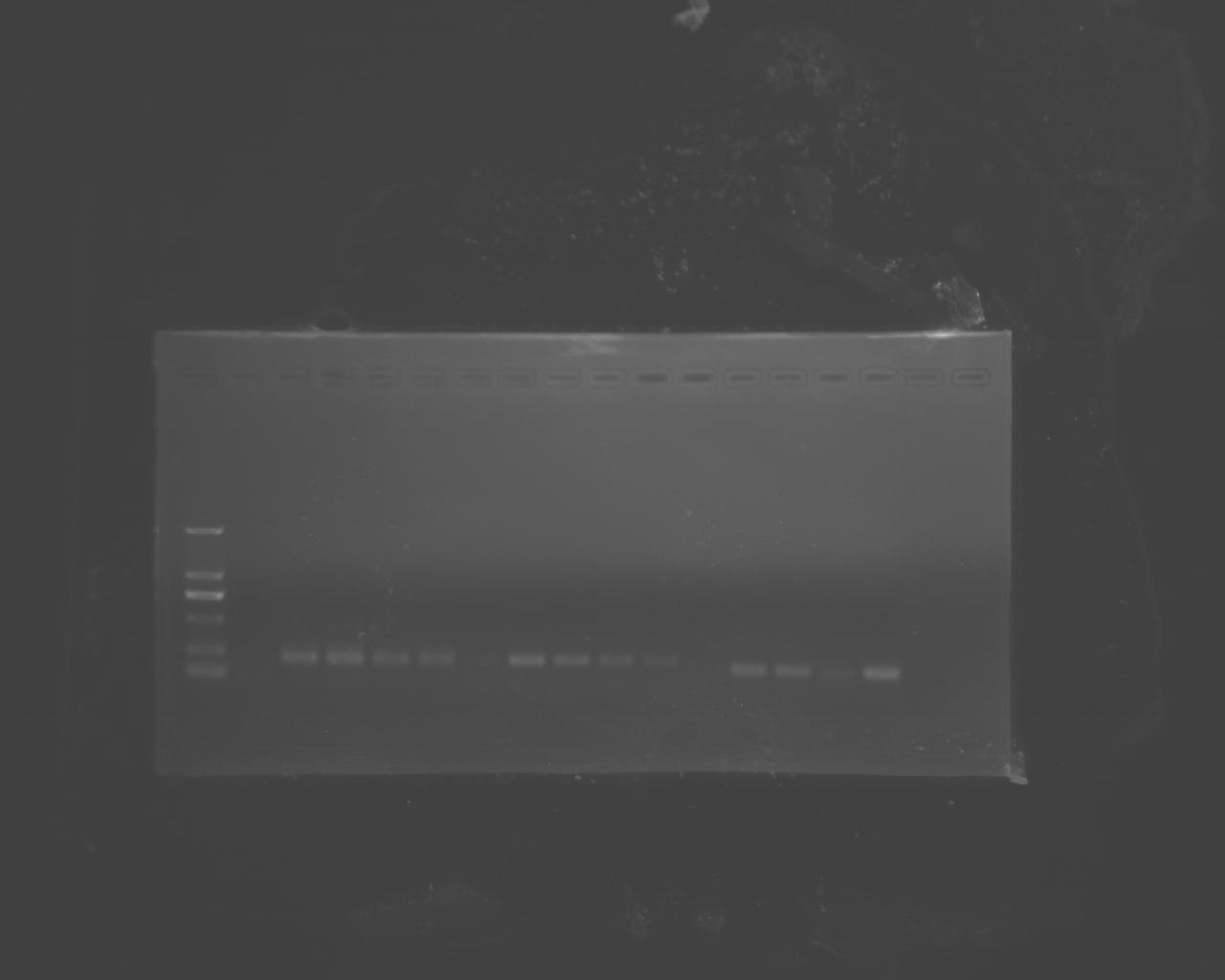


Root Branch Leaves Flower Fruit

*ZjbZIP30*


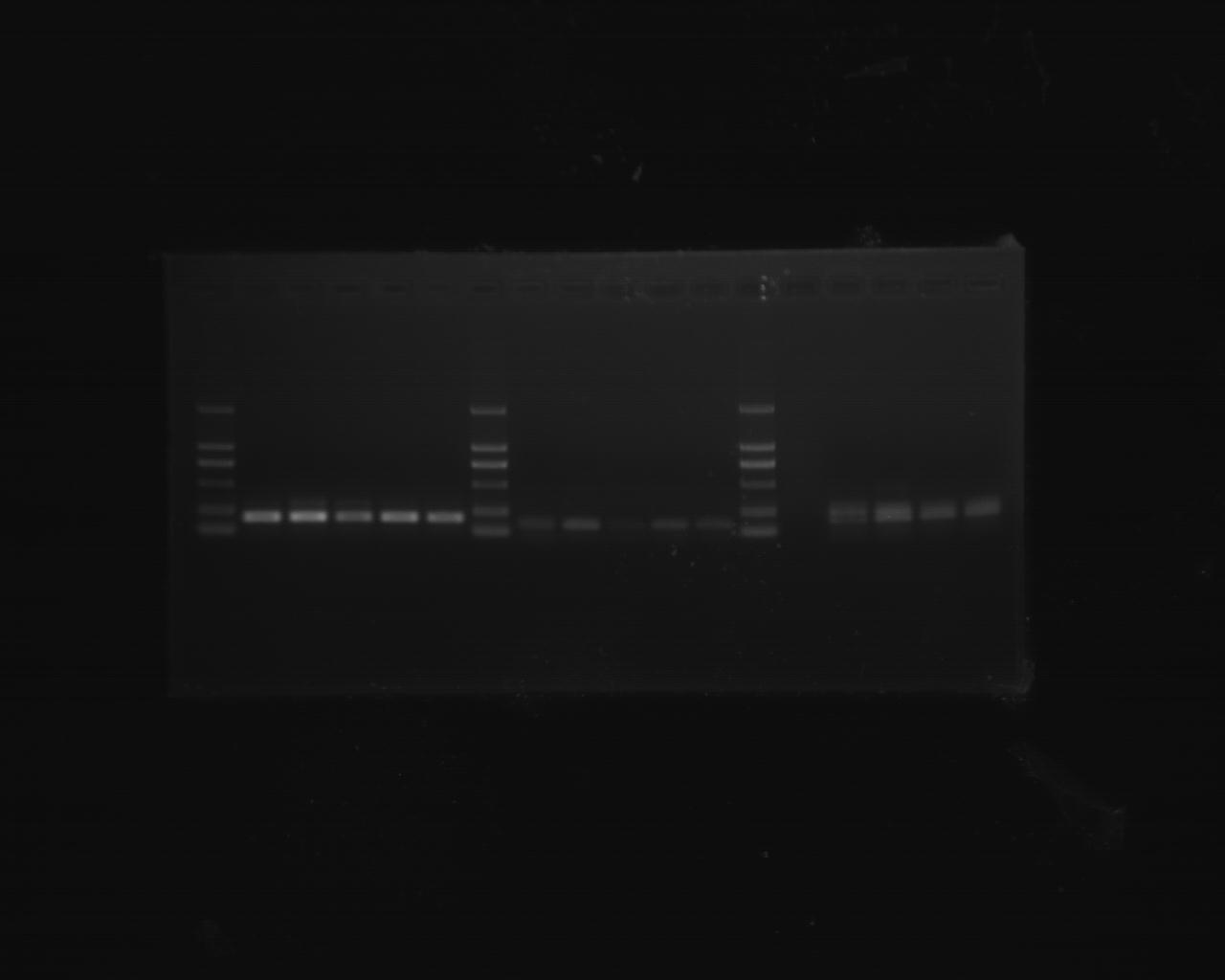


Root Branch Leaves Flower Fruit

*ZjbZP31*


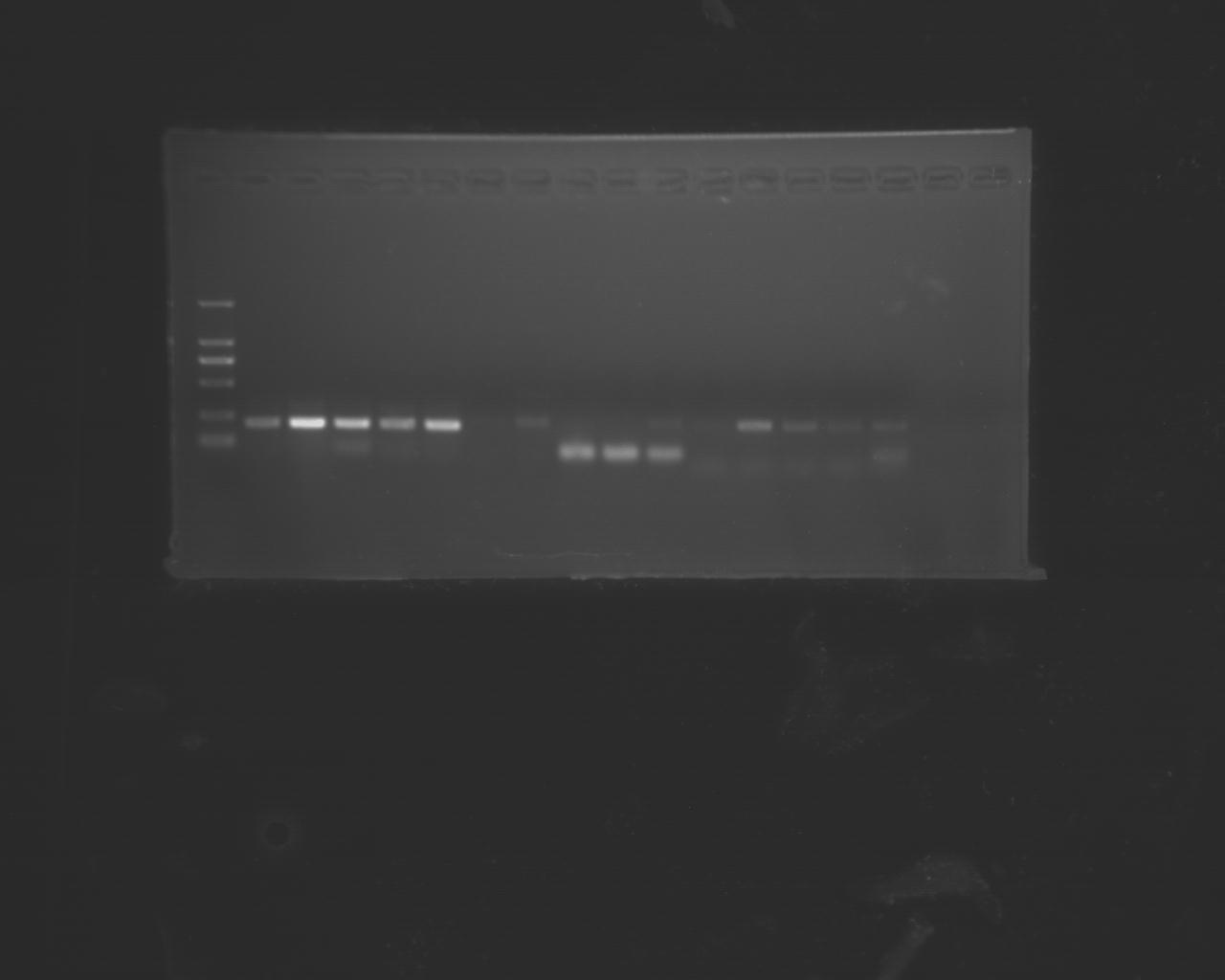


Root Branch Leaves Flower Fruit

*ZjbZIP33*


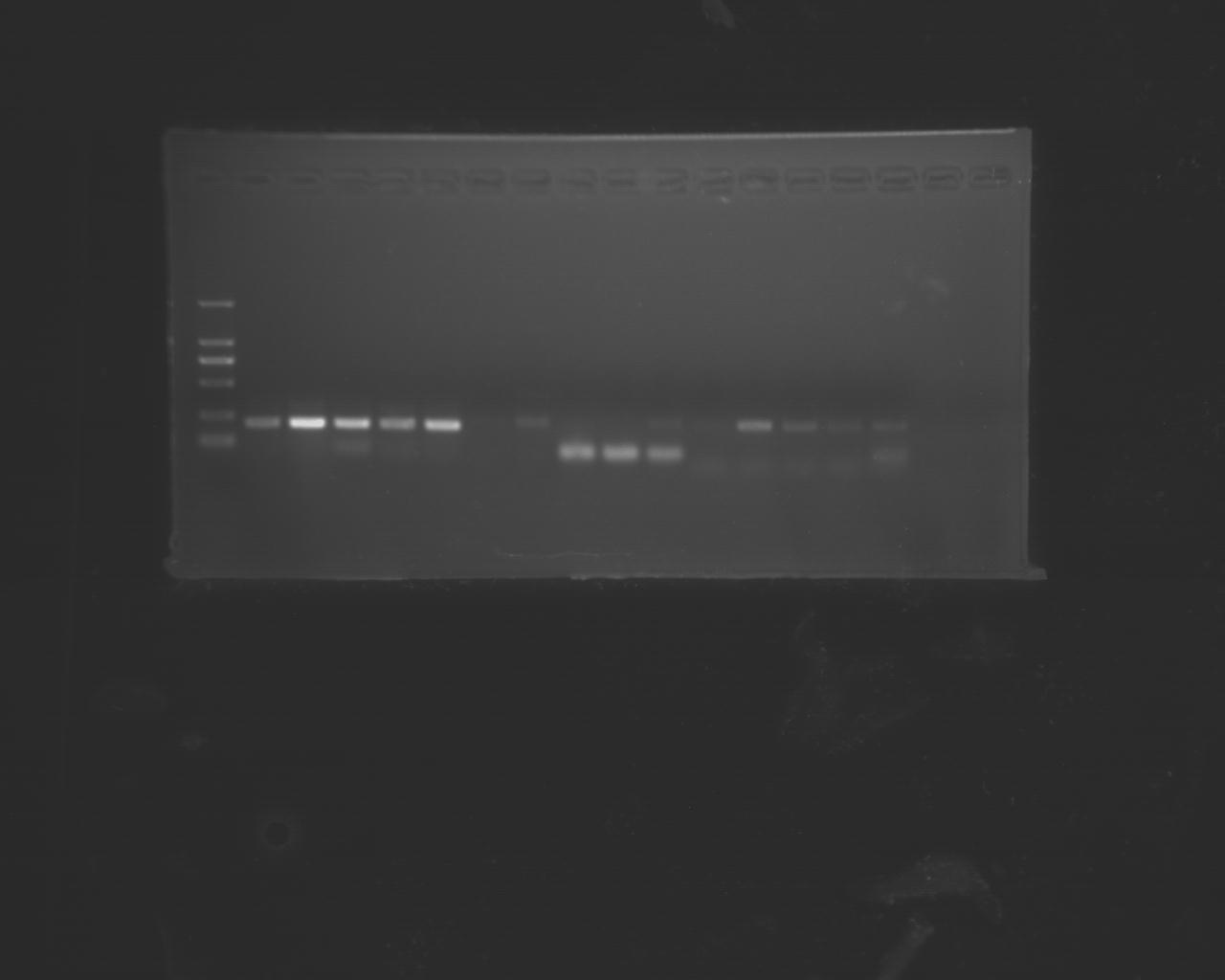


Root Branch Leaves Flower Fruit

*ZjbZIP34*


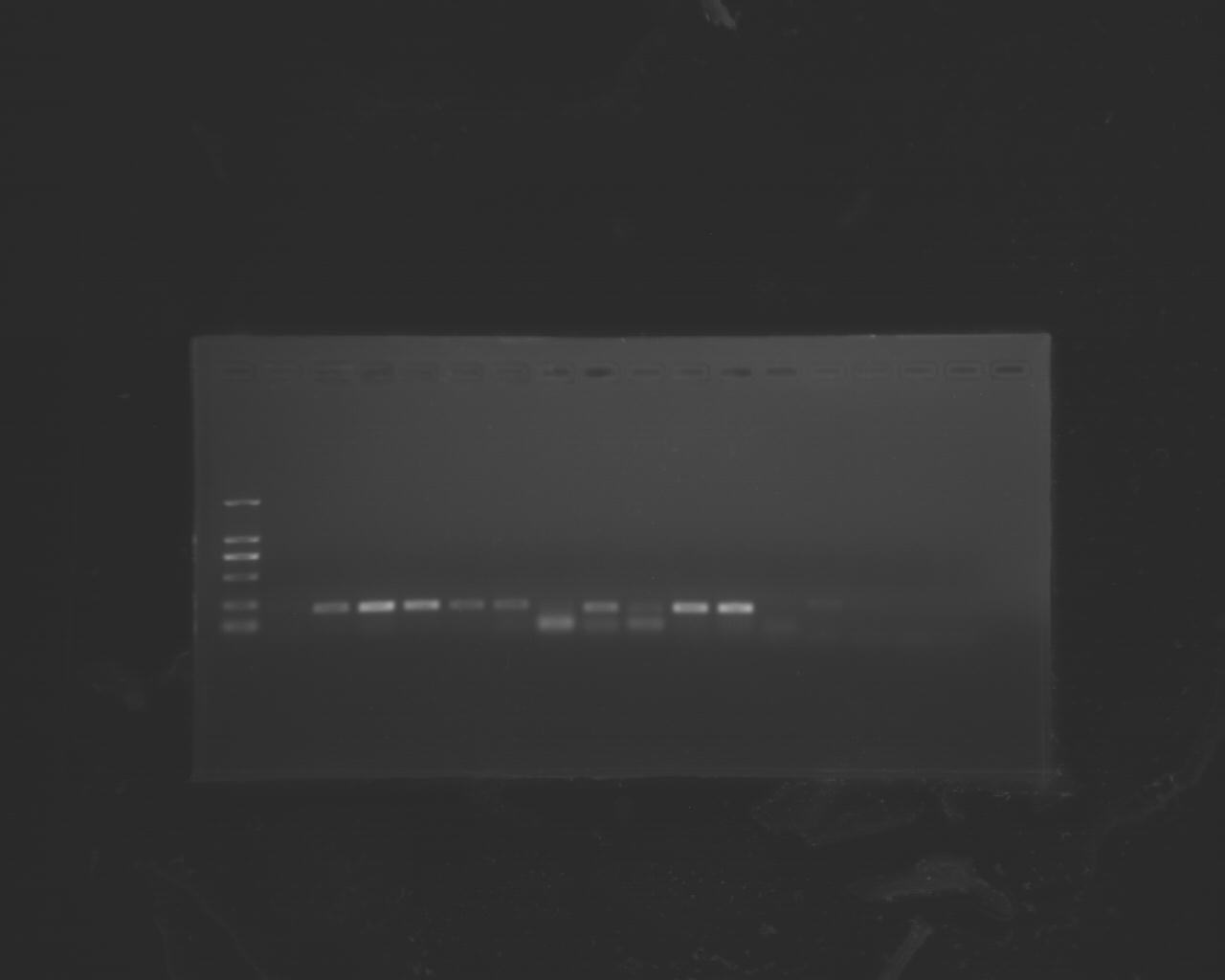


Root Branch Leaves Flower Fruit

ZjbZIP35


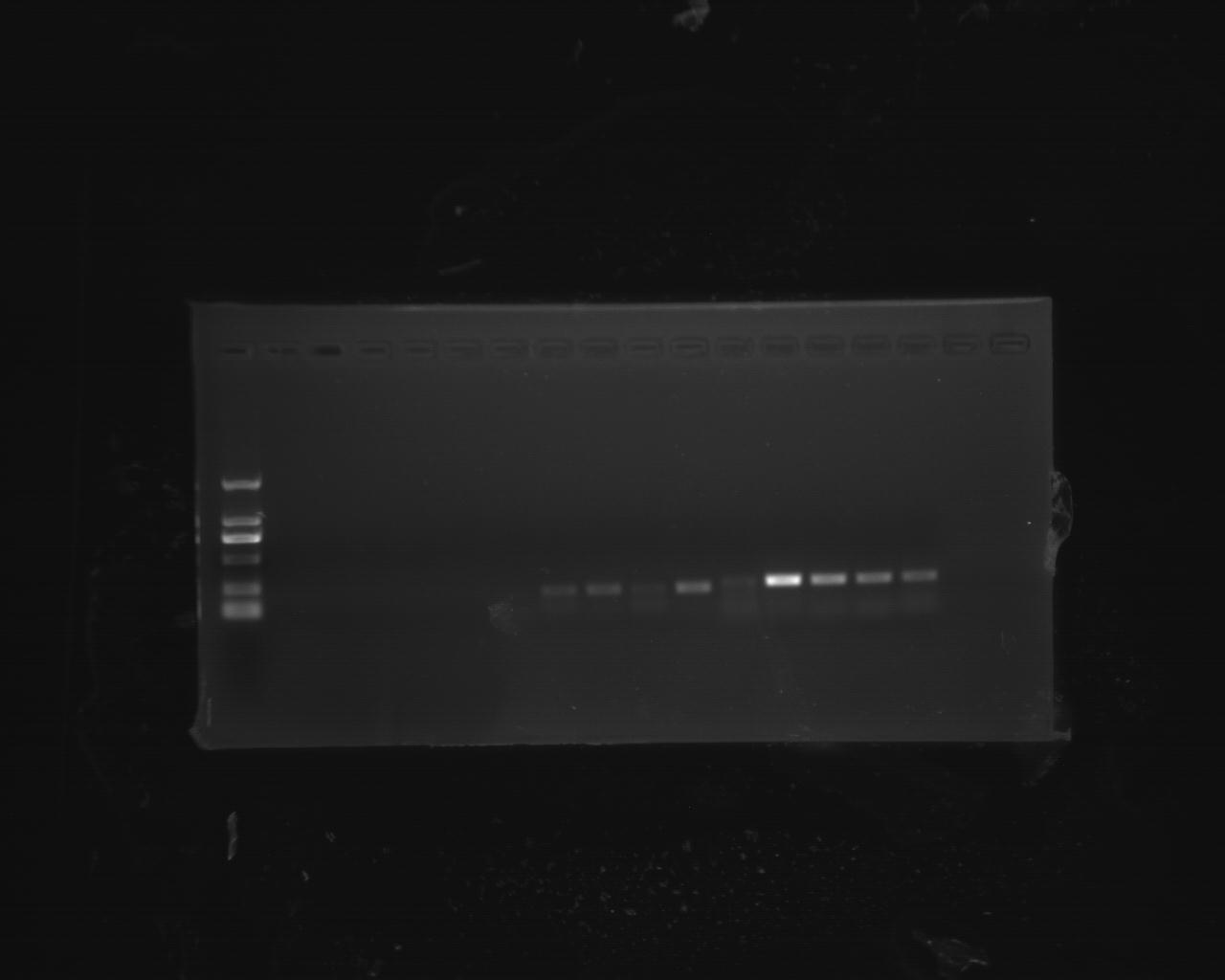


Root Branch Leaves Flower Fruit

ZjbZIP36


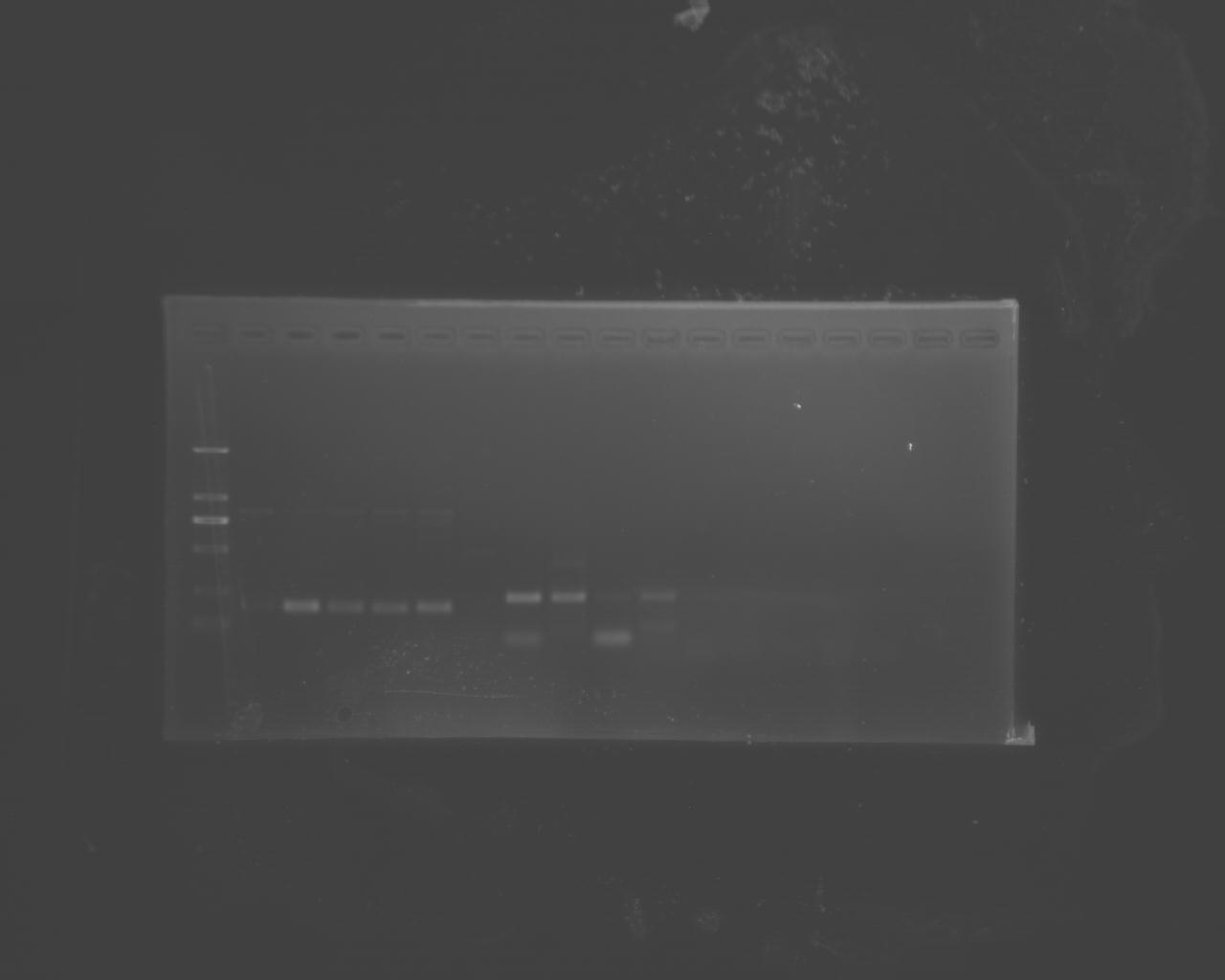


Root Branch Leaves Flower Fruit

*ZjbZIP38*


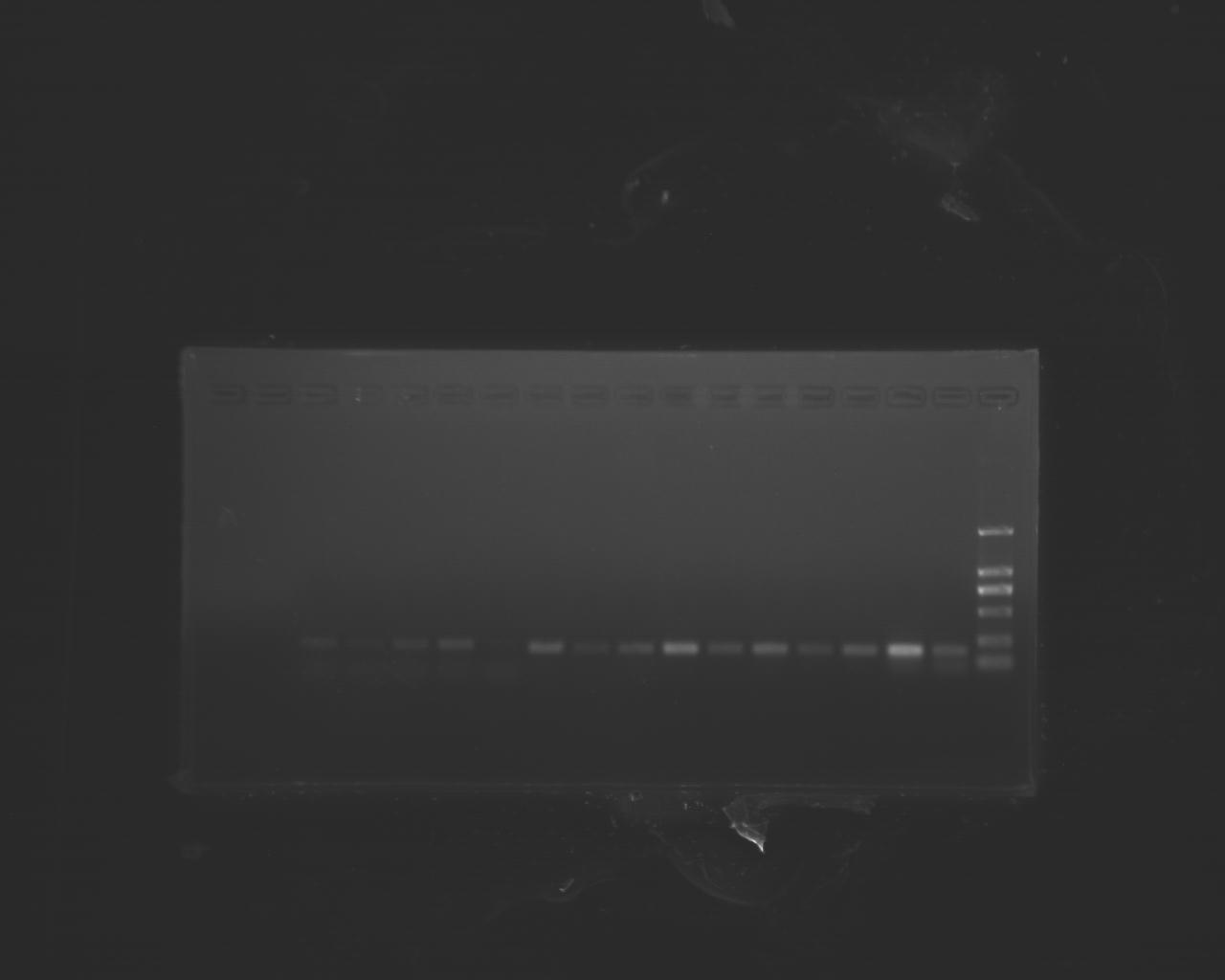


Fruit Flower Leaves Branch Root

*ZjbZIP40*


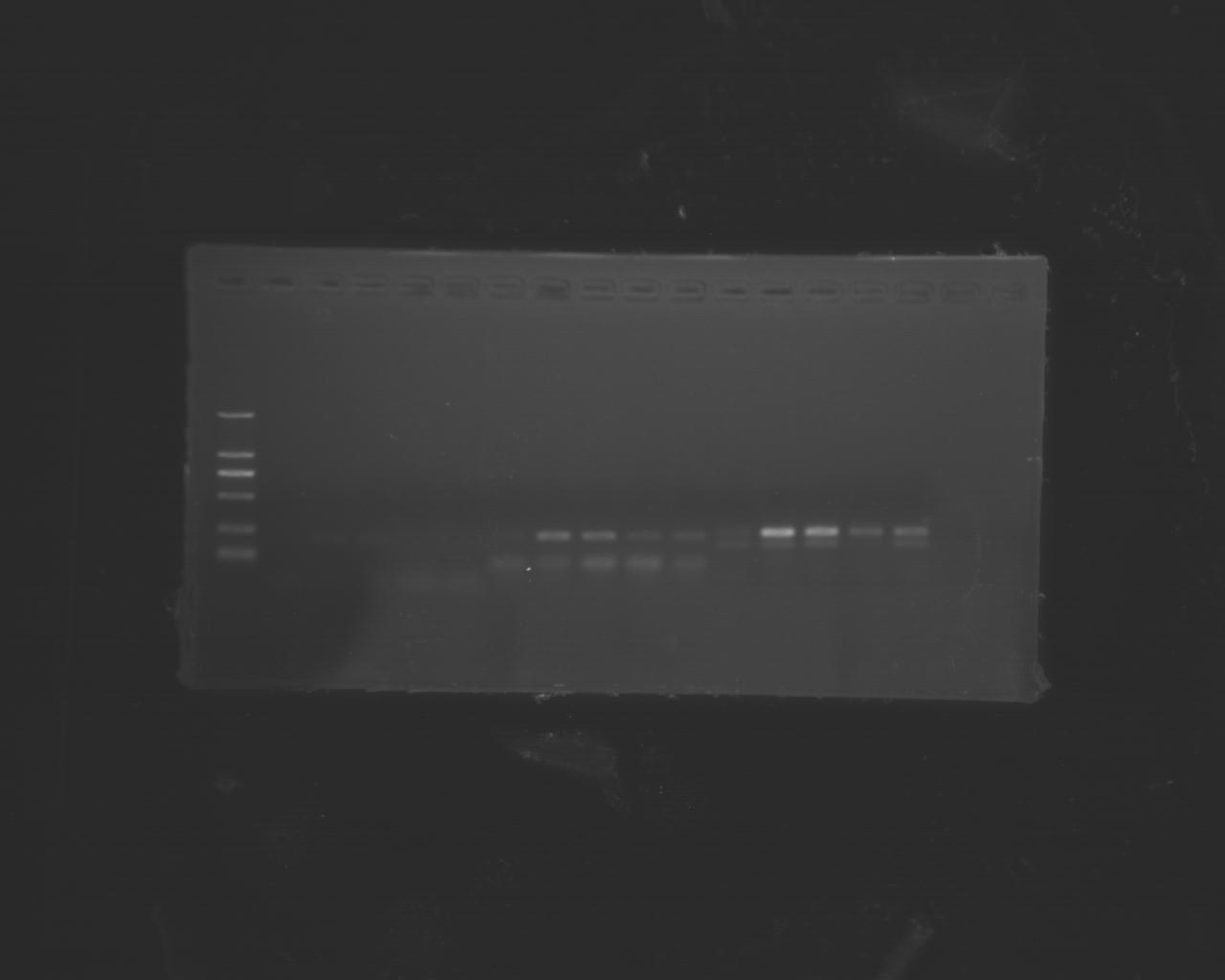


Root Branch Leaves Flower Fruit

ZjbZIP42


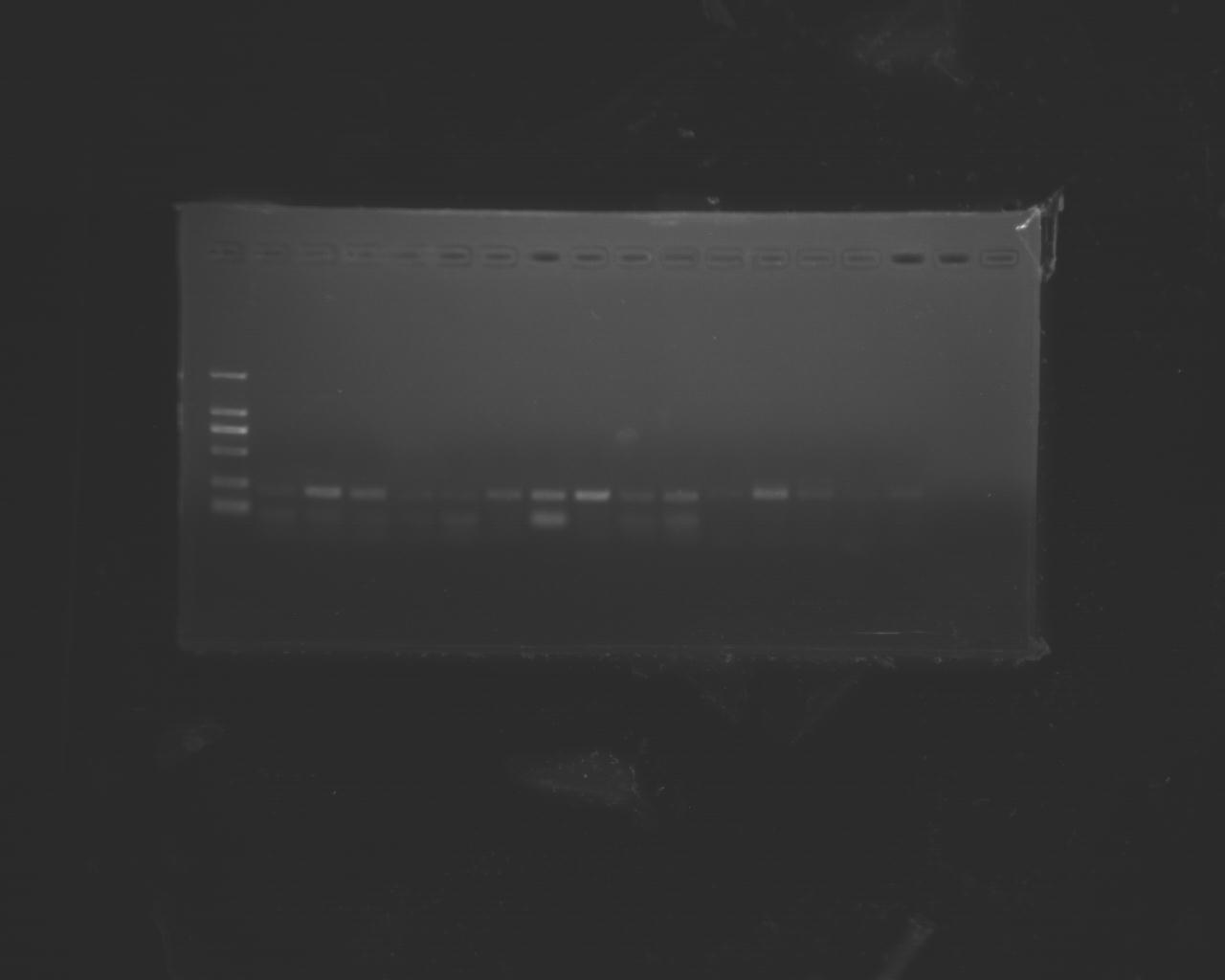


Root Branch Leaves Flower Fruit

ZjbZIP45

**Expression patterns of the genes in five tissues of jujube by RT-PCR.**


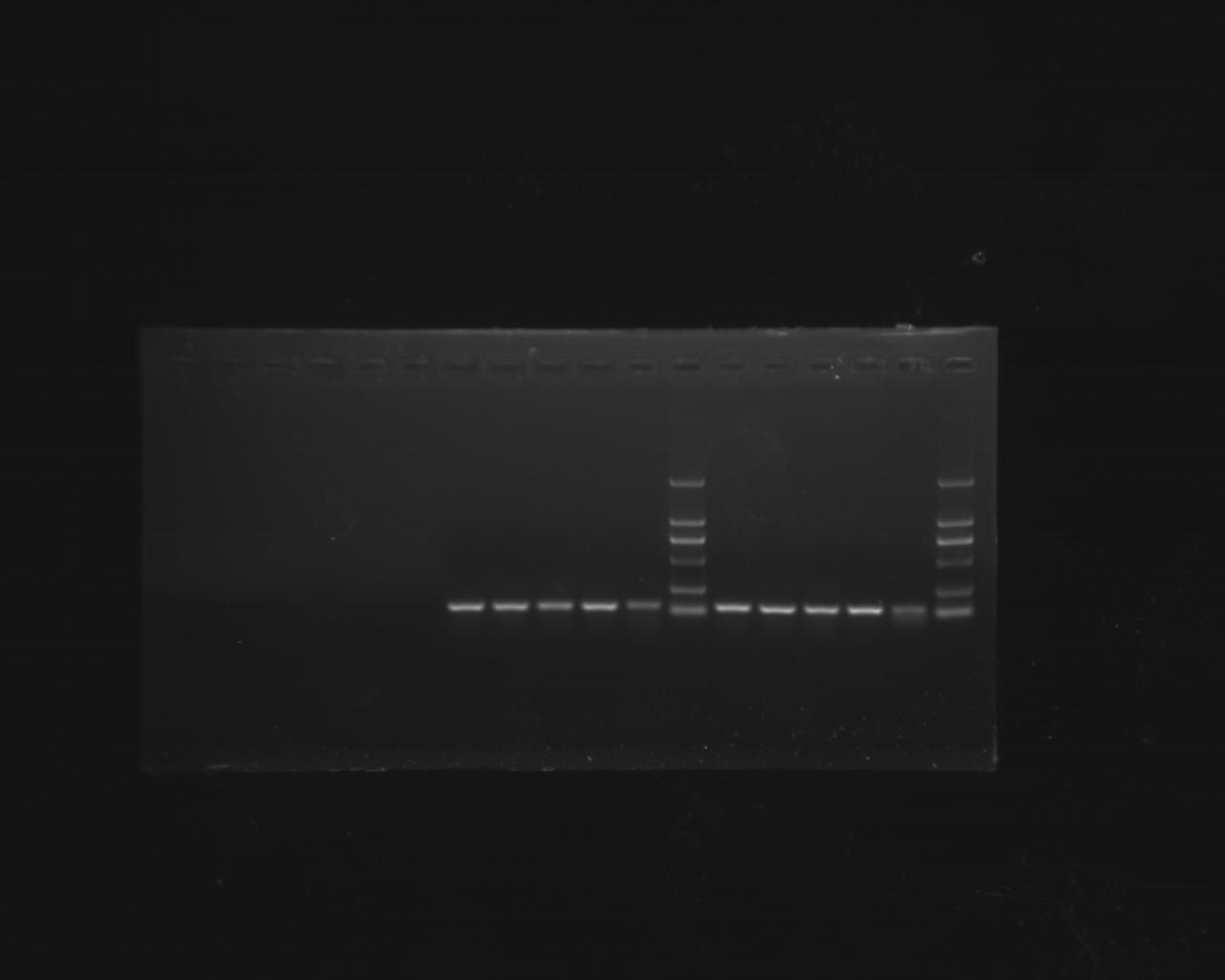


Fruit Flower Leaves Branch Root

***ZjACT***


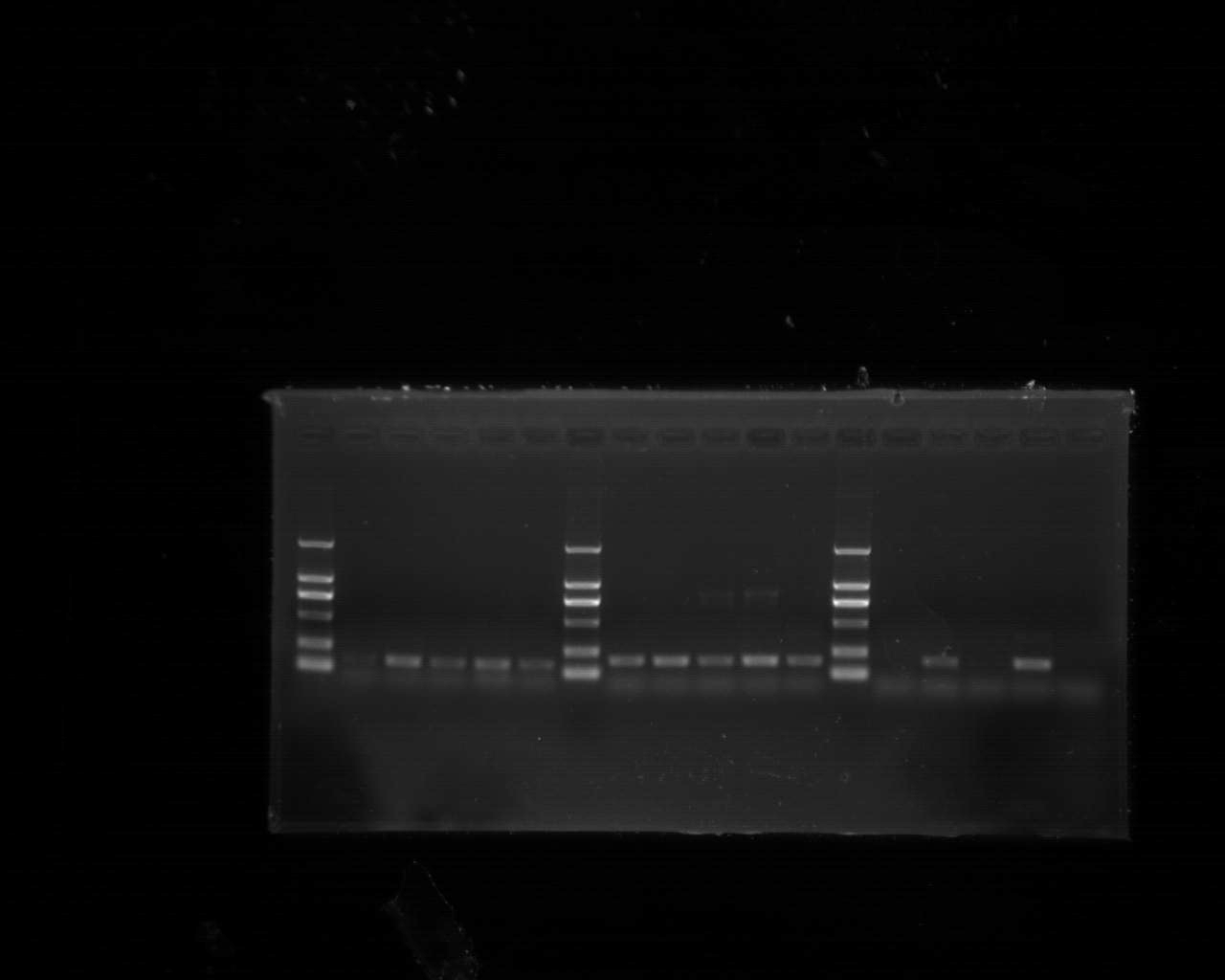


Root Branch Leaves Flower Fruit

*ZjbZIP2*


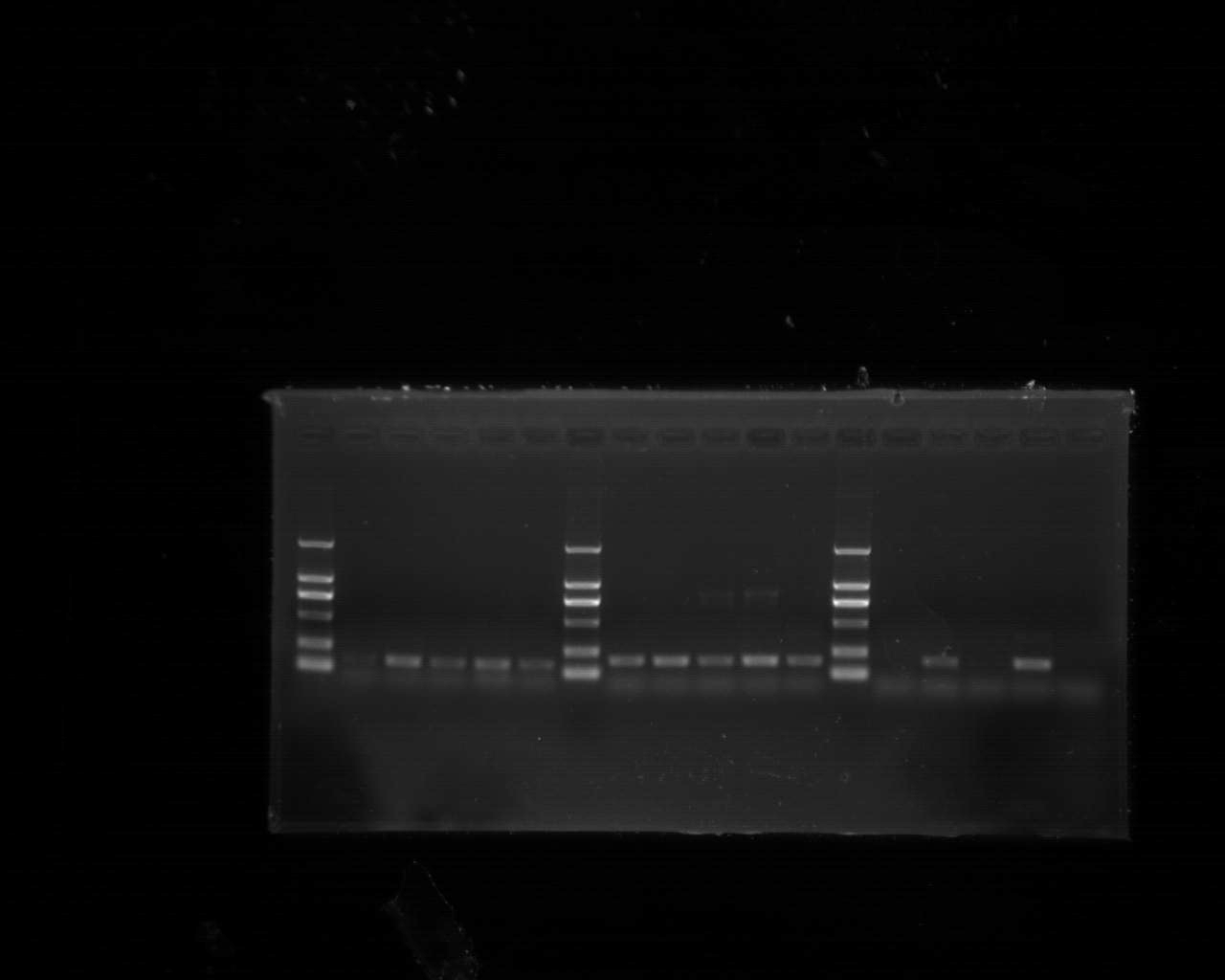


Root Branch Leaves Flower Fruit

*ZjbZIP3*


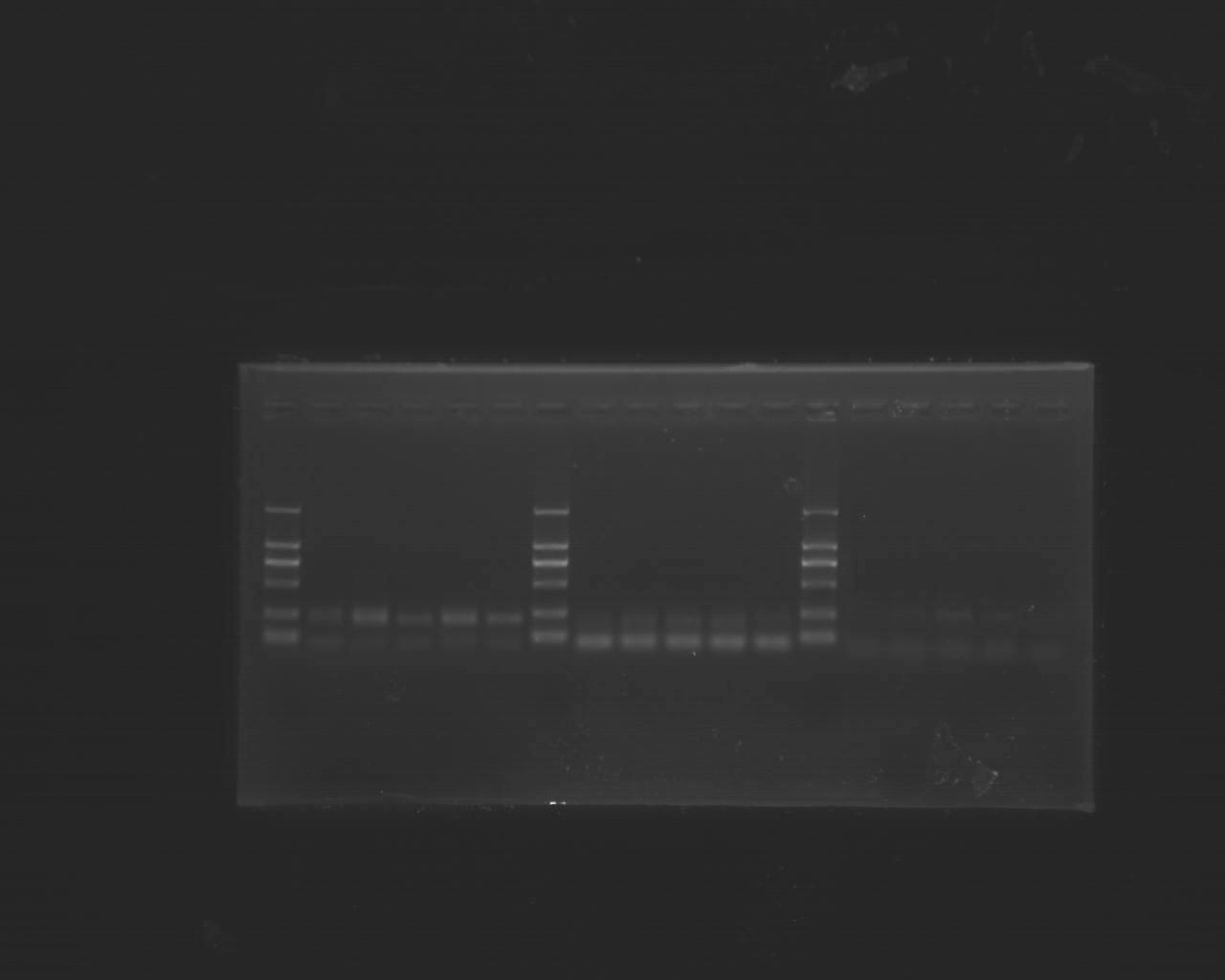


Root Branch Leaves Flower Fruit

*ZjbZIP4*


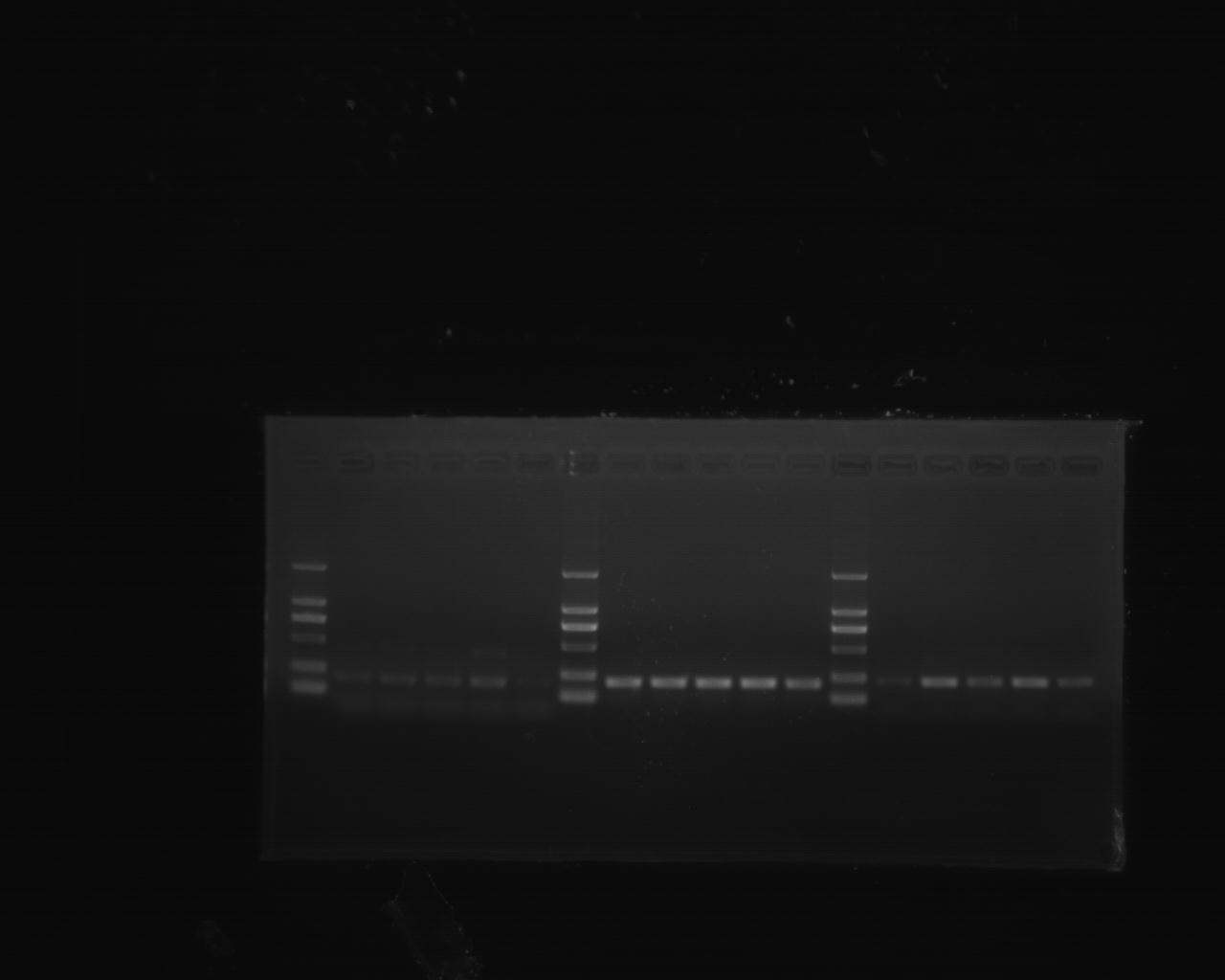


Root Branch Leaves Flower Fruit

*ZjbZIP5*


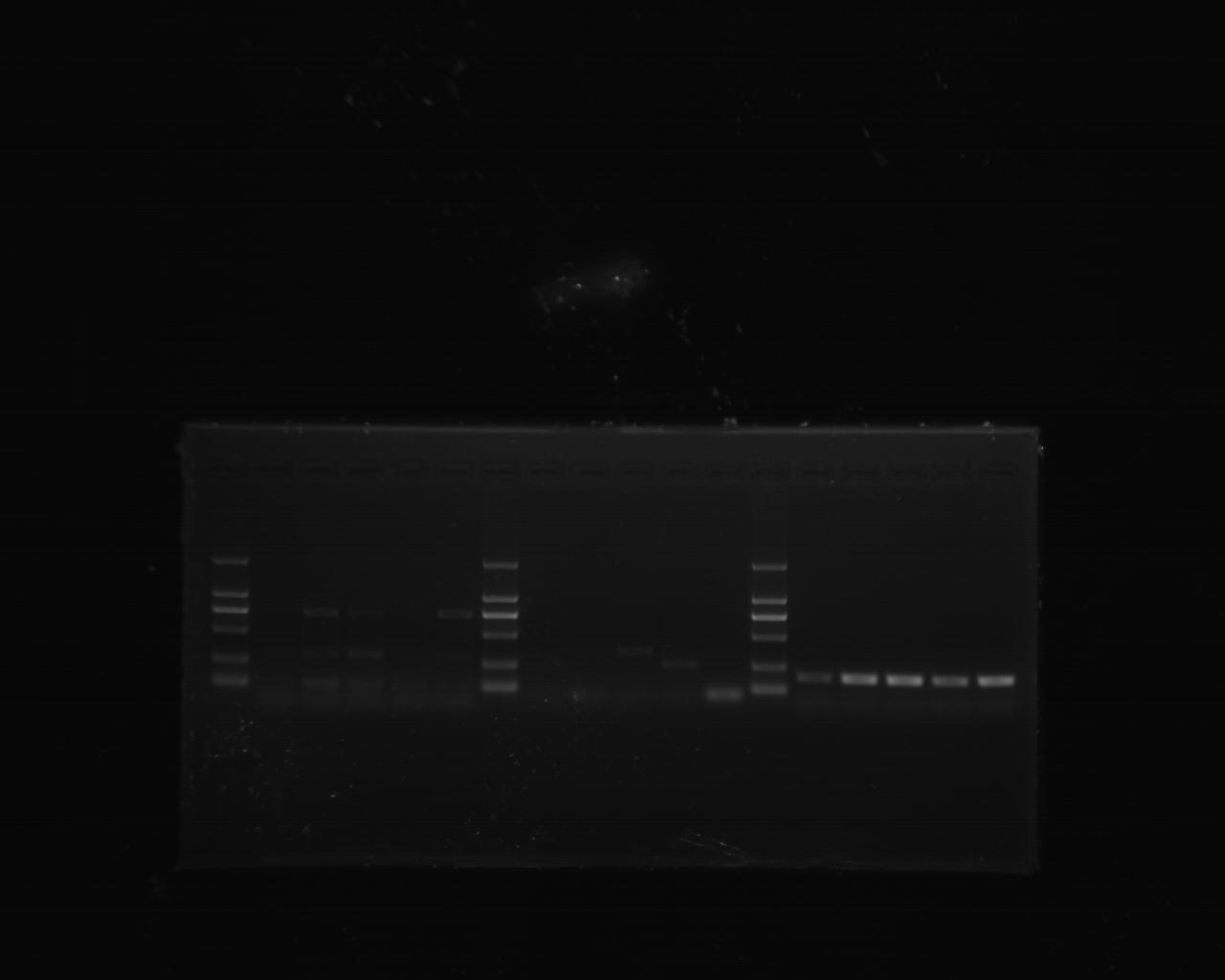

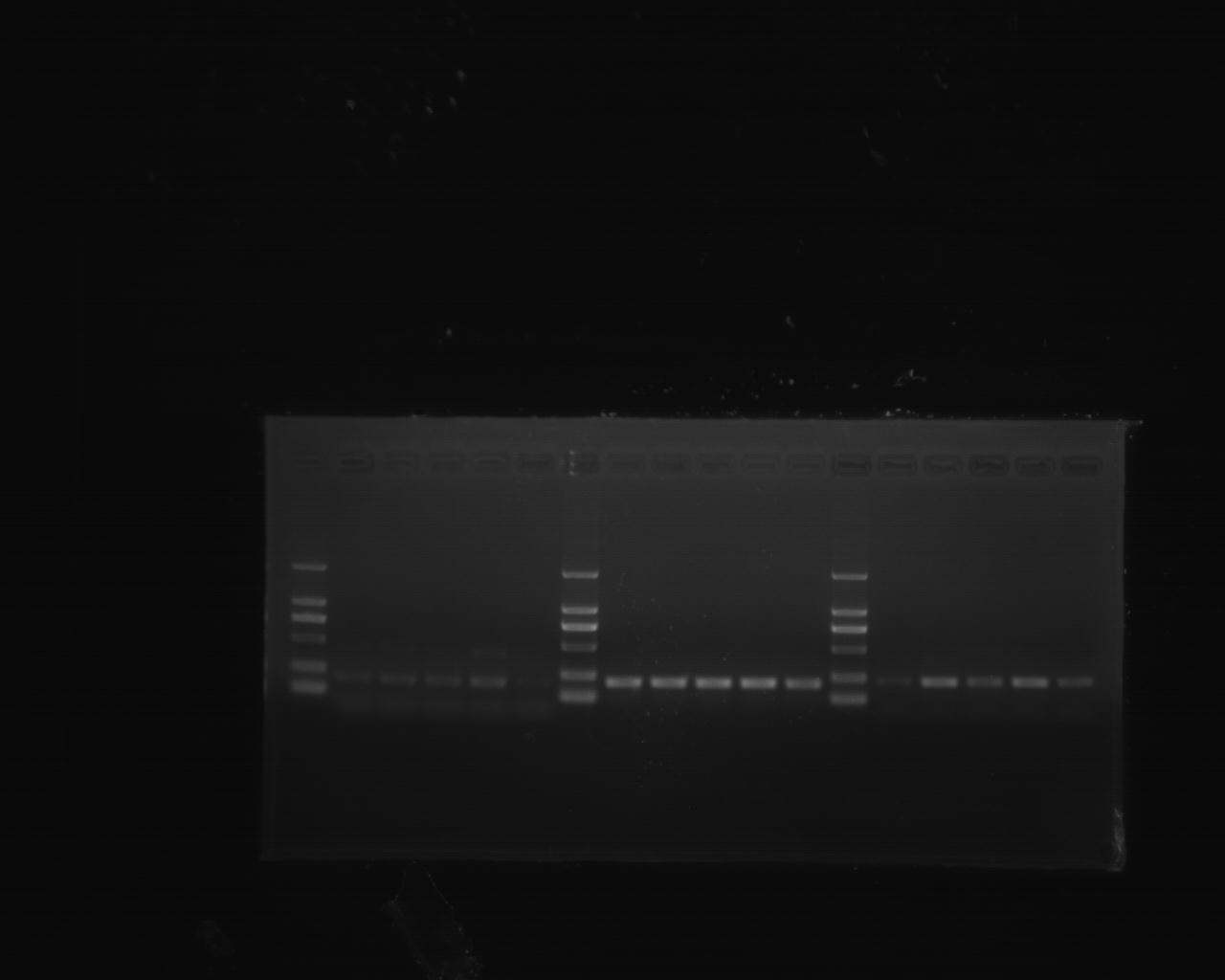


Root Branch Leaves Flower Fruit

*ZjbZIP6*

Root Branch Leaves Flower Fruit

***ZjbZIP9***


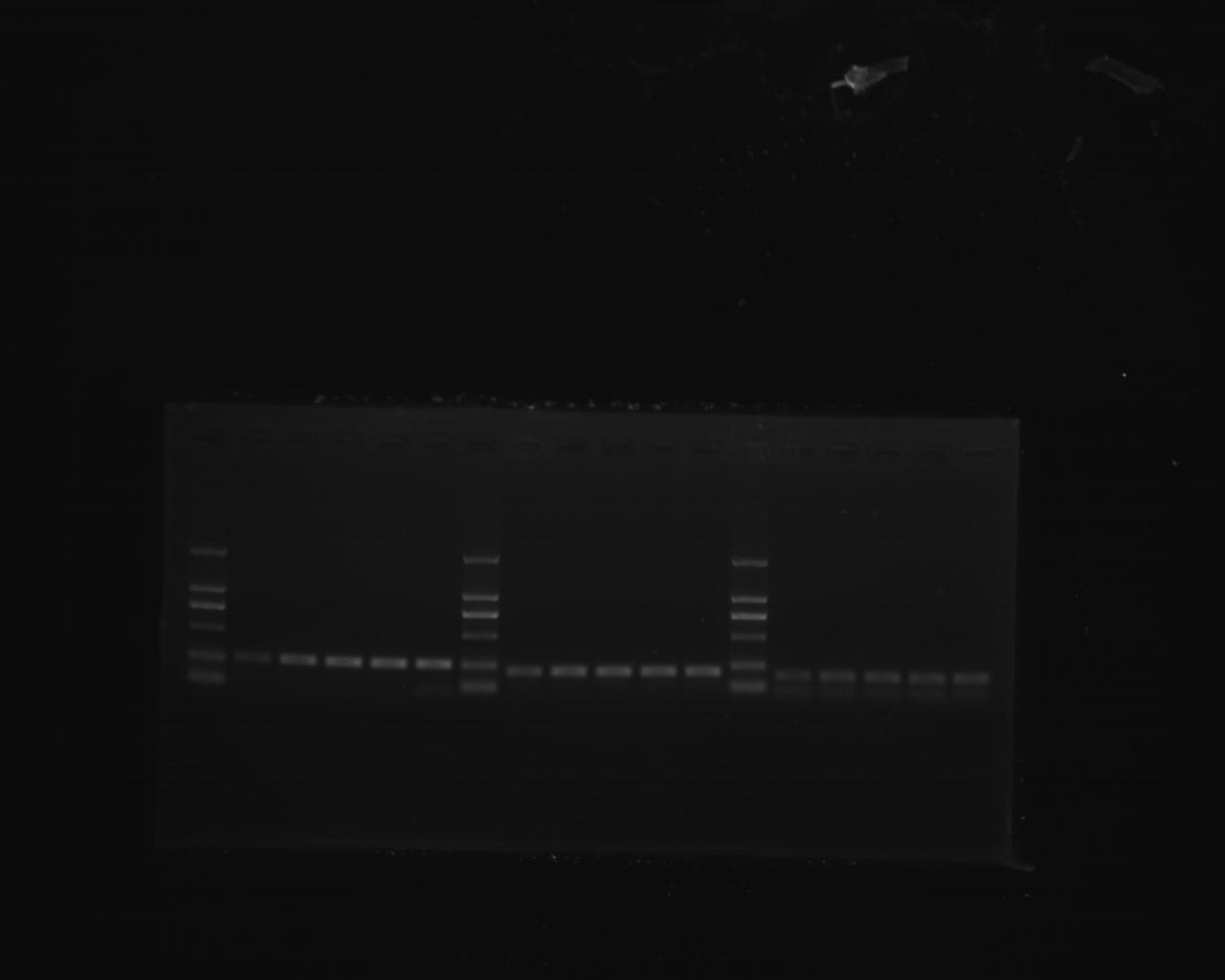


Root Branch Leaves Flower Fruit

*ZjbZIP10*


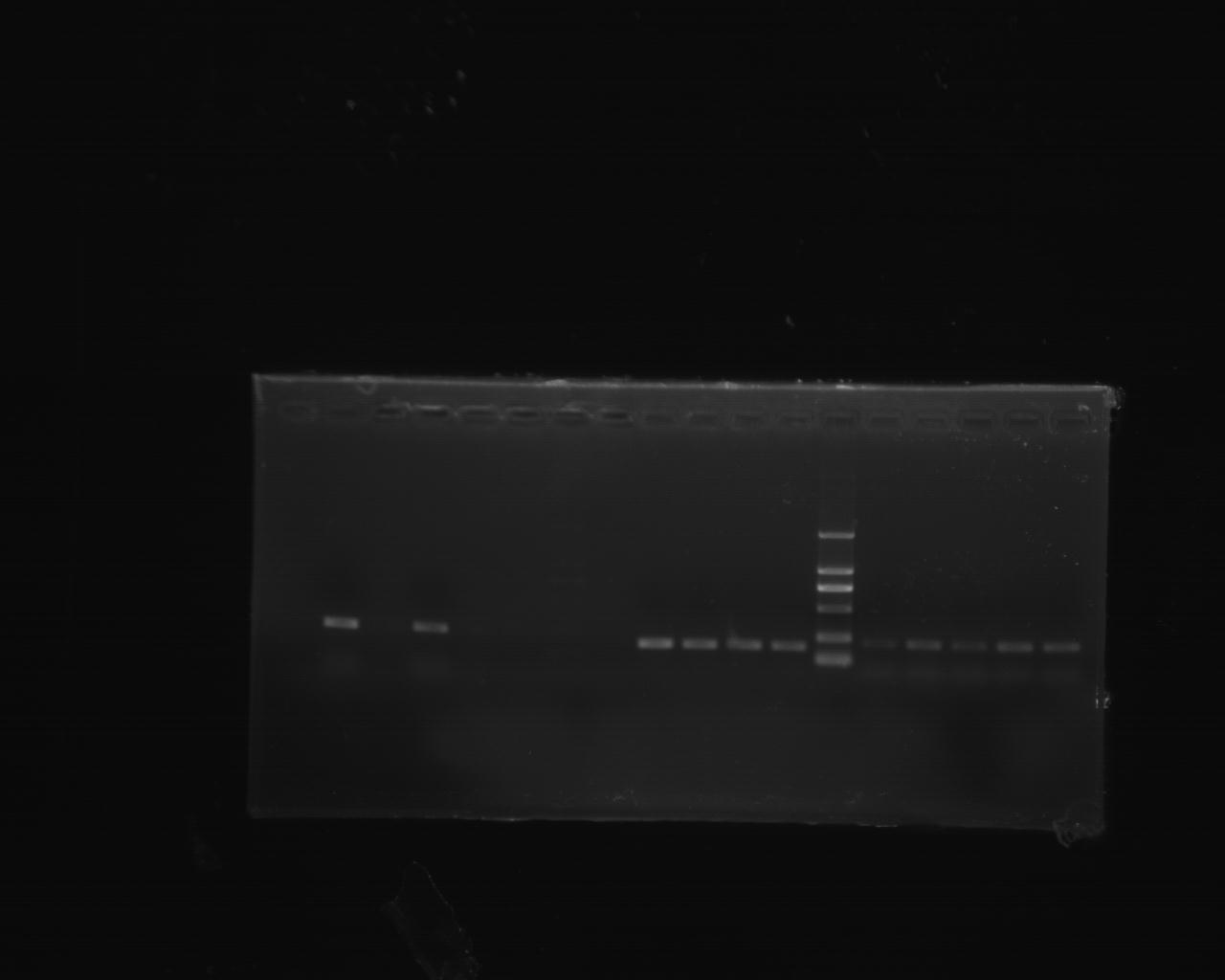


Root Branch Leaves Flower Fruit

ZjbZIP11


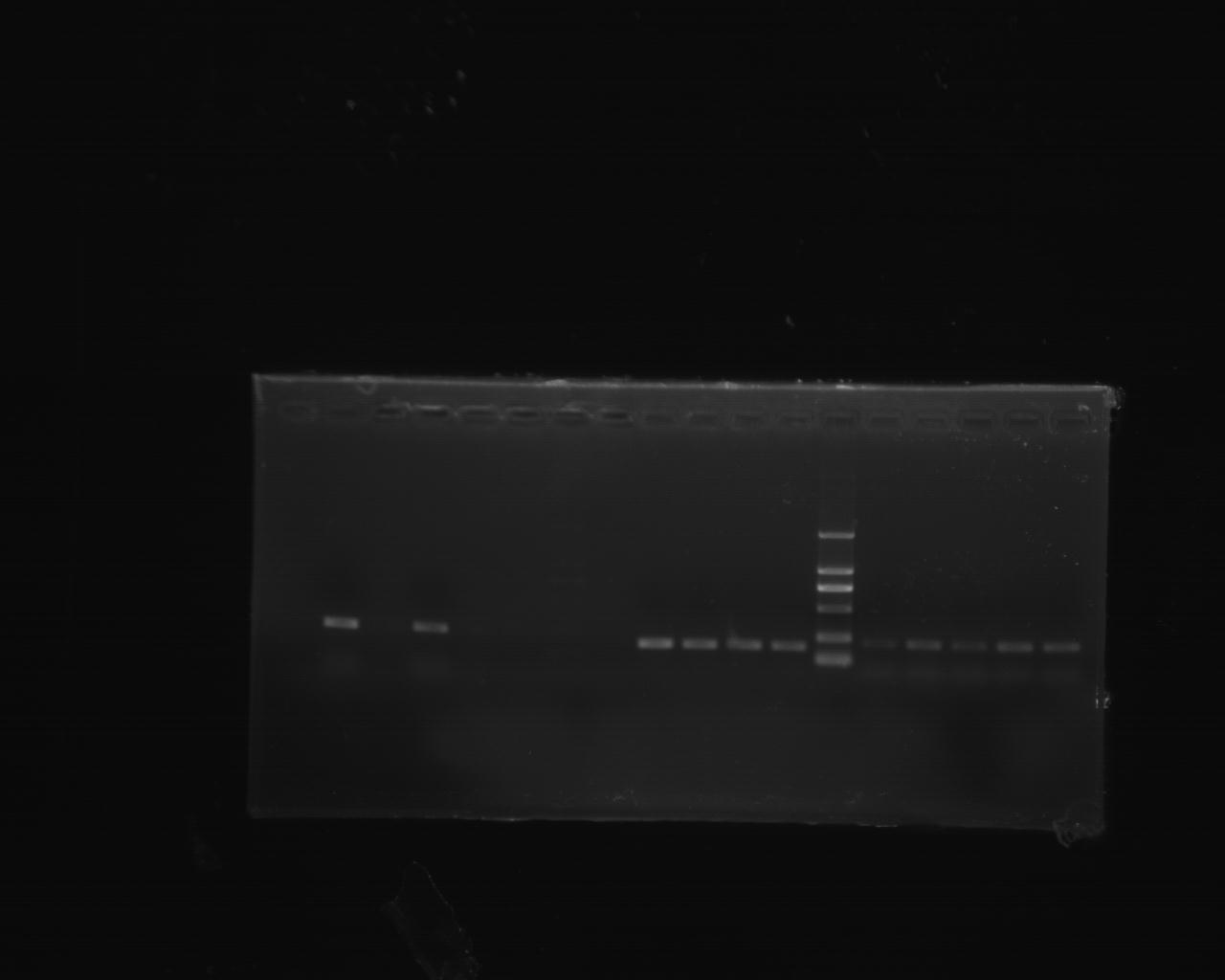


Root Branch Leaves Flower Fruit

ZjbZIP12


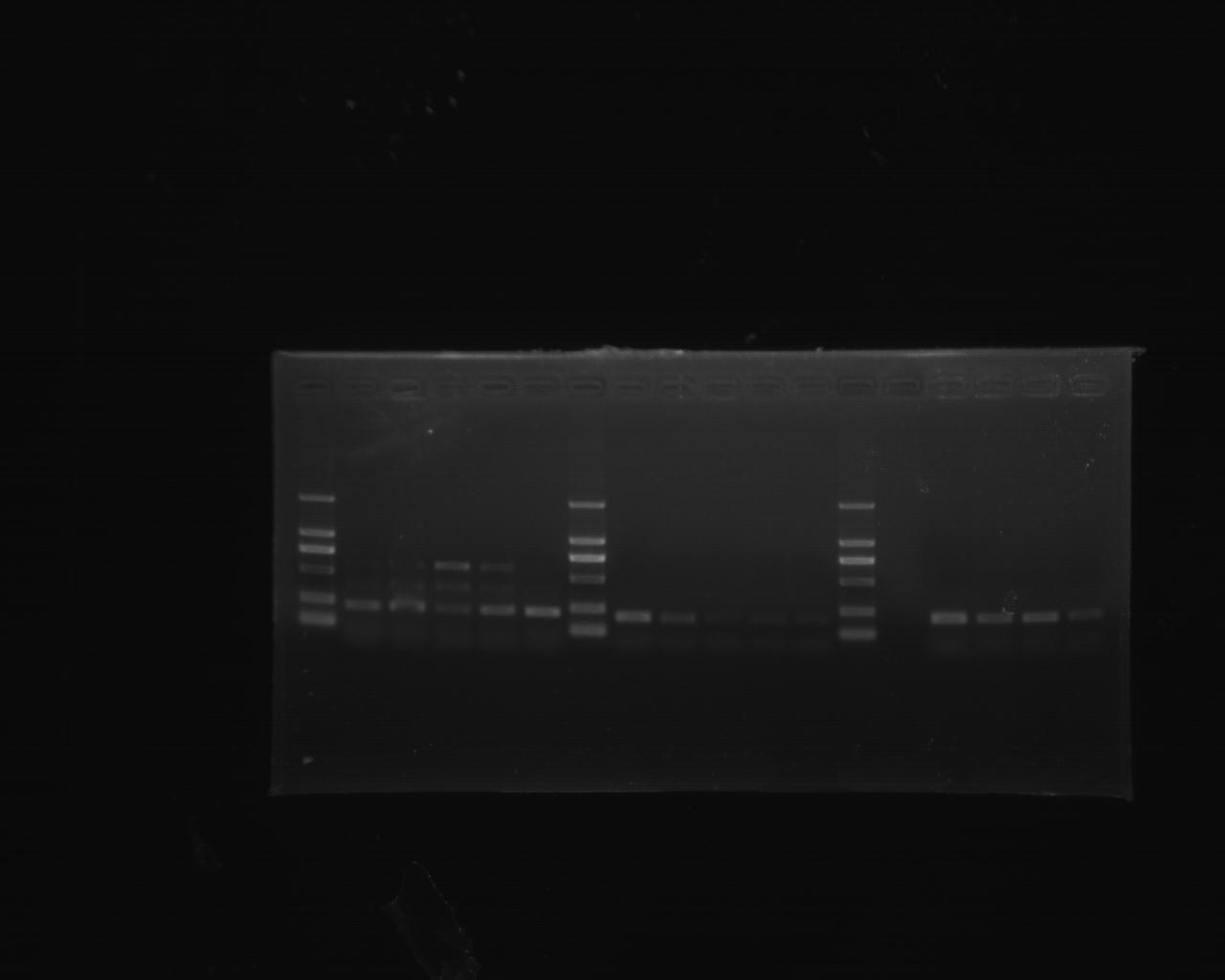


Root Branch Leaves Flower Fruit

ZjbZIP41


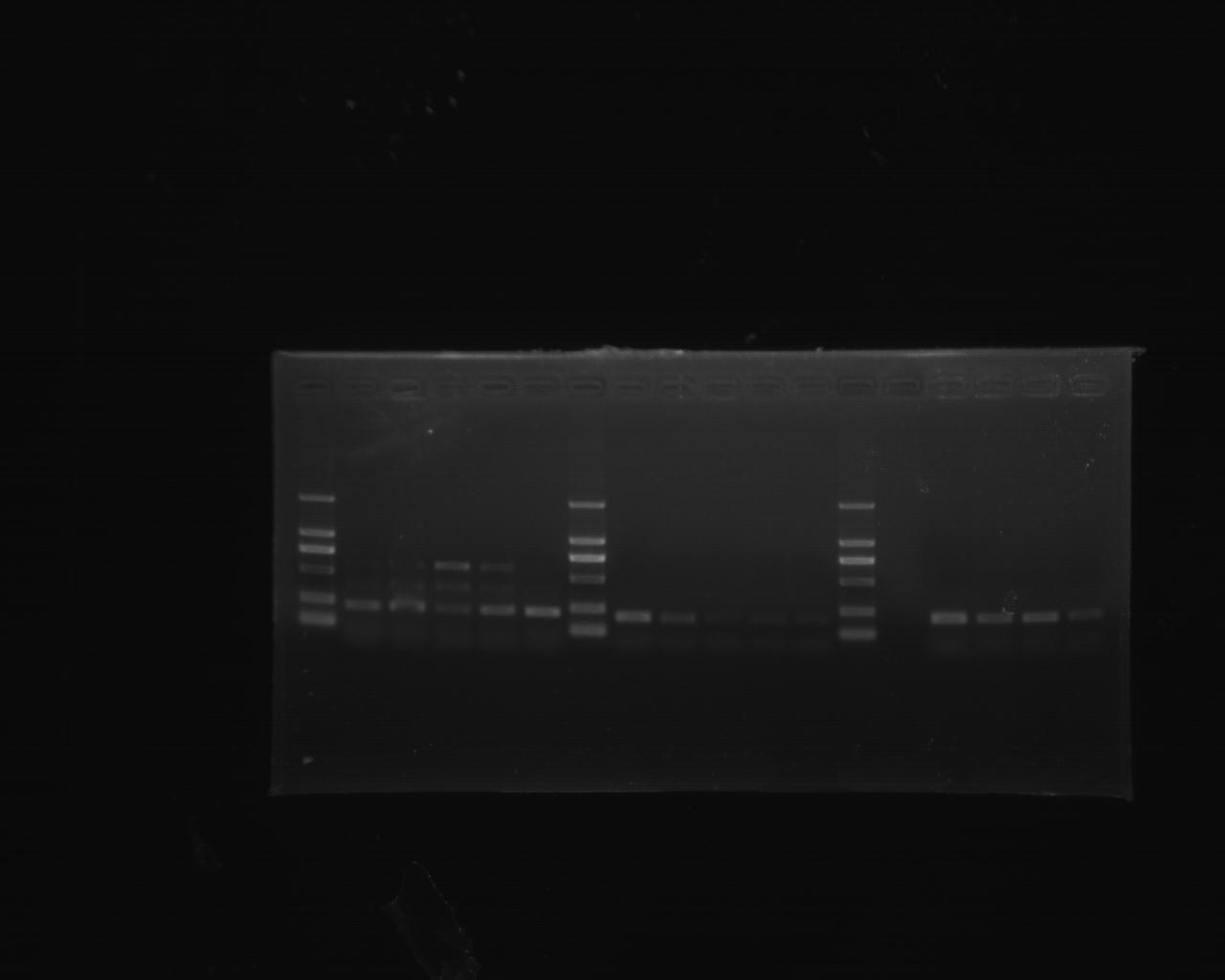


Root Branch Leaves Flower Fruit

ZjbZIP15


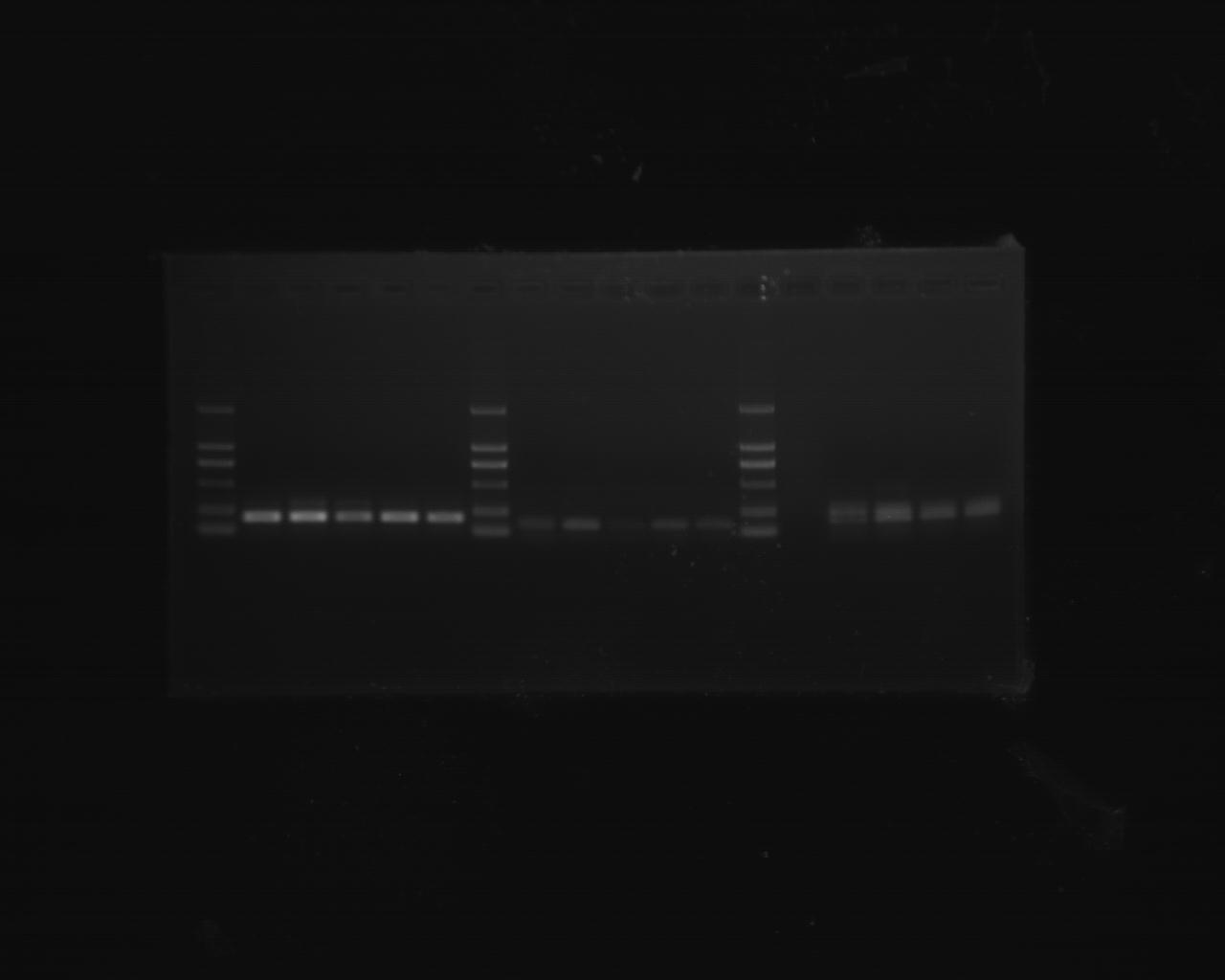


Root Branch Leaves Flower Fruit

ZjbZIP19


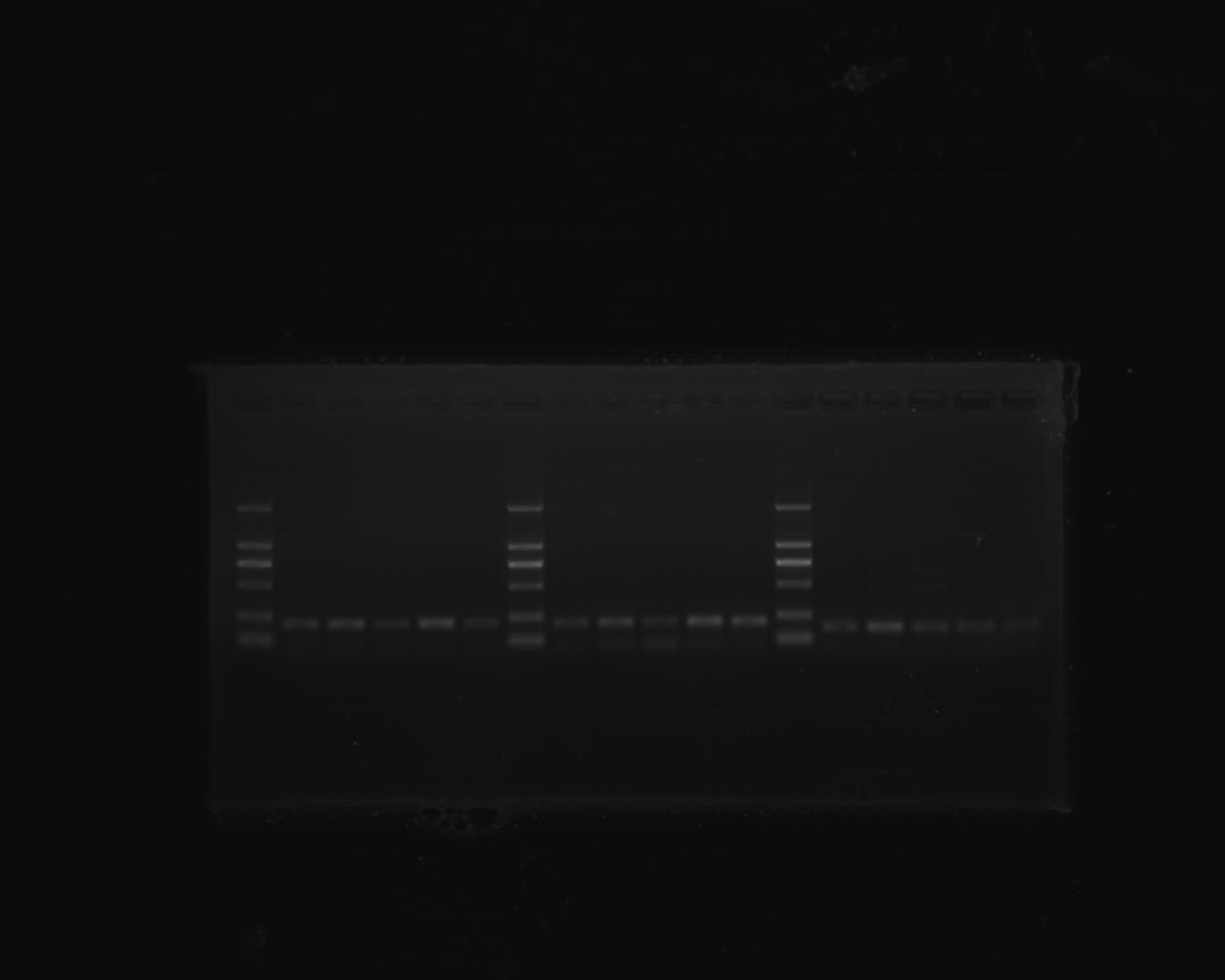


Root Branch Leaves Flower Fruit

ZjbZIP20


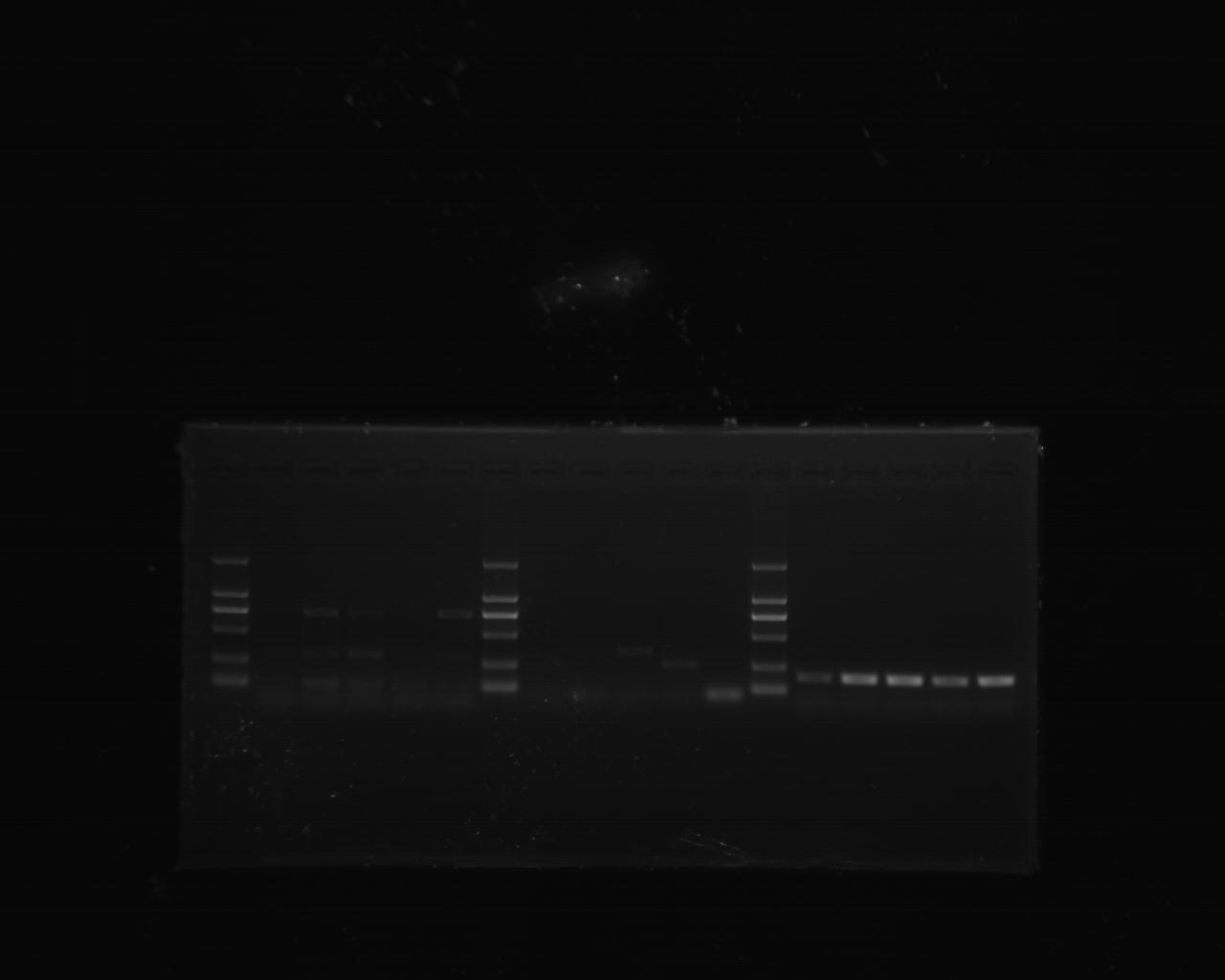


Root Branch Leaves Flower Fruit

ZjbZIP22


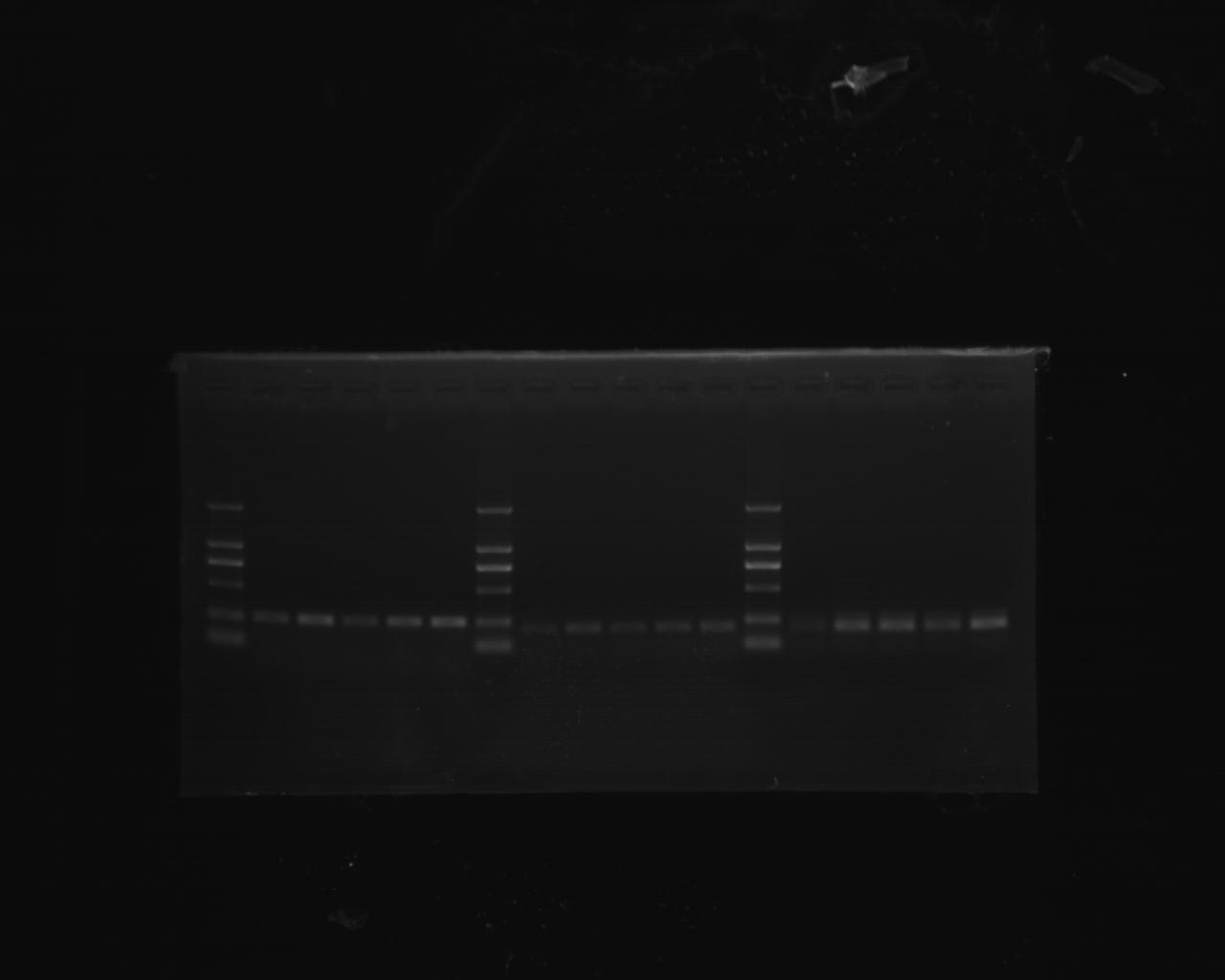


Root Branch Leaves Flower Fruit

ZjbZIP24


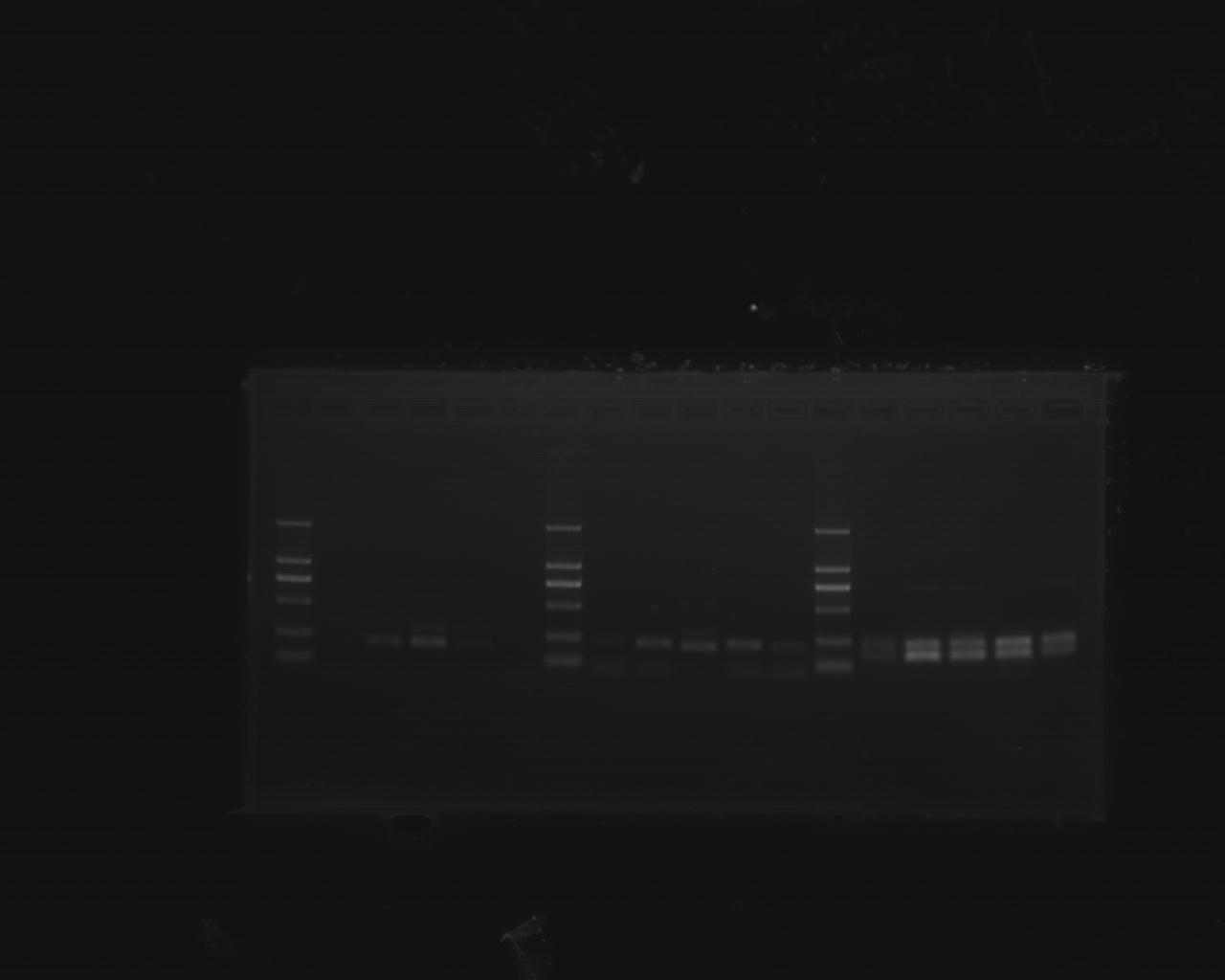


Root Branch Leaves Flower Fruit

ZjbZIP25


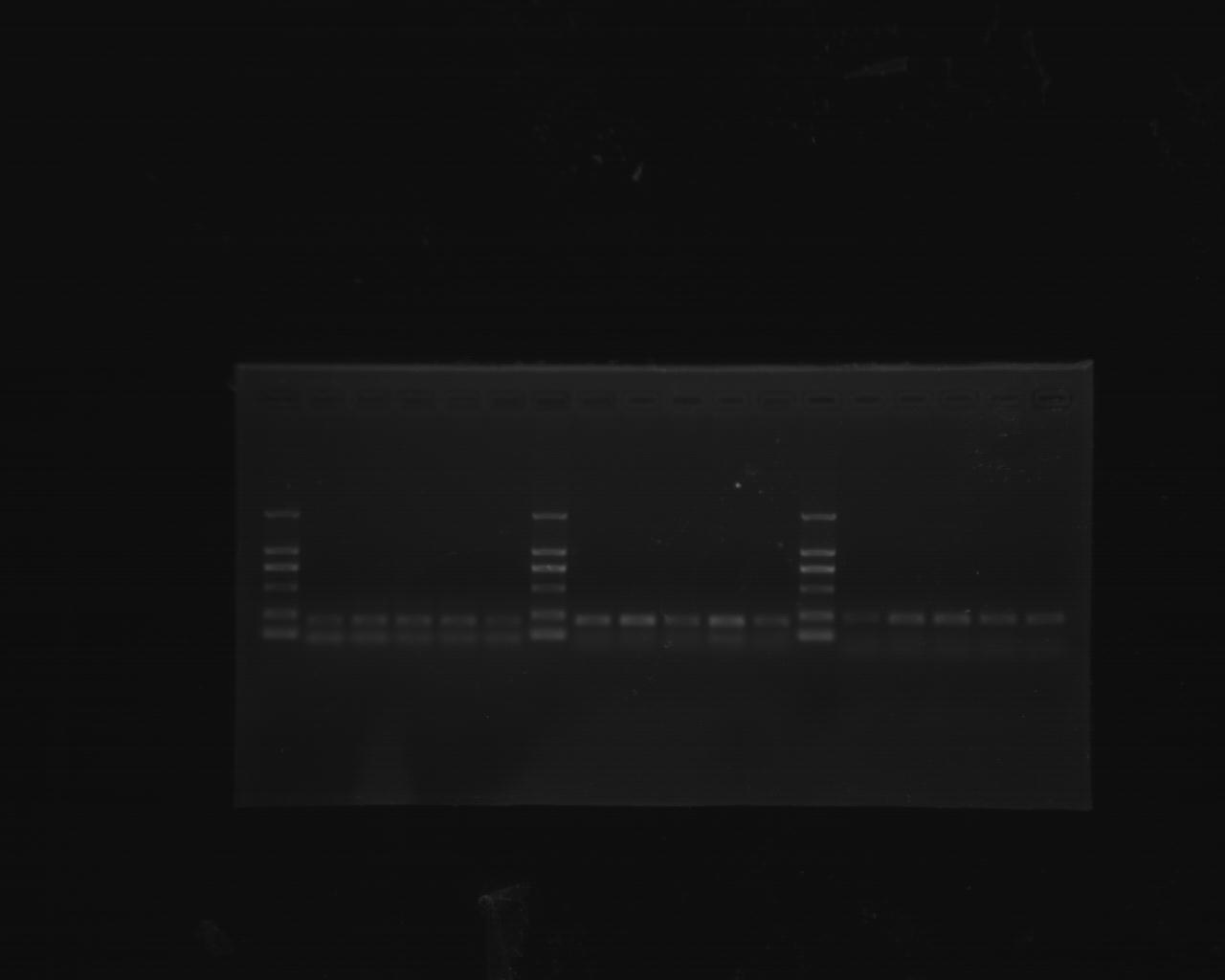


Root Branch Leaves Flower Fruit

ZjbZIP26


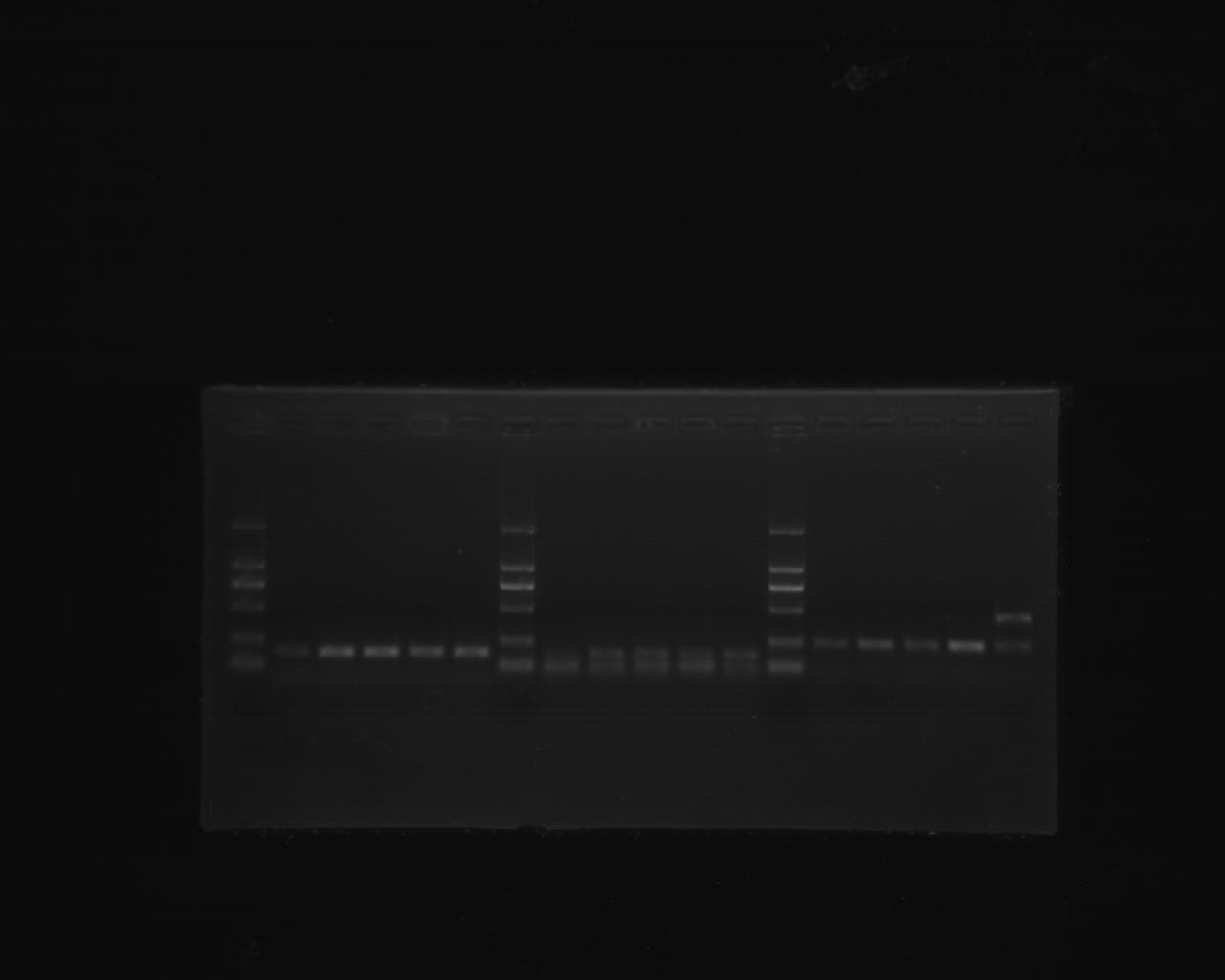


Root Branch Leaves Flower Fruit

ZjbZIP27


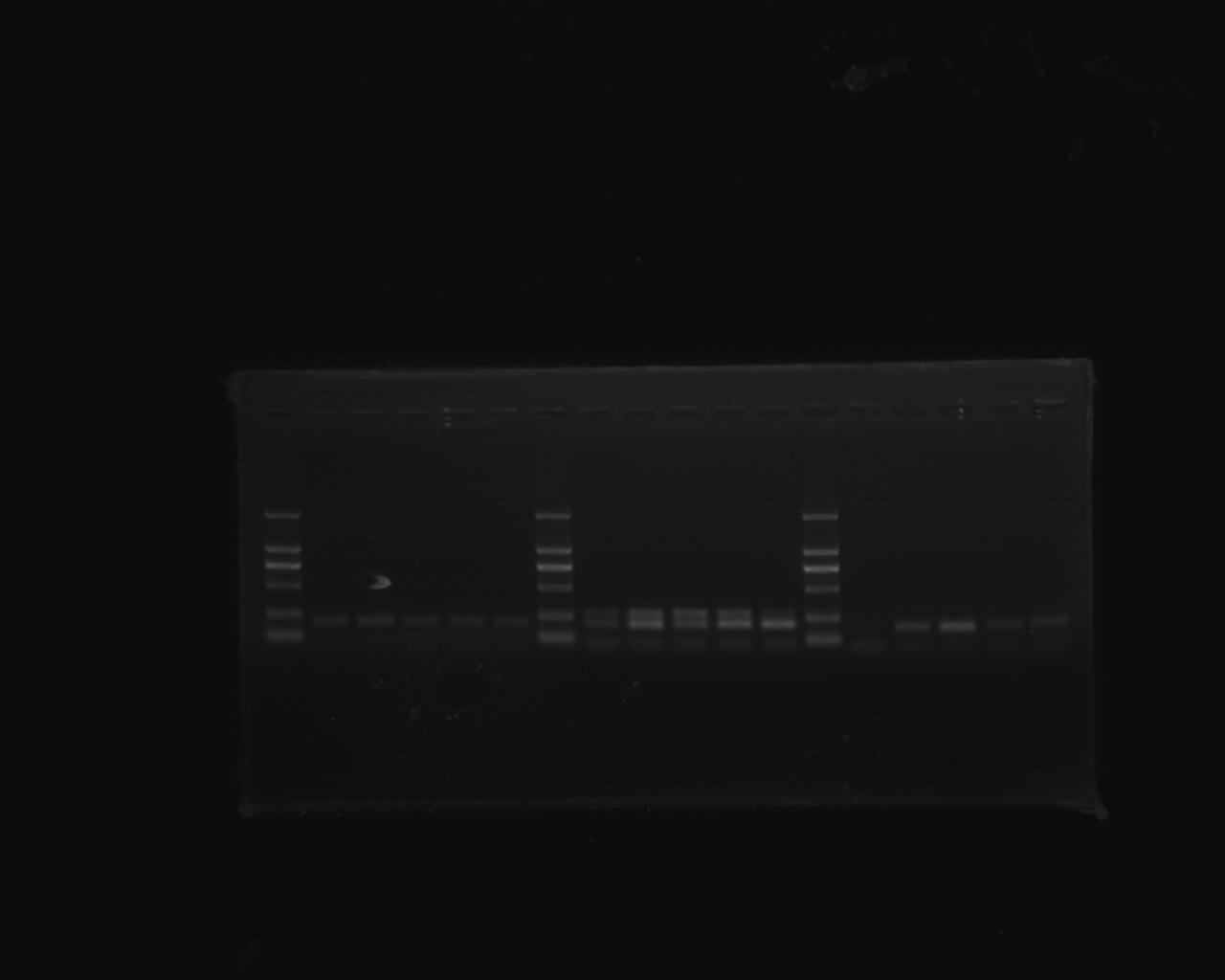


ZjbZIP28

Root Branch Leaves Flower Fruit


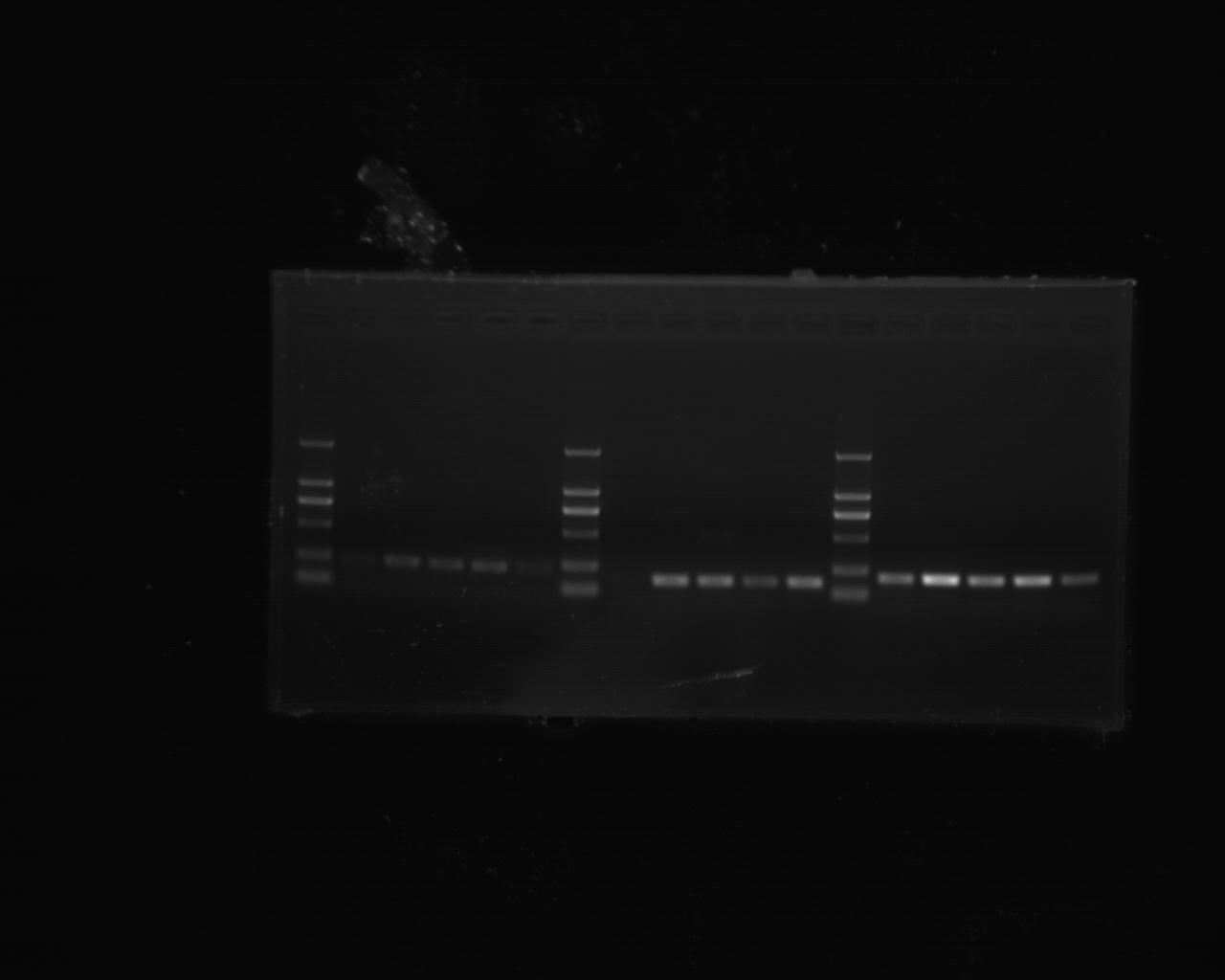


Root Branch Leaves Flower Fruit

ZjbZIP29


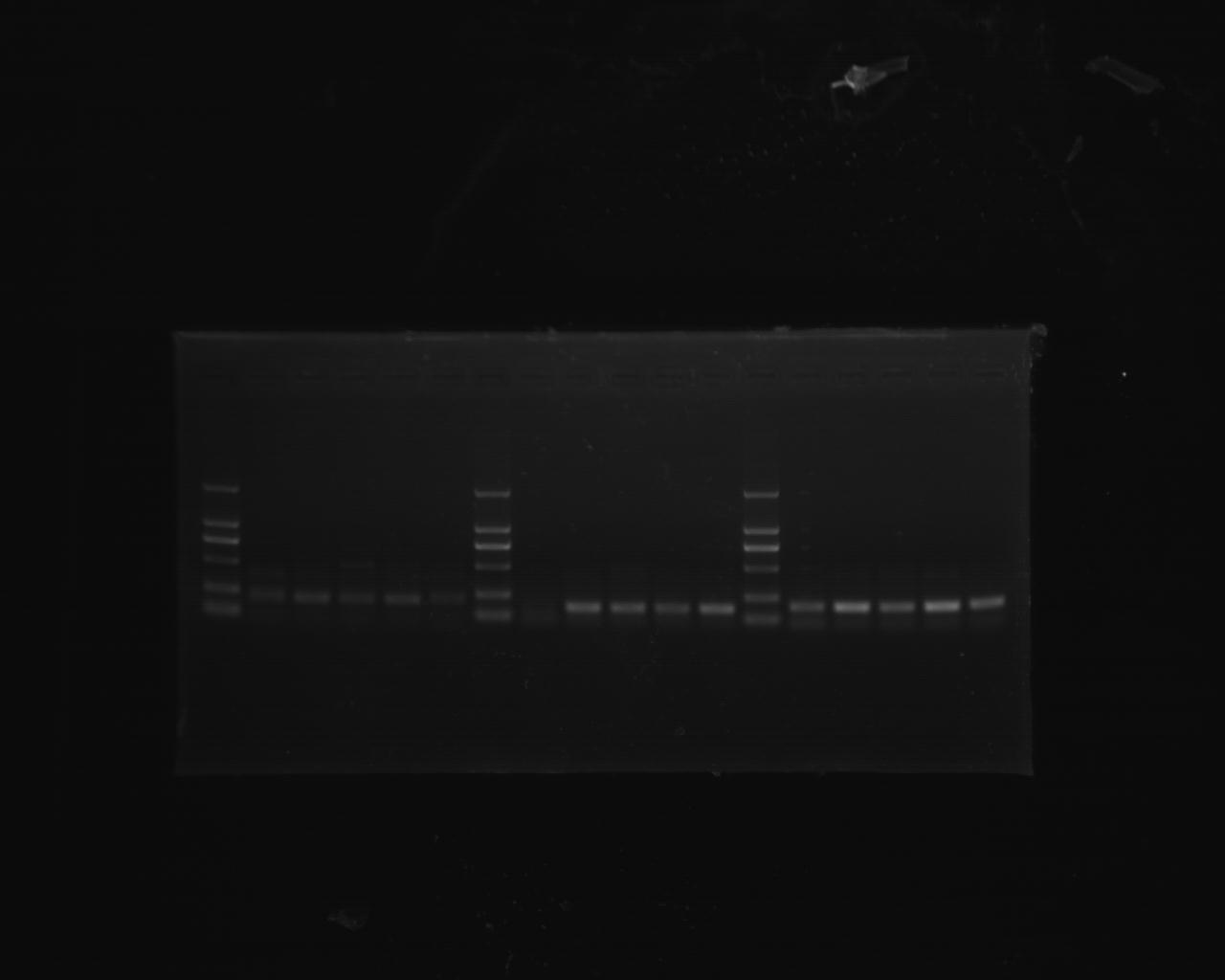


ZjbZIP30

Root Branch Leaves Flower Fruit


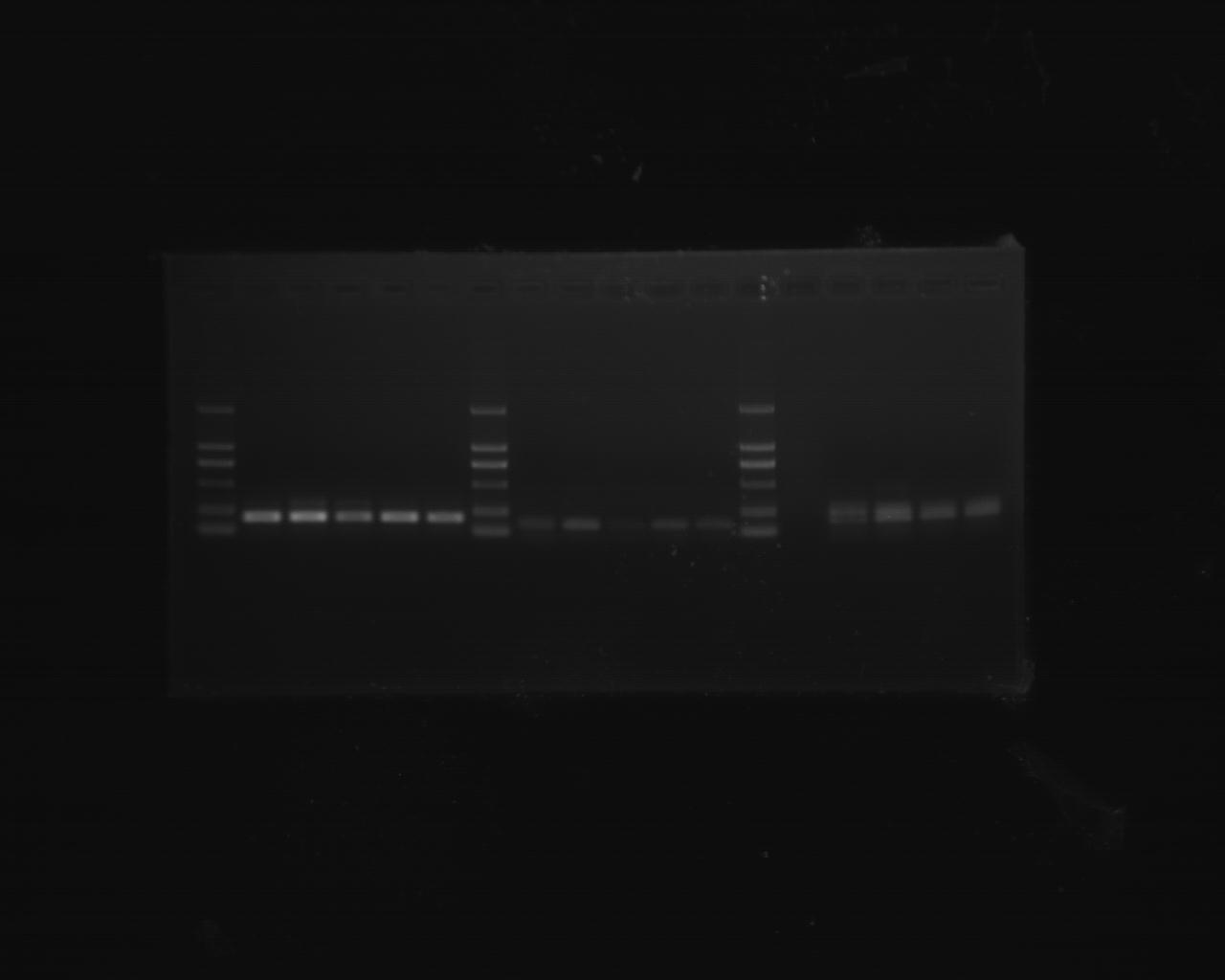


*ZjbZIP31*

Root Branch Leaves Flower Fruit


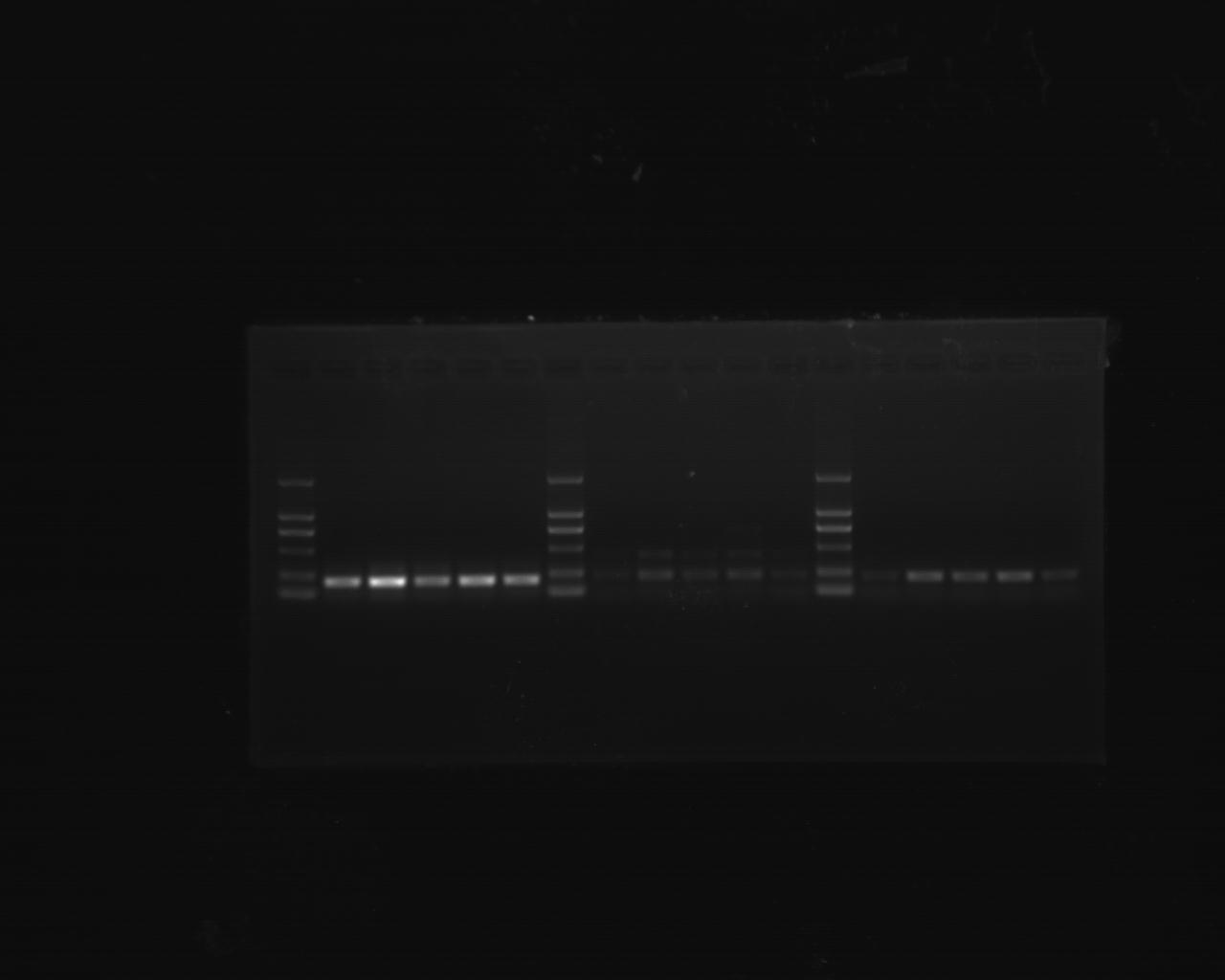


*ZjbZIP33*

Root Branch Leaves Flower Fruit


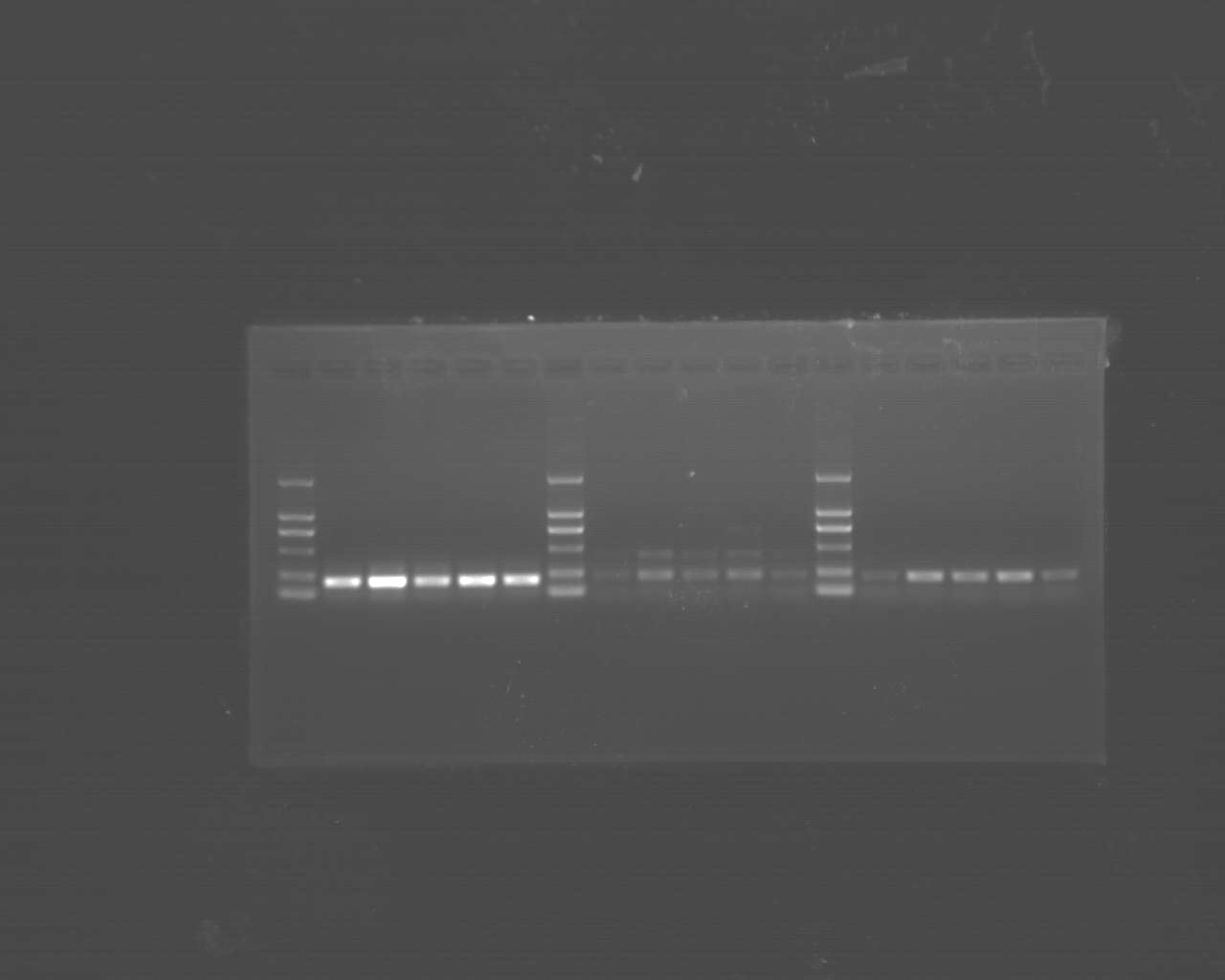


*ZjbZIP34*

Root Branch Leaves Flower Fruit


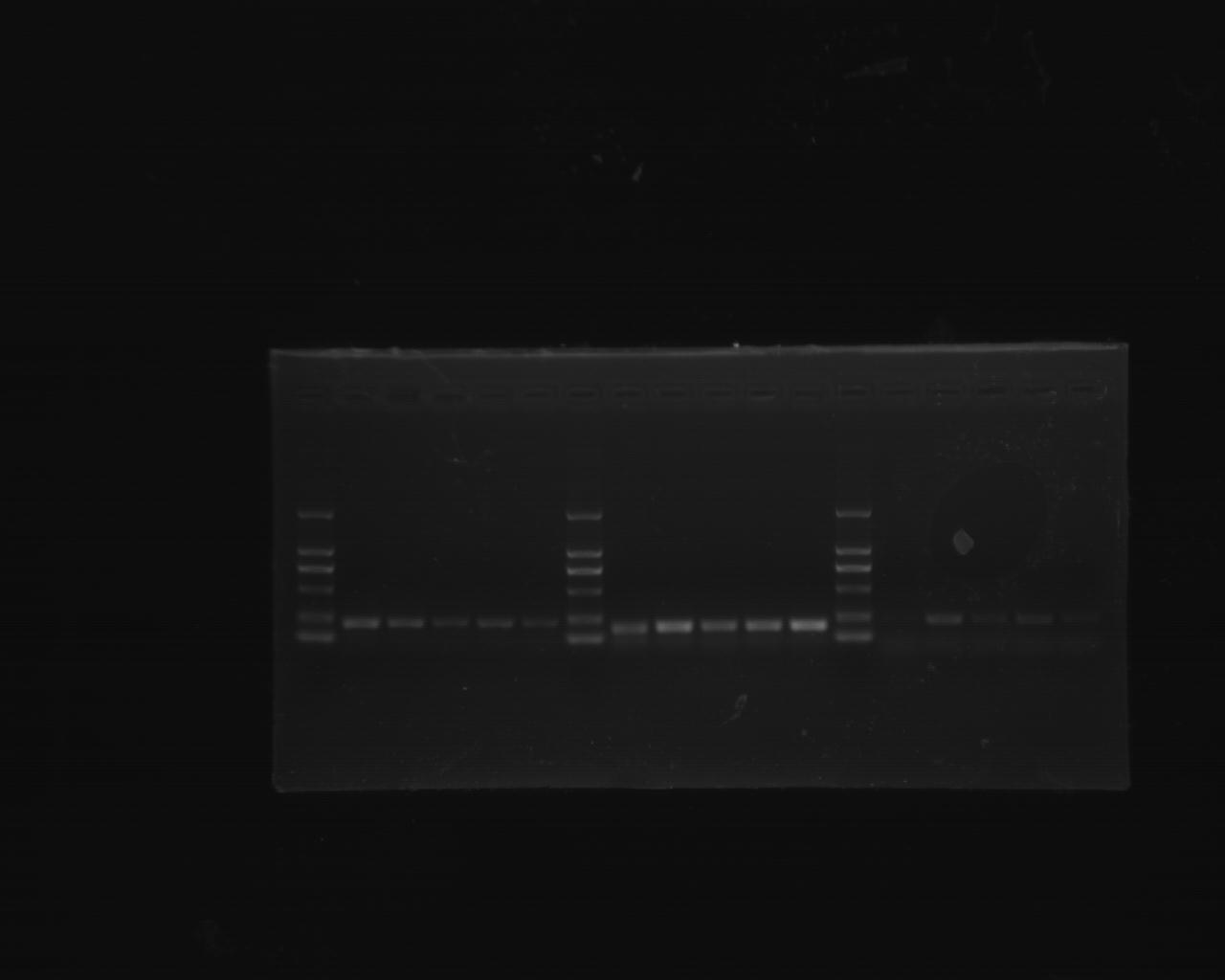


Root Branch Leaves Flower Fruit

*ZjbZIP35*


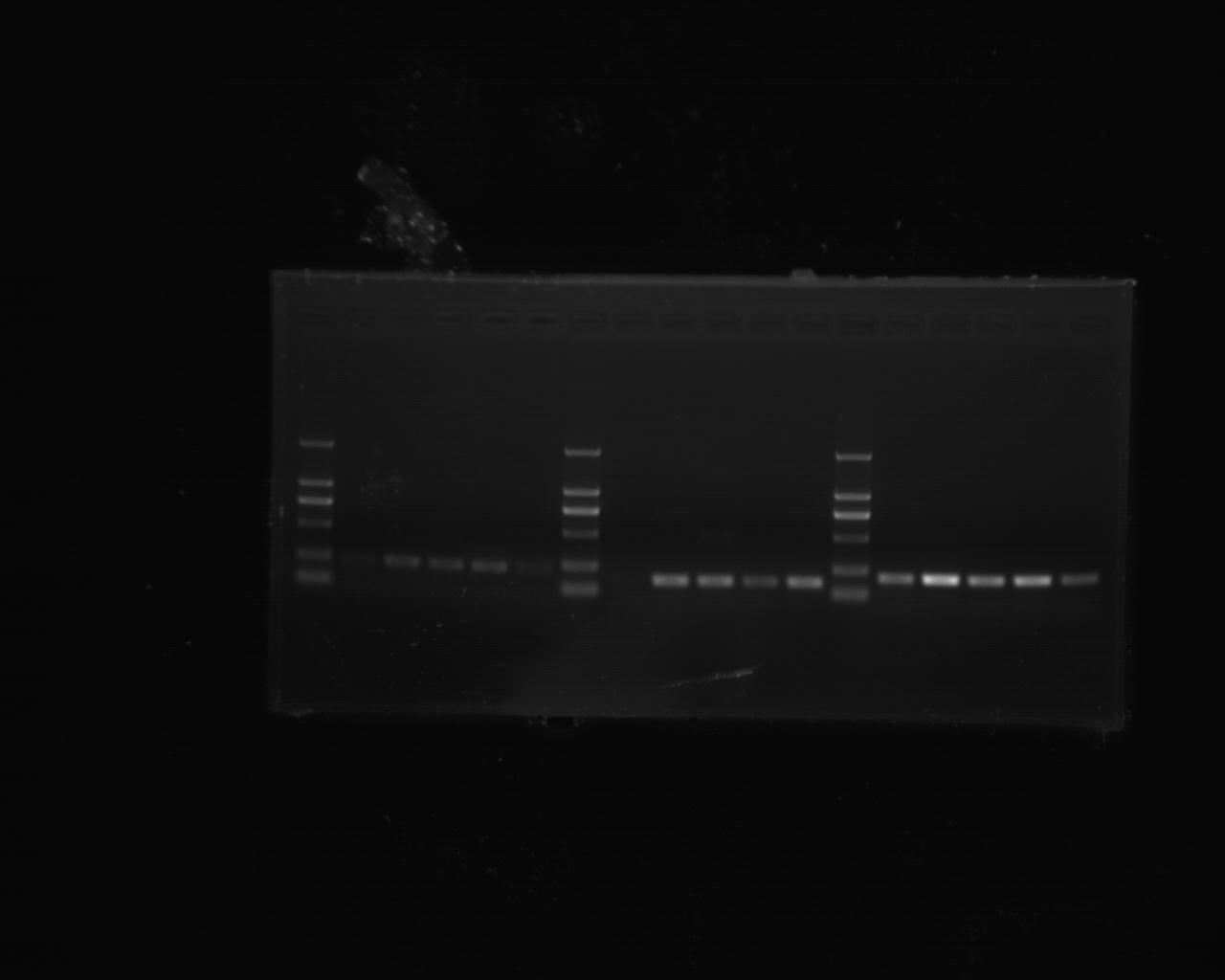


Root Branch Leaves Flower Fruit

*ZjbZIP36*


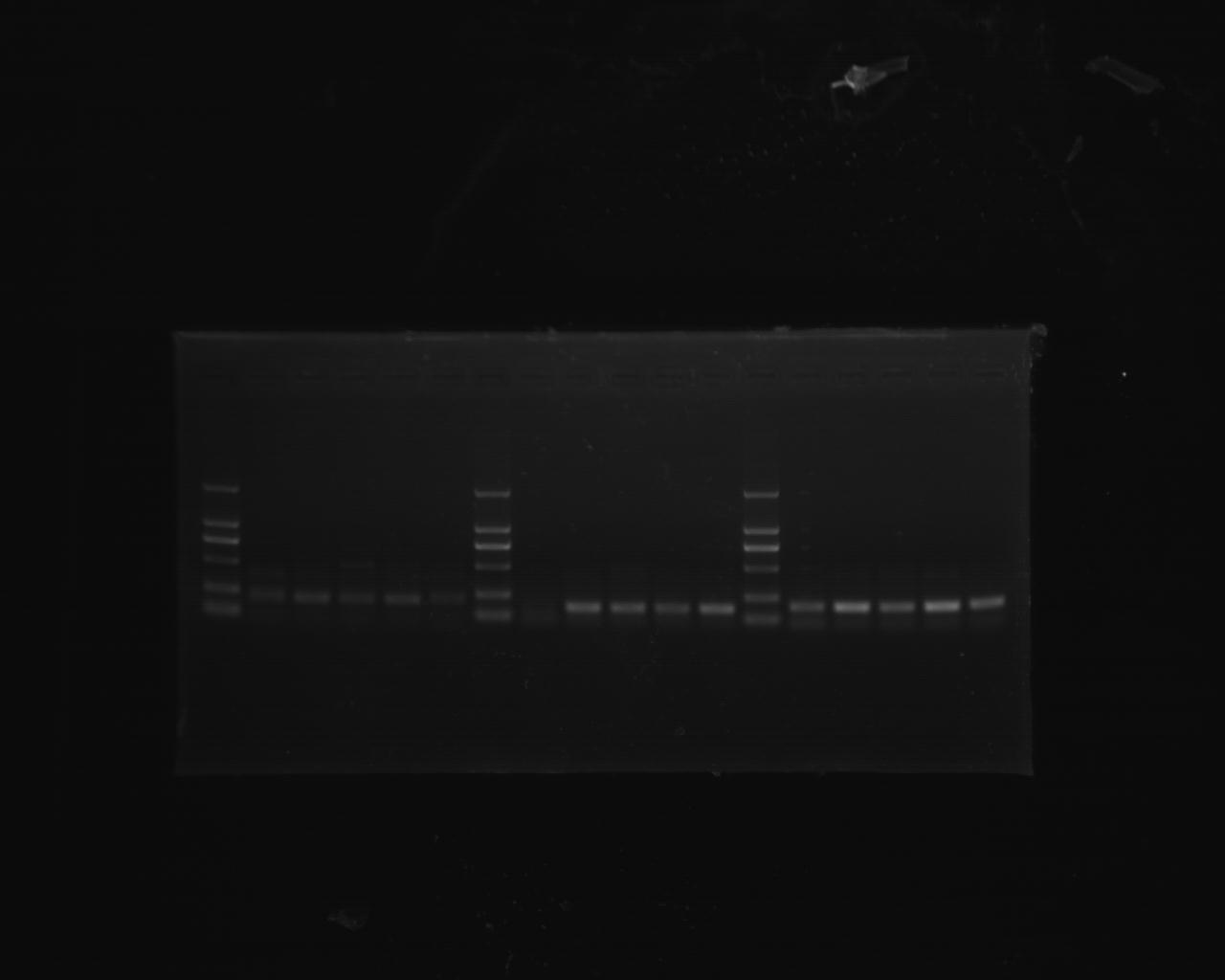


Root Branch Leaves Flower Fruit

*ZjbZIP38*


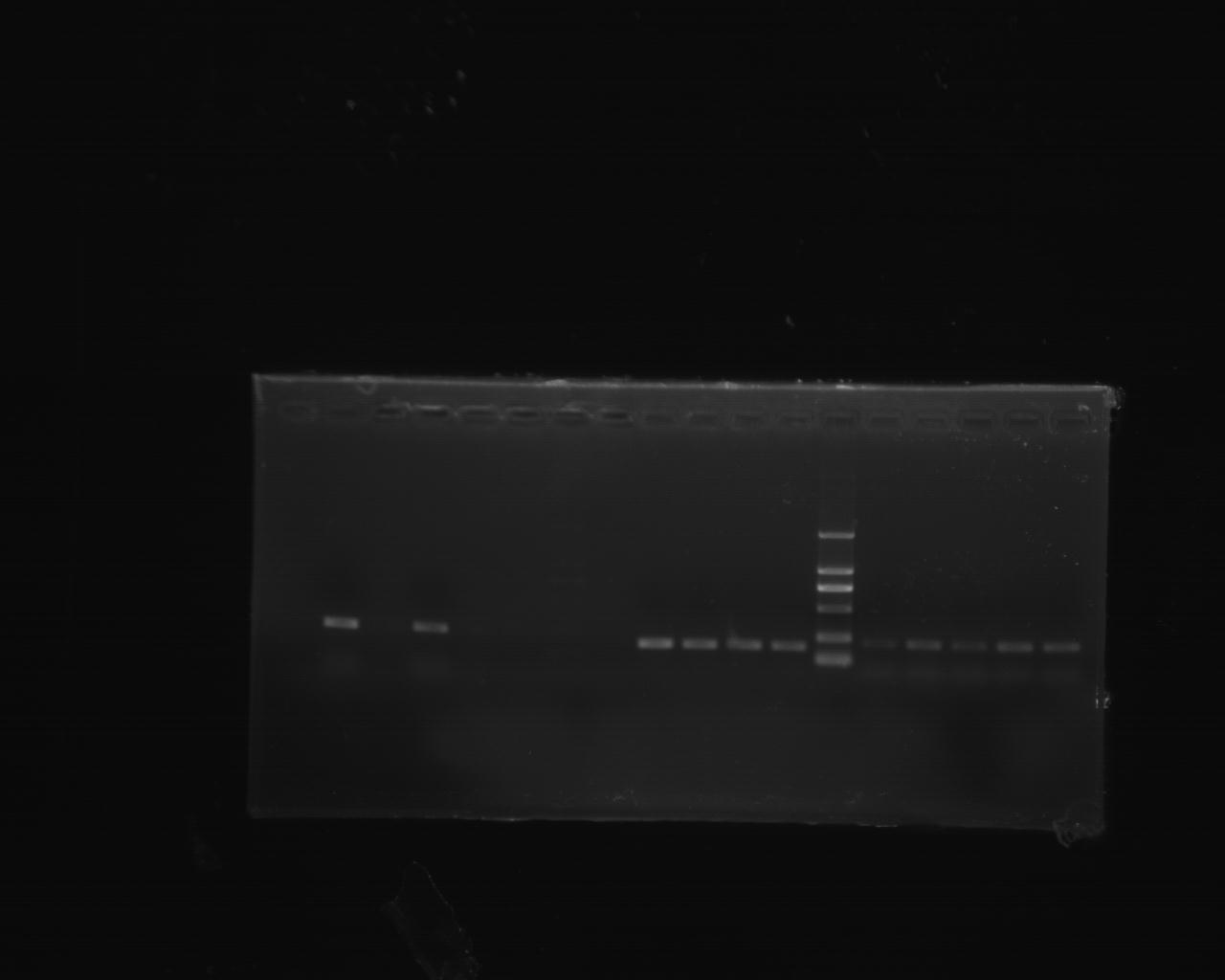


Root Branch Leaves Flower Fruit

*ZjbZIP40*


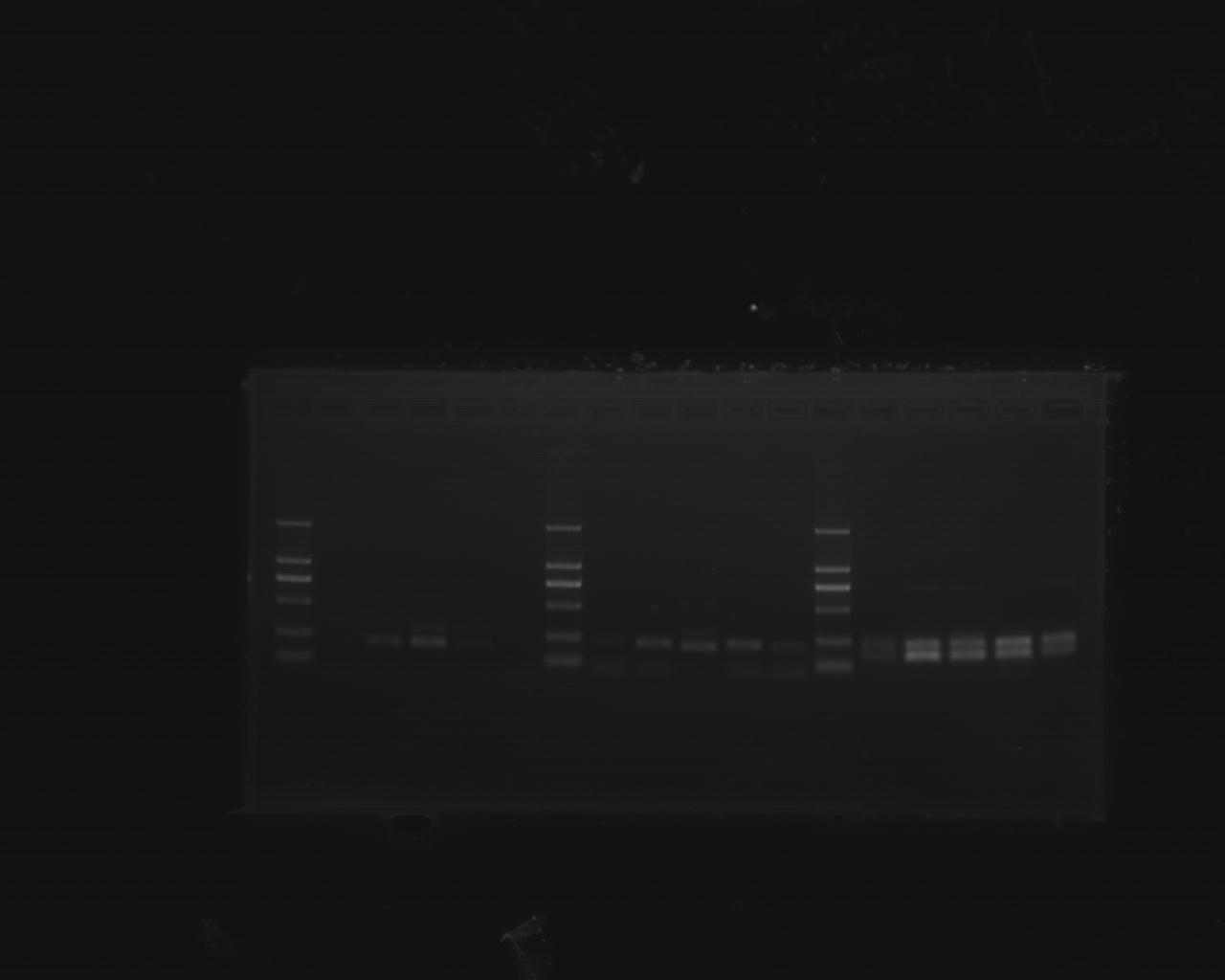


*ZjbZIP42*

Root Branch Leaves Flower Fruit


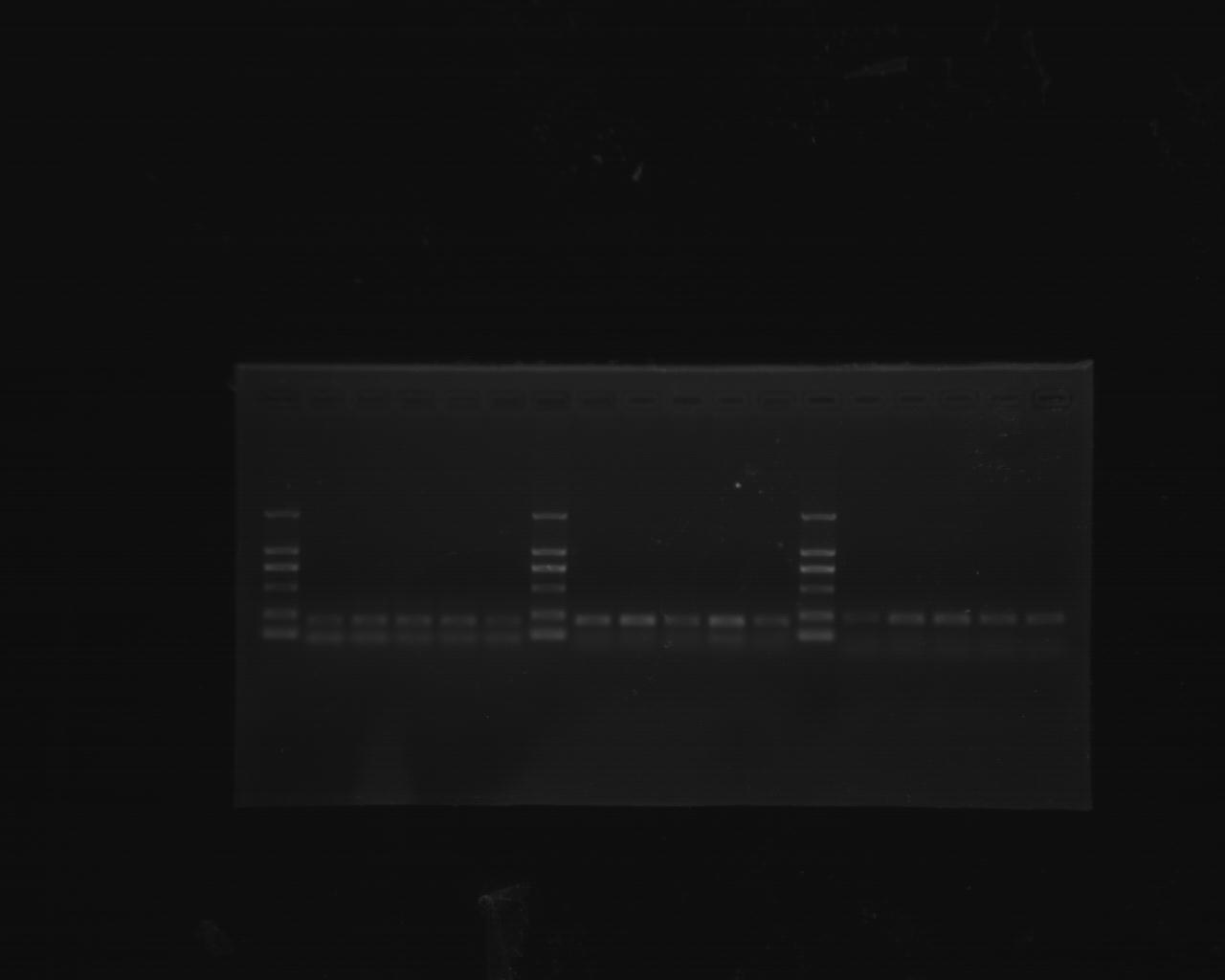


*ZjbZIP45*

Root Branch Leaves Flower Fruit

B. All original, blot images in Fig. 7B were now as the following.

-LW


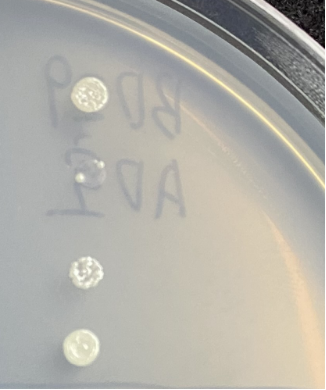

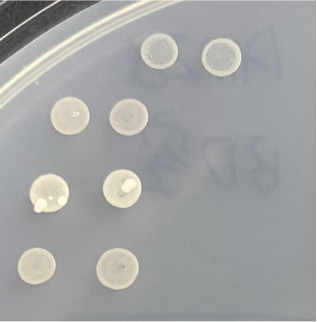

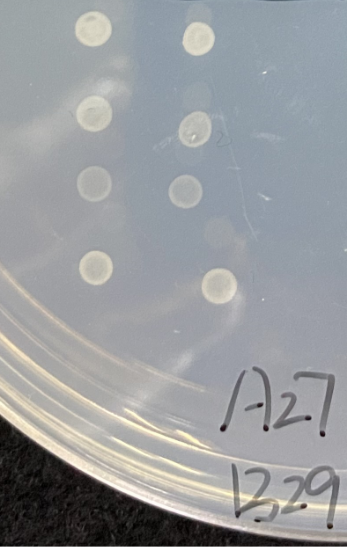

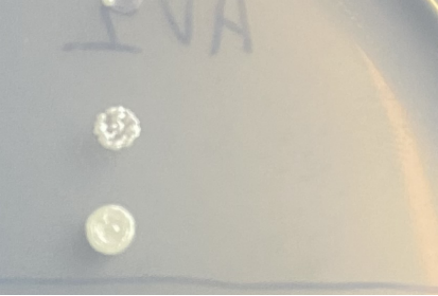

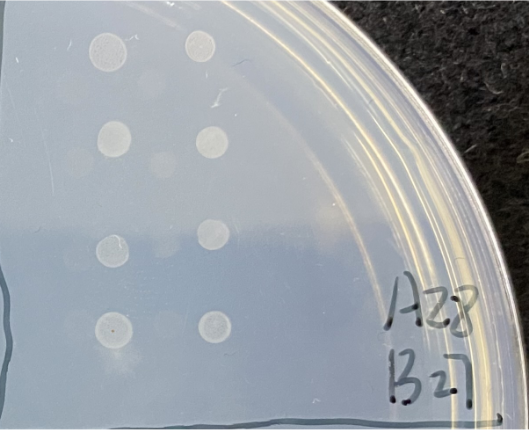

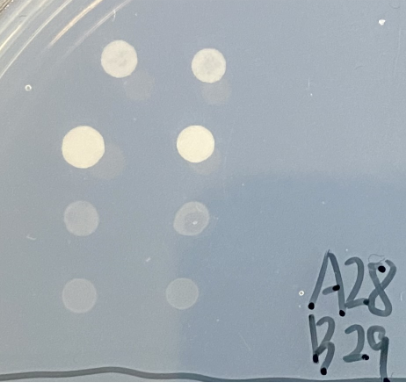


Empty+ZjbZIP29

ZjbZIP27+Empty

ZjbZIP27+ZjbZIP29

ZjbZIP28+Empty

ZjbZIP28+ZjbZIP27

ZjbZIP28+ZjbZIP29

-LWH


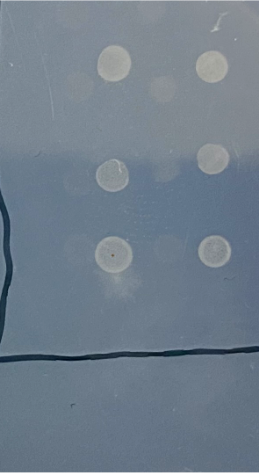

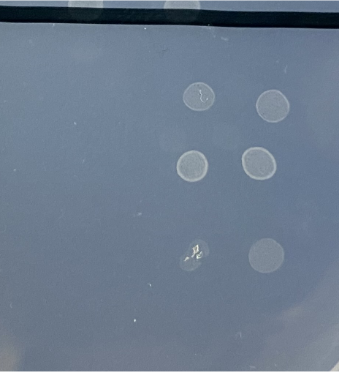

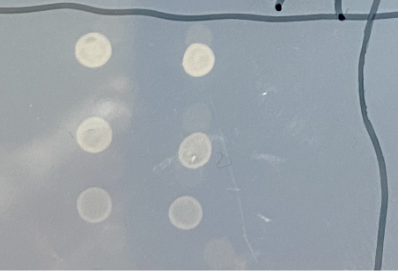

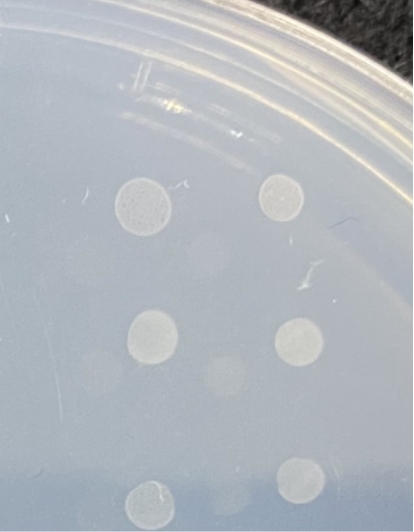

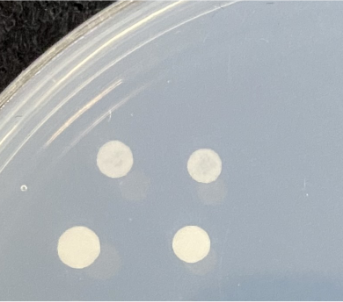

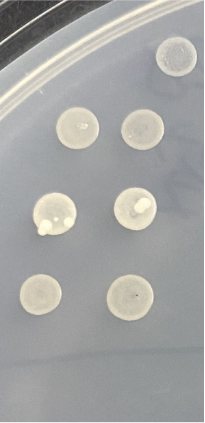


Empty+ZjbZIP29

ZjbZIP27+Empty

ZjbZIP27+ZjbZIP29

ZjbZIP28+ZjbZIP27

ZjbZIP28+ZjbZIP29

ZjbZIPA28+Empty


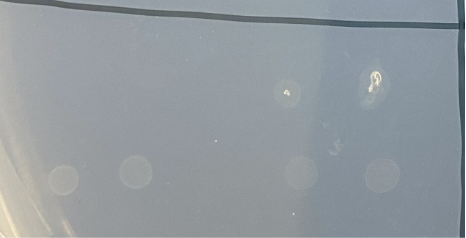

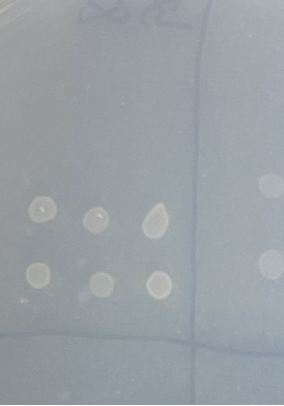

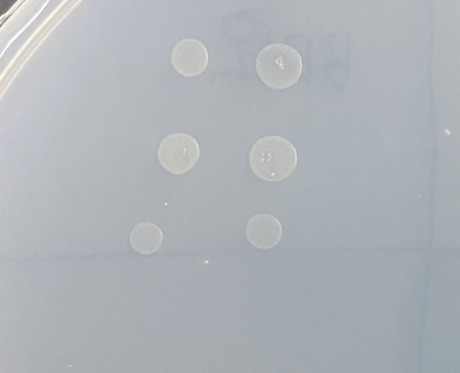

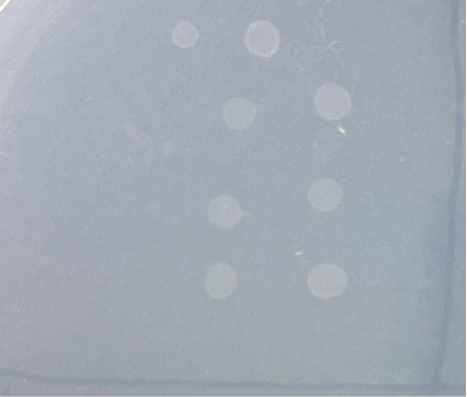

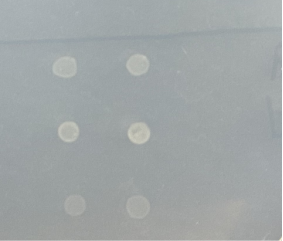

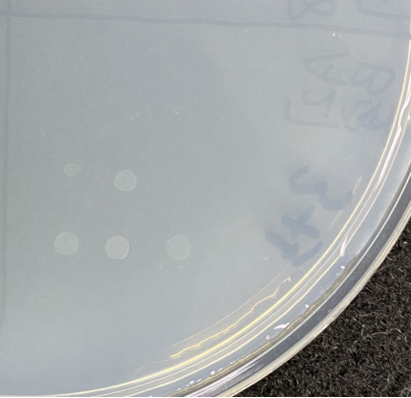


Empty+ZjbZIP29

-LWH+3AT

ZjbZIP27+Empty

ZjbZIP27+ZjbZIP29

ZjbZIP28+Empty

ZjbZIP28+ZjbZIP27

ZjbZIP28+ZjbZIP29

**-LWAH**


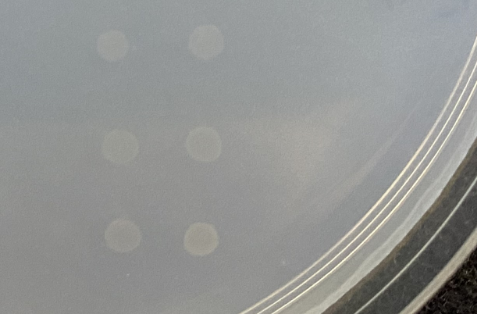

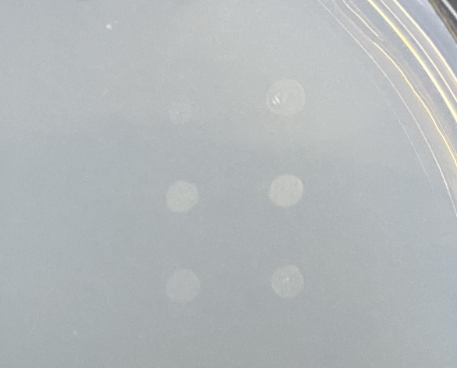

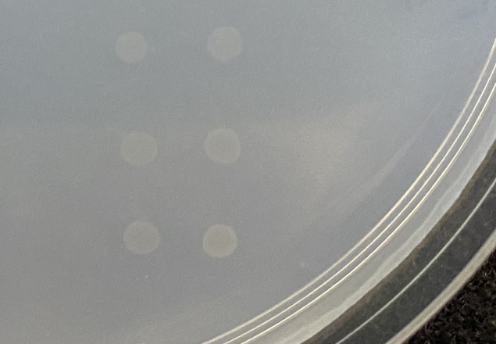

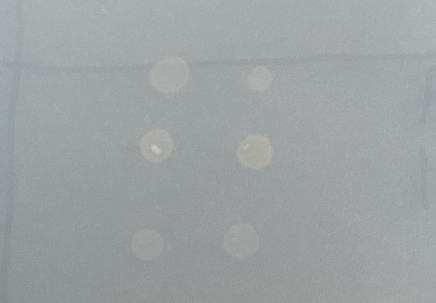


Empty+ZjbZIP29

ZjbZIP27+Empty


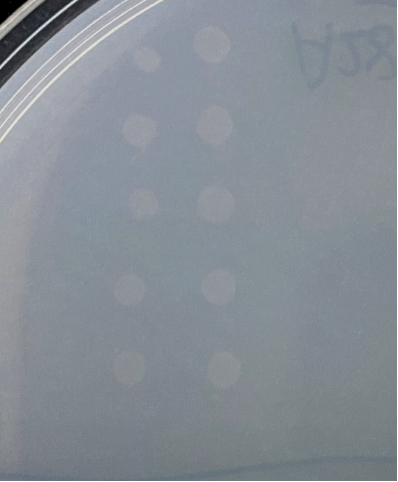

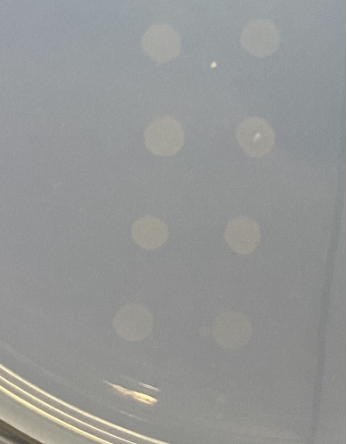


ZjbZIP27+ZjbZIP29

ZjbZIP28+Empty

ZjbZIP28+ZjbZIP27

ZjbZIP28+ZjbZIP29
